# Interfacial Energetics in Metal Halide Perovskite Solar Cells

**DOI:** 10.1021/acs.chemrev.5c00782

**Published:** 2026-08-26

**Authors:** Zuzanna Molenda, Lucas Scalon, Shivam Singh, Yana Vaynzof

**Affiliations:** † Chair for Emerging Electronic Technologies, Dresden University of Technology, Nöthnitzer Str. 61, 01187 Dresden, Germany; ‡ 28394 Leibniz Institute for Solid State and Materials Research Dresden, Helmholtzstraße 20, 01069 Dresden, Germany

## Abstract

Controlling the energetic landscape of metal halide perovskite solar cells is crucial for optimizing device efficiency and stability, as well as enabling new architectures beyond conventional layered designs. While band alignment criteria are key determinants of performance in organic photovoltaic devices, they are often insufficient for perovskites due to their high charge carrier density, mobile ions, and soft lattice structure. A deep understanding of the electronic structure of the perovskite and the contacting layers remains an active area of research and requires expertise in the characterization techniques. In this review, we analyze how interfacial energetics influence charge extraction, the formation of energy barriers, interfacial recombination, and ion migration, highlighting the unique challenges posed by the soft ionic lattice of metal halide perovskites. To this end, we provide a comprehensive overview of experimental techniques for probing energetic alignment, emphasizing their operating principles, limitations, and emerging in situ variants. Building on these insights, we discuss strategies for controlling the interfacial energetics of perovskite solar cells, ranging from interfacial layers and dipolar modifiers to electrical doping. Finally, we explore the device architectures that eliminate the need for charge-selective layers. Together, these insights establish a framework for understanding and engineering the energetic landscape of metal halide perovskite-based devices, linking fundamental semiconductor physics with practical strategies for future perovskite technologies.

## Graphical abstract

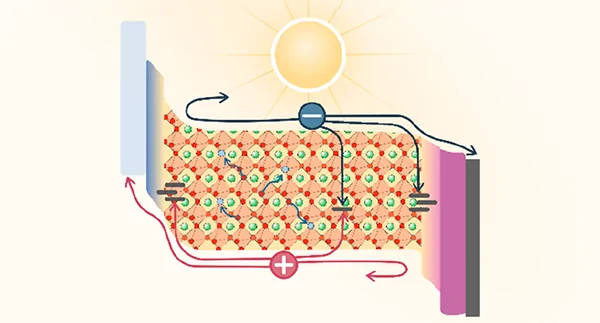

## 1 A Brief Introduction to Metal Halide Perovskite Solar Cells: Why Interfacial Energetics Matter?

Metal halide perovskites (MHPs) are characterized by the chemical formula ABX_3_ in which A is a monovalent organic (methylammonium (MA^+^), formamidinium (FA^+^), or inorganic cation (Cs^+^), B is a divalent metal cation (Pb^2+^, Sn^2+^), and X is a halide anion (I^–^, Br^–^, Cl^–^). In the absence of structural distortion and at low temperatures, perovskites adopt a cubic structure, formed by a three-dimensional (3D) network of corner-sharing halide [BX_6_]^4–^ octahedra, with the A-site cation having a 12-fold coordination, being surrounded by 12 halide anions (Figure 1a). In the MHPs, adjusting the composition by tuning A, B, and X species allows controlling the material’s properties such as band gap, charge diffusion length, exciton binding energy, absorption onset, and stability.

**Figure 1:**
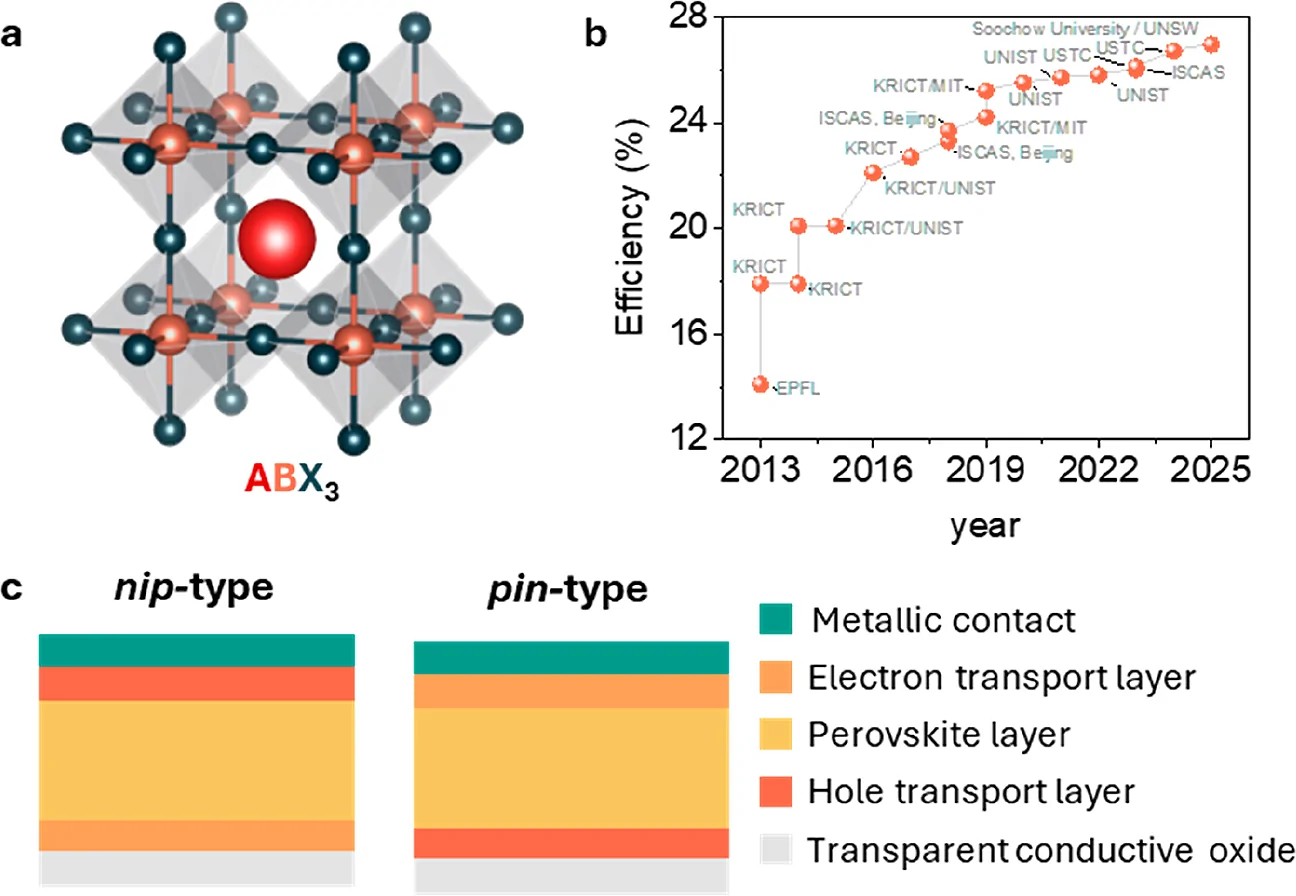
(a) Crystal structure of a cubic ABX_3_ perovskite. (b) Record power conversion efficiency of single-junction perovskite solar cells. This plot is courtesy of the National Laboratory of the Rockies, Golden, CO.^26^ (c) Schematic illustration showing the stacking in a *n-i-p*- and *p-i-n*-type perovskite solar cell.

Although the photoconductivity of MHPs was first reported in the 1950s,^1^ their popularity grew rapidly after Miyasaka and his team, in 2009, demonstrated their use as light-sensitizing materials for dye-sensitized solar cells (DSSCs).^2^ Since then, MHPs have been explored in a wide range of optoelectronic technologies, including solar cells,^3–5^ lasers,^6–8^ light-emitting diodes (LEDs),^9–11^ photodetectors,^12,13^ X-ray detectors,^14,15^ transistors,^16–19^ memristors,^20,21^ spintronics,^22,23^ and thermoelectric devices.^24,25^ The power conversion efficiency (PCE) of single-junction perovskite solar cells (PSCs), excluding tandems, has risen from 10% ^4,3^ to 27%^5^ in less than 15 years (Figure 1b), and the scalability of the technology has been demonstrated to be feasible.

To achieve high performance in PSCs, several adjacent layers are required to transport the photogenerated charges from the MHP active layer to the electrodes (Figure 1c). The order in which these layers are stacked defines two different device architectures: the regular (*n-i-p*-type) or inverted (*p-i-n*-type), as shown in Figure 1c. Importantly, the stacking of these layers shapes the energetic landscape of the photovoltaic device, influencing fundamental processes such as light absorption, charge generation, transport, and extraction, which, in turn, affect its performance. The imperfect nature of the device's internal interfaces influence the energetic landscape of the device. For example, the interfaces of the MHP active layer often exhibit undercoordinated ions due to incomplete formation of the metal halide octahedra, leading to a higher defect density than in the bulk of the perovskite film.^27^ Moreover, lattice mismatch between the perovskite and the charge extraction layers inevitably induces strain, charge accumulation, and recombination.^28,29^ Additionally, interfacial chemical processes, such as the migration of halides from the perovskite into the charge selective layers (CSLs) or metallic contacts, can progressively alter the interface composition and energetics.^30,31^ Furthermore, energy level misalignment between the perovskite and the charge extraction further exacerbates recombination losses.^32–34^ As a result, interfacial recombination in PSCs is recognized as one of the leading causes for energy losses in the device.^35,36^ In this sense, although MHPs exhibit exceptional intrinsic optoelectronic properties, the complexity of the device architecture ultimately limits their efficiency.

Overcoming these limitations requires careful engineering of the interfaces to minimize energy losses, thereby further enhancing photovoltaic performance. One effective strategy is to modulate the interfacial properties to suppress nonradiative recombination. This can be achieved through doping, dipole formation, or surface reconstruction. By creating favorable band bending at the interfaces,^37–39^ or by changing the semiconductor character via targeted doping strategies to better match the charge-selective layer,^40,41^ the driving force for charge extraction can be optimized, thereby facilitating more efficient carrier transfer from the MHPs to the charge-selective contacts. Understanding and controlling these interfacial processes is therefore critical to unlock the full potential of MHP-based optoelectronic devices. In this review, we provide an overview of the fundamental principles, experimental insights, and theoretical advances that have shaped our current understanding of interface energetics in MHP solar cells. We highlight the open questions in the field and discuss emerging concepts that may help address key challenges in this technology.

## 2 Understanding the Energy Level Structure of Metal Halide Perovskites

To understand the interfacial energetics of perovskite solar cells, the fundamental energy-level structure of the MHP active layer is crucial. In this section, a brief introduction to the energetic structure of perovskites, their defect tolerance, and ionic nature is outlined. These concepts describe the unique nature of these semiconductors, which, in turn, influence the energetic alignment of the perovskite with the other layers of the device.

### 2.1 Energetic Band Structure of Metal Halide Perovskites

To understand the energetic landscape of perovskite solar cells, it is essential to recognize that their band structure distinguishes them from those of classical semiconductors, such as group IV (e.g., Si and Ge) and group III–V (e.g., GaAs, InP, and Ga). Here, we focus our discussion on the perovskite compositions based on either Pb^2+^ ([Xe] 4*f* ^14^ 5*d* ^10^ 6*s* ^2^ 6*p* ^0^) or Sn^2+^ ([Kr] 4*d* ^10^ 5*s* ^2^ 5*p* ^0^) as the B-site cation, as these represent the most commonly used compositions. In addition, we discuss the impact of the X-site halide and the indirect influence of the A-site cation on the energetics and band structure of MHPs.

In MHPs, the orbital hybridization between the B- and X-site species gives rise to the conduction and valence bands (Figure 2a,b). More specifically, the conduction band minimum (CBM) is formed by the B-*p*/X-*s* antibonding state, with a small contribution from the X-site halide, while the valence band maximum (VBM) originates from an antibonding combination between B-*s*/X-*p* states.^42,43^ The orbital interaction gives rise to a direct bandgap at the R-point of the Brillouin zone (Figure 2c). Note that the orbitals from the A-site cation do not contribute to the formation of the electronic band structure of the perovskite. In the following sections, we revisit the discussion of the A-site cation, illustrating its indirect impact on the band structure.

**Figure 2:**
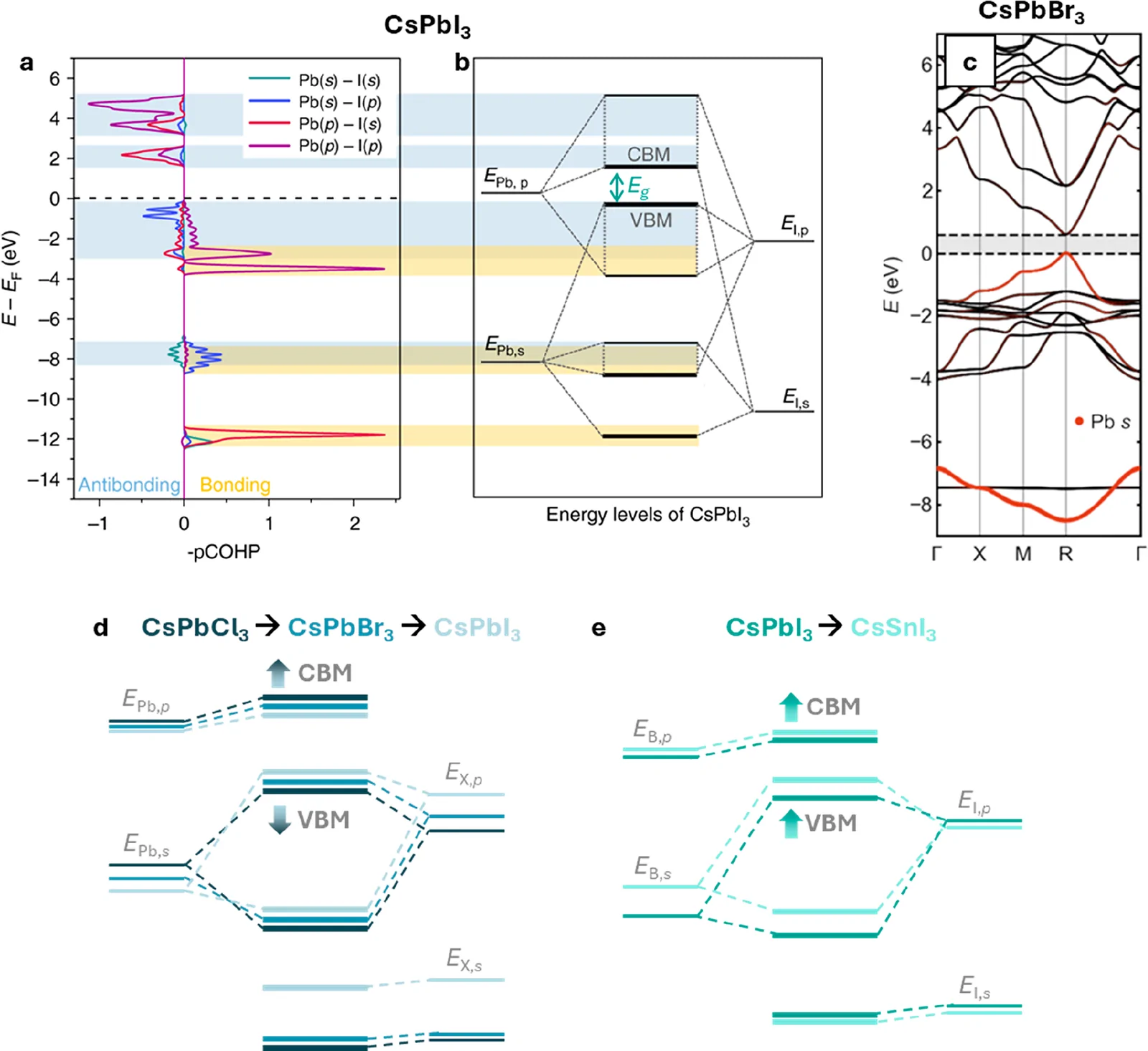
(a) Orbital-resolved Crystal Orbital Hamiltonian Population (COHP) analysis for CsPbI_3_. The positive and negative values represent the bonding and antibonding characters, respectively. (b) Schematic energy-level diagram. The colored bars guide the eye in identifying the orbital interactions that form the valence and conduction bands. (c) Electronic band structure of cubic CsPbBr_3_ perovskite. The contribution of the *s* states is shown in red. Schematic energy-level diagrams illustrating the trends resulting from changes in (d) the halide and (e) the B-site cation. Panels (a), (b), (d), and (e) Reproduced with permission from reference 43. Copyright 2019 Springer Nature. Creative Commons Attribution 4.0 International License. (c) Reproduced with permission from reference 44. Copyright 2020 Materials Research Society. Creative Commons Attribution 4.0 International License.

An important aspect regarding the band structure of MHPs is the influence of the lone electron pair in Pb^2+^ 6*s* ^2^ (or Sn^2+^ 5*s* ^2^) orbital. The interaction between B-*s*/X-*p* states to form the valence band results in a pair of bands: a low-energy one with bonding character and a high-energy one with antibonding character (Figure 2b).^44^ The presence of this lone electron pair raises the energy of the VBM compared to other semiconductors and creates a high curvature in this band, which favors a low effective mass for holes.^44^ These electrons in the central B atom are stereochemically active, meaning that they can distort the octahedra and break inversion symmetry depending on the B-site cation used.^45,46^ The stereochemical expression of the lone electron pairs depends on the relativistic contraction of the *ns* orbitals and, therefore, on the B-site cation. For instance, Pb^2+^ has a stronger relativistic effect and spin–orbit coupling (SOC) compared to Sn^2+^ due to a larger atomic number. This causes the outermost electrons in the 6*s* ^2^ orbital of Pb^2+^ to experience stronger contraction compared to the electrons in the 5*s* ^2^ orbital of Sn^2+^, meaning that the 5*s* ^2^ electrons in Sn^2+^ are more stereochemically expressed. Perovskites based on Ge^2+^ as the B-site present an even higher activity of the lone electron pair, as there is lower orbital contraction of Ge^2+^ than of Sn^2+^ or Pb^2+^.^47^ In fact, the stereochemical expression of the lone electron pair in Ge-based MHPs has been attributed to the octahedral tilting that causes instability in these materials, which includes the off-centering motion of Ge^2+^ and a phase transition at low temperatures.^48^

Since the VB and CB are derived from orbital interaction between the B- and X-site ions, it is evident that utilizing (or alloying) different species will result in changes to the band structure of the perovskite. For example, as we descend in the halogen group from Cl to I, the energy of the *np* orbitals increases in the order Cl (3*p*) < Br (4*p*) < I (5*p*). This increase in orbital energy raises the VBM and shifts the CBM downward, thereby reducing the bandgap.^49^ This behavior occurs because the VBM is primarily influenced by antibonding interactions between the B *ns* and X *np* orbitals, which means it follows the energy levels of the halide. In contrast, the CBM is derived from B *np* states and is only indirectly affected by changes in the halide. However, the electronegativity is not the only factor contributing to variations in the energy of the CBM and VBM. As the halide is varied from Cl to Br to I, the B–X bond length increases due to the larger ionic radius of the halide. The increased bond length reduces the overlap between the B-site metal and halide orbitals, weakening the antibonding character of the B–X interaction that defines the conduction band. As a result, the conduction band minimum shifts to lower energy.^43^ The reduced orbital hybridization down the group is also due to the X-*p* orbitals becoming more diffuse and higher in energy, which in turn diminishes overlap with the B-*s* orbital.^43^ Altogether, these effects cause a downward shift in the CBM from Cl → Br → I (Figure 2d). Conversely, the VBM shifts upward from Cl → Br → I due to a combined effect of the increasing halide *np* orbital energy, the longer B–X bond length, and the decreased orbital hybridization (Figure 2d). Therefore, the bandgap decreases as we go down the halogen group.

Shifts in the energy of the CBM and VBM also occur when the B-site cation is altered. For example, when replacing Pb^2+^ with Sn^2+^, the energies of both band edges shift upward (Figure 2e).^43^ This occurs because the Sn^2+^ 5*s* orbital is at a higher energy level than the Pb^2+^ 6*s*, as the Sn^2+^ 5*s* ^2^ orbitals are less stabilized, leading to a smaller energy gap difference between the 5*s* and 5*p*. Although both CBM and VBM increase in energy when Pb^2+^ is replaced by Sn^2+^, the shift is more significant on the VBM, due to the direct involvement of the 5*s* ^2^ orbitals in its formation. As a consequence, the band gap of the material decreases. Interestingly, in mixed Sn–Pb compositions, the band gap is found to be smaller than that of either pure Pb- or pure Sn-based perovskite.^50,51^ This trend is due to the electrons in the 5*s* orbitals of Sn being in higher energy than the 6*s* electrons in Pb, causing the VBM to be formed predominantly from Sn-derived states, while the CBM originates primarily from Pb-derived states in the mixed composition.^50^

As discussed above, the band edges of MHPs do not originate from the A-site cation states. This cation primarily stabilizes the lattice through electrostatic interactions and hydrogen-bonding (H-bonding) with the halides.^52^ In the case of ammonium-based organic cations, bonding energy in the order of a few tenths of eV is observed.^53^ Although the molecular orbitals from the A-cation contribute only to deep states in the conduction and valence bands,^54^ it affects the structural parameters of the perovskite, inducing strain and octahedral tilting, which is phenomenologically described by the tolerance factor (*t*, eq 1). In this equation, *r*_*A*_ *, r*_*B*_, and *r*_*X*_ are the ionic radius of A, B, and X species. In practical terms, the size of the A-site cation results in octahedral distortions that alter the crystal symmetry of MHPs. In the presence of such distortions, the bond length between the A and X species is altered, which changes the orbital overlap and, as a consequence, the bandgap.

1 $$t=\frac{r_{A}+r_{X}}{\sqrt{2}\left(r_{B}+r_{X}\right)}$$

When the size of the A-site cation surpasses a given limit established by the Goldschmidt tolerance factor (eq 1), the 3D MHP structure becomes less stable (Figure 3a). In such cases, the perovskite may form either low-dimensional structures when bulky ammonium-based organic cations (yellow region) are used, or nonperovskite phases with small inorganic cations (orange region). Within the stability window (marked in green in Figure 3a), octahedral distortions arise when *t* deviates from 1.0, resulting in lower-symmetry phases.^55^ The extent of tilting and distortion is quantified by the B–X bond length distortion index and the deviation of the X–B–X angles from the ideal 180°, which is expected in cubic perovskites.^55,56^ This can reduce B–X orbital overlap, which increases the bandgap. The influence of the A-site cation on the APbI_3_ perovskites is illustrated in Figure 3b. Formamidinium yields the most symmetric structure (*t* ≈ 1), with a bandgap of 1.59 eV.^43^ Replacing FA^+^ with a smaller cation like Cs^+^ decreases *t*, enhances octahedral tilting, reduces B–X orbital overlap, and widens the band gap. Conversely, introducing larger A-site cations, such as guanidinium, tends to expand the lattice, distorting the B–X bonds. However, when considering the influence of the A-cation, the change in the bandgap depends on the balance between bond length and bond angle distortions. It is interesting to note that the influence of the A-cation can vary depending on the B-cation.^56^ For example, in FA_1–*x*_Cs_*x*_PbI_3_, increasing Cs^+^ content widens the bandgap due to octahedral tilting and reduced orbital overlap (Figure 3c). In contrast, in FA_1–*x*_Cs_*x*_SnI_3_, Cs^+^ incorporation only causes a decrease in the cubic lattice parameter, i.e., causes unit cell shrinkage, leading to a decrease in the bandgap (Figure 3c).

**Figure 3:**
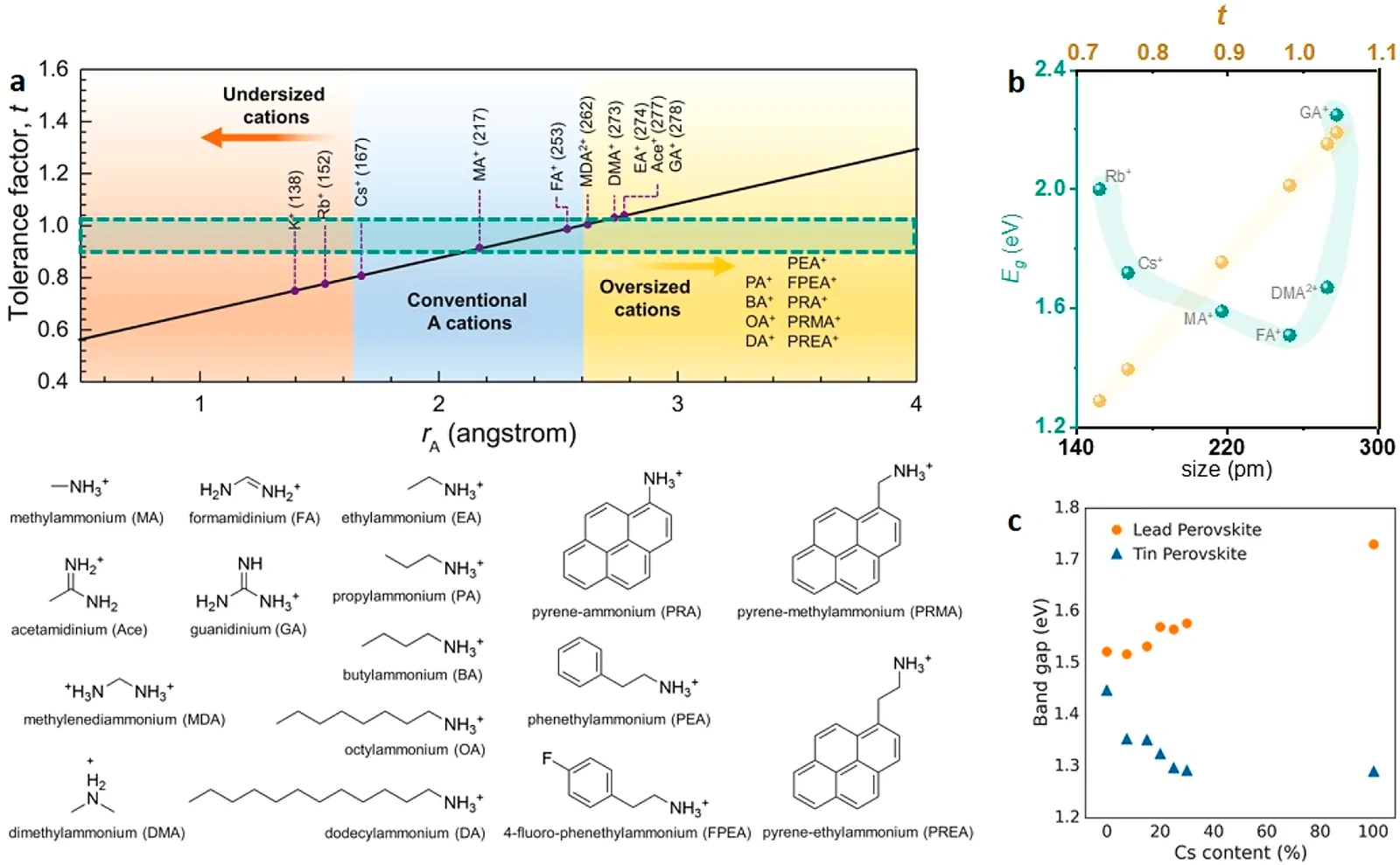
(a) Variation of the tolerance factor (*t*) as a function of the cationic radius (*r*_*A*_) for different A-site cations in an APbI_3_ perovskite, along with the molecular structure of the A-cations. The values in parentheses indicate the ionic radius of the cations (in pm). The region marked in green represents A-site cations that result in a stable 3D MHP structure, defined by 0.85 < *t* < 1.05. (b) Change in the bandgap (*E*_*g*_) and tolerance factor (*t*) as a function of the A-site cation size for APbI_3_ perovskites. The molecular size data were obtained from ref 54. The following sources were used for the bandgap values: Rb^+^: ref 57; Cs^+^, MA^+^, and FA^+^: ref 43; DMA^2+^: ref 58; GA^+^: ref 59. (a) Reproduced with permission from ref 54. Copyright 2022 American Association for the Advancement of Science. (c) Reproduced with permission from ref 56. Copyright 2017 American Chemical Society.

### 2.2 Impact of Defects on the Energetics of Metal Halide Perovskites

One of the most intriguing aspects of MHPs is their ability to tolerate defects. This means that perovskites, particularly those based on iodide and bromide,^60^ tend not to have significant changes in their optoelectronic properties when defects are present in the crystal lattice. In a crystal lattice, defects can be classified into intrinsic point defects, structural defects, and interfacial defects (Figure 4a,b). The point defects are related to missing or misplaced atoms, including vacancies (e.g., *V*_*A*_ *, V*_*B*_ *, V*_*X*_), antisites (e.g., *B*_*X*_ *, X*_*B*_ *, B*_*A*_ *, A*_*B*_ *, A*_*X*_ *, X*_*A*_), and interstitials (e.g., *A*_*i*_ *, B*_*i*_ *, X*_*i*_). Structural defects are imperfections in materials that can include atomic plane dislocations, stacking faults, grain boundaries, and large voids. Interfacial defects occur at surfaces and are often caused by incomplete formation of the metal halide octahedra. These defects may involve undercoordinated B-species, halide vacancies, and the desorption of A-cations. Our purpose here is not to provide a comprehensive analysis of defect chemistry in MHPs, but rather to understand how defects affect the band structure of the material, which ultimately impacts the energy alignment in an optoelectronic device.

**Figure 4:**
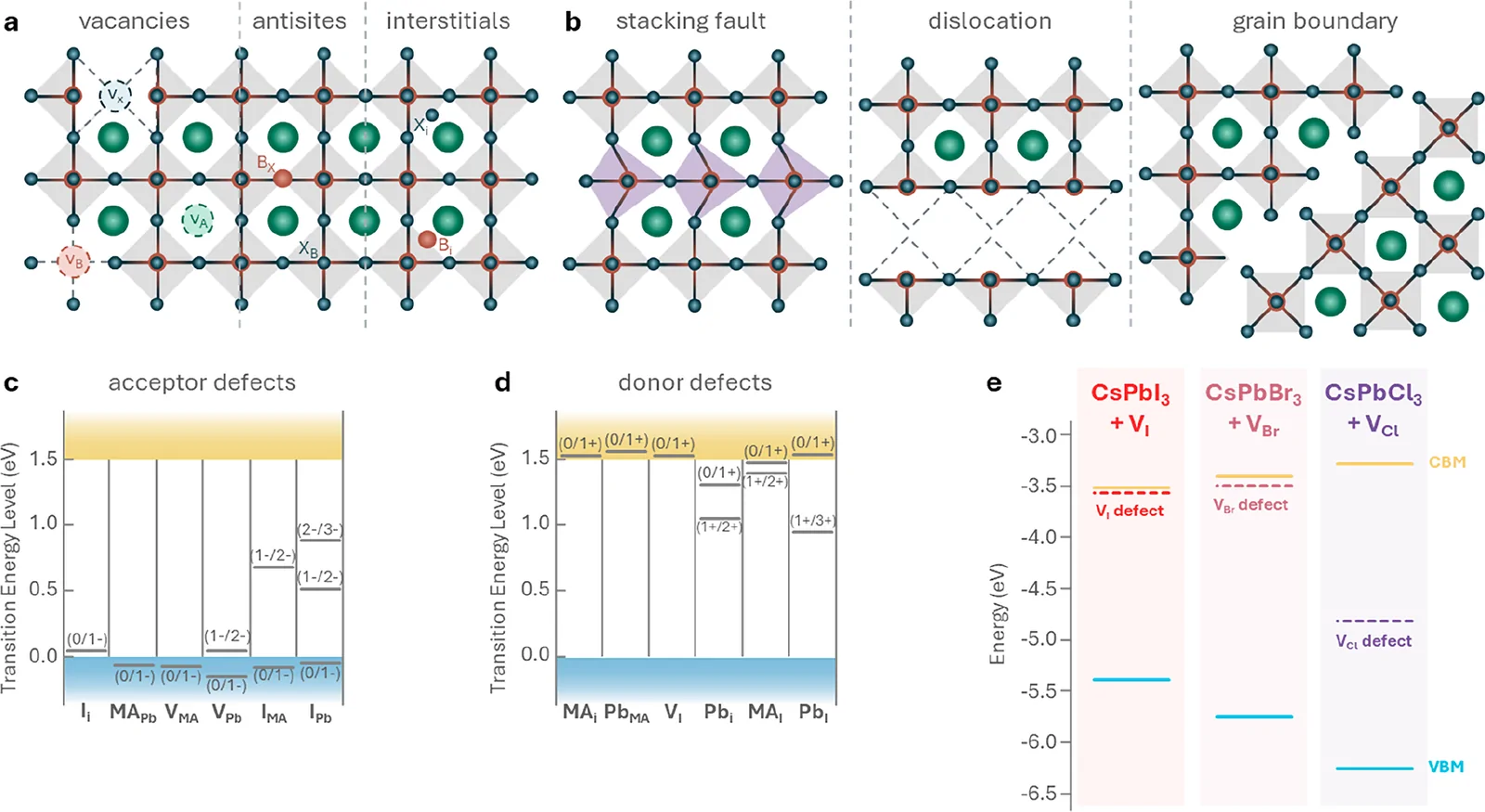
Schematic illustration showing (a) point defects and (b) structural defects in MHPs. Transition energy levels for (c) acceptor-, and (d) donor-type defects. (e) Electronic structure of CsPbI_3_, CsPbBr_3_, CsPbCl_3_, and the corresponding position of halide vacancy defect. (c,d) Adapted with permission from ref 63. Copyright 2014 AIP Publishing LLC. (e) Adapted with permission from ref 66. Copyright 2018 American Chemical Society.

Although the structural and interfacial defects can be minimized using passivation and optimization of perovskite ink formulation,^61^ point defects are always present.^62^ However, in MHPs, such as MAPbI_3_, point defects with low formation energy, i.e., those that are most likely to form, including *MA*_*i*_ *, V*_*Pb*_ *, MA*_*Pb*_ *, I*_*i*_ *, V*_*I*_, and *V*_*MA*_, form energy states close to the VBM and CBM (Figure 4c,d).^63^ Deep trap states are only observed for defects with high formation energies, including *I*_*Pb*_, *I*_*MA*_, *Pb*_*i*_, and *Pb*_*I*_ (Figure 4c,d), and are therefore less likely to be formed. For acceptor-type defects, which include *I*_*i*_ *, MA*_*Pb*_ *, V*_*MA*_ *, V*_*Pb*_ *, I*_*MA*_, and *I*_*Pb*_, their energy proximity to the VBM of MHPs is a result of the antibonding nature of this band, due to the presence of the lone electron pair on the divalent metal cation. As discussed in the previous section, the lone electron pair raises the energy of the VBM, making it in close energy proximity with the energy level from acceptor-type defects, as shown in Figure 4c. Indeed, having an antibonding state at the top of the VB is a crucial factor for a semiconductor to have shallow-energy defect states, a feature also observed for other semiconductors.^64^

On the other hand, the shallow nature of some donor-type defects (which includes *MA*_*i*_, *Pb*_*MA*_, *V*_*I*_ *, Pb*_*i*_, *MA*_*I*_, and *Pb*_*I*_) around the CBM arises from the highly ionic bonding character of the MHP (Figure 4d). It is important to mention that defects involving the A-cation, e.g., MA^+^, have a negligible effect on the electronic structure, as they do not contribute to the band structure. In other words, MA-related defects create trap states at a higher energy than the CBM; therefore, inside the CB continuum. Regarding Pb-related donor defects, they tend to form shallow states due to the weak covalent character of the CBM, which is primarily composed of delocalized Pb 6*p* orbitals.^64,65^

It is essential to note that the defect tolerance, especially regarding halide vacancies, is significantly altered when chloride replaces iodide or bromide.^60,62,66^ For I- and Br-based perovskite, halide vacancy (donor-type defect) forms a shallow energy state close to the CBM (Figure 4e).^66^ In contrast, for Cl-based perovskites, a trap state within the bandgap is observed.^66^ This can be explained by the fact that the *p* orbitals of Cl are in a much lower energy state than those of Br or I. In this sense, the resulting vacancy state will be located at a higher energy relative to both VBM and CBM, resulting in a deep trap state.^60^

Defect tolerance in MHPs has a profound impact on the charge carrier recombination, which ultimately influences the device performance. As we discussed, most low-formation energy point defects only create shallow trap states and should not significantly increase charge carrier recombination. Nevertheless, one needs to be careful when working with Cl-based perovskites. The formation of deep trap states differs from that observed for Br- and I-based perovskites. For them, even defects with low formation energy can cause trap states within the bandgap. These trap states cannot be easily populated by thermal energy and will act as recombination centers for charge carriers.

Unlike point defects, structural defects have a significant influence on charge carrier recombination in nearly all polycrystalline perovskites. Grain boundaries (GB), for instance, are known sites of increased carrier recombination rates,^67^ and several investigations have focused on passivation strategies to minimize the deleterious effect of GB on solar cell efficiency.^61^ Recently, it was found that PSC performance is significantly reduced even if the trap states derived from GB are not too deep within the bandgap.^68^ Device simulations showed that the effect of the grain boundaries on the device efficiency depended on whether the GBs hosted hole or electron traps. Hole traps at the GB are more detrimental to device performance, significantly impacting the current–voltage hysteresis (Figure 5a,b). The trapped holes remain confined near the GB, slowing their extraction rate and increasing the probability of nonradiative recombination with freely moving electrons. The effect is less pronounced when electrons are trapped at the GB, as hole extraction is more efficient in perovskites, which reduces nonradiative recombination.^68^ Experimental evidence also demonstrates charge accumulation at the GB and a consequent band bending toward, which has been claimed to help drive exciton separation, benefiting solar cell performance.^69–71^

**Figure 5:**
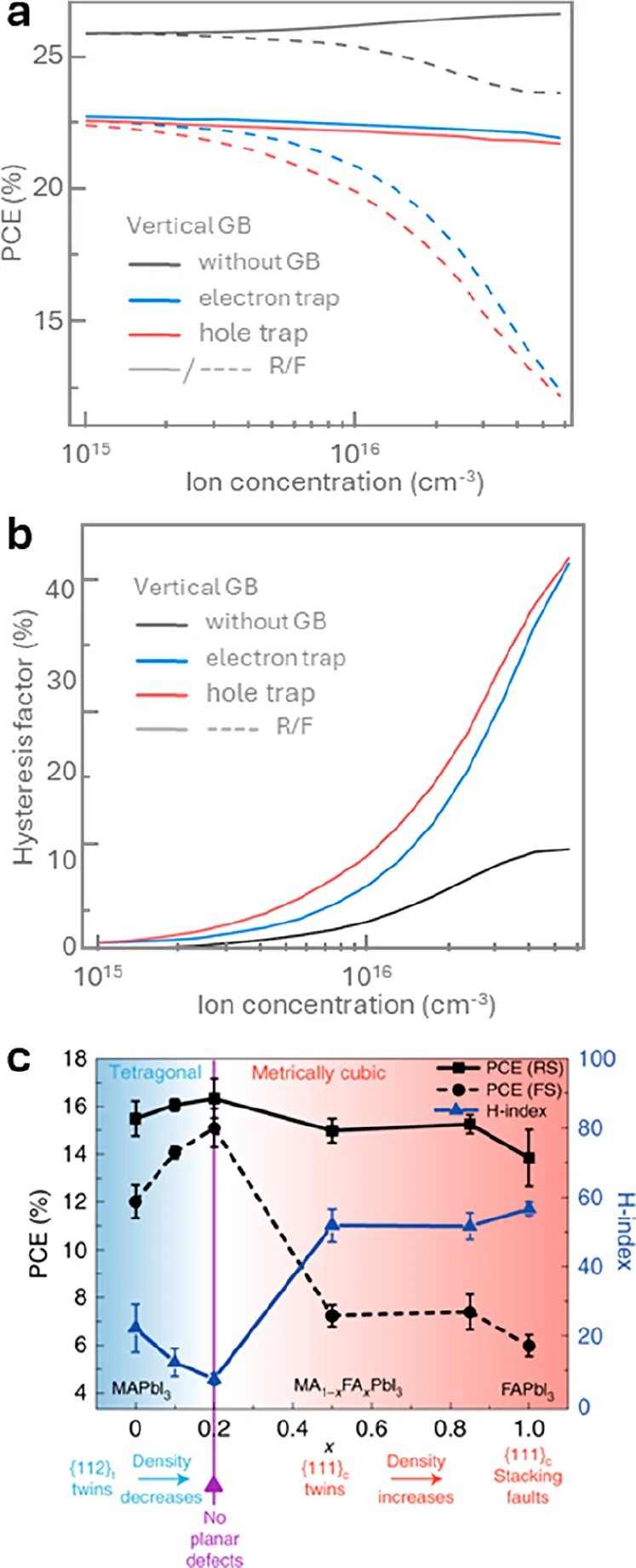
Device simulation results of the (a) power conversion efficiency (PCE) and (b) hysteresis index as a function of the ion concentration in the device. (c) Variation of the average PCE and the hysteresis index (H-index) as a function of the variation in the FA content (*x*) in MA_1–*x*_FA_*x*_PbI_3_. (a,b) Adapted with permission from ref 68. Copyright 2025 Royal Society of Chemistry. (c) Adapted with permission from ref 72. Copyright 2021 Springer Nature.

Unlike GBs, stacking faults and dislocations are less explored types of defects in MHPs, with even fewer studies linking them to PSC performance. Rothmann et al. helped shed light on these defects.^94^ Using low-dose scanning transmission electron microscopy (STEM) with advanced image processing in FAPbI_3_, the authors found that stacking faults and dislocation defects, although present in the perovskite structure, do not induce significant strain to the material. Another study suggested that stacking faults in FAPbI_3_, particularly on the (011) facet, are spots that trigger degradation in the film.^73^ Interestingly, the density of planar defects, which includes stacking faults, twins, and dislocations, can be controlled by adjusting the amount of FA in MA_1–*x*_FA_*x*_PbI_3_.^72^ Empirical observations suggested that an increase in the density of these defects negatively affects parameters such as charge carrier lifetime, open-circuit voltage loss, and, consequently, the PCE, as well as the hysteresis index (Figure 5c). It is important to note that establishing a correlation between the parameters of PSCs and charge carrier lifetime in relation to the presence of planar defects is challenging. This difficulty arises because variations in composition can also impact other aspects of the film, such as the density of point defects, doping levels, and microstructure, which may also influence the aforementioned parameters.

Another critical source of defects and, therefore, nonradiative recombination is the surfaces of the MHP film. These defects can be caused by chemical impurities, formed during the crystallization or thermal annealing steps, as well as induced by moisture and oxygen degradation.^74^ In general, surface defects typically include uncoordinated ions, dangling bonds, and nonstoichiometric surface termination, which introduces electronic states within the bandgap. Indeed, the interfaces of the perovskite have been reported as the primary source of trap-assisted recombination in the device, resulting in a significant loss of efficiency.^75,76^ Additionally, several investigations have suggested that ion migration and accumulation at the interface, in the presence of a built-in electric field,^30,31^ and nonideal stoichiometry of the perovskite, cause nonoptimal band bending at the interfaces, which deteriorates device performance.^77,78^ Beyond limiting efficiency, the high reactivity of interfacial defects accelerates chemical degradation pathways, making interfacial passivation one of the key aspects for achieving both performance and long-term stability.

## 3 Interfacial Energetics

The electronic band structure of halide perovskites offers valuable insights into their intrinsic properties, including the direct nature of the bandgap, effective carrier masses, and density of states. These factors play a crucial role in determining the fundamental optoelectronic response of the material. However, in functional devices such as perovskite solar cells, charge carriers need to be efficiently extracted across interfaces via transport layers and electrodes. When these layers are assembled into complete devices, the idealized electronic structure, typically determined by examining the individual energy level positions of the components, is inadequate for describing or predicting charge extraction. Interfaces introduce additional complexities to the system, such as energy-level misalignment, defect states, band bending, interfacial dipoles, and ion redistribution. These factors significantly shape the energetic landscape of the device.

### 3.1 Band Alignment in PSCs

Band alignment describes how the energy levels of two interfacing materials are positioned relative to each other. This provides information primarily about the internal fields across the device and, indirectly, about possible sources of nonradiative recombination. Well-described and understood types of junctions include those between metal and semiconductor, namely, ohmic or Schottky junctions. In PSCs, they can be used to describe the contact between the charge transport layer (CTL) and the top or bottom electrode (even though ITO and FTO are rather degenerated semiconductors, in most discussions, they are considered metals, for simplification). An ohmic junction occurs when the work function (*WF*) of the metal is lower than or equal to the electron affinity (*EA*) of the semiconductor (*WF*_*m*_ ≤ *EA*_*S*_) for electron contact (Figure 6a). For hole contact, the *WF* should be higher than or equal to the ionization energy (*IE*) of the semiconductor (*WF*_*m*_ ≥ *IE*_*S*_) (Figure 6b). Upon contact between these two materials, the Fermi levels align, shifting the vacuum level upward (for electron ohmic contact) or downward (for hole ohmic contact), and causing the flow of the respective carrier from the *E*_*C*_ (energy of the CBM), or *E*_*V*_ (energy of the VBM), to the metal. If the work functions of the metal and semiconductor are similar, and the semiconductor is intrinsic, then it extracts both electrons and holes.

**Figure 6:**
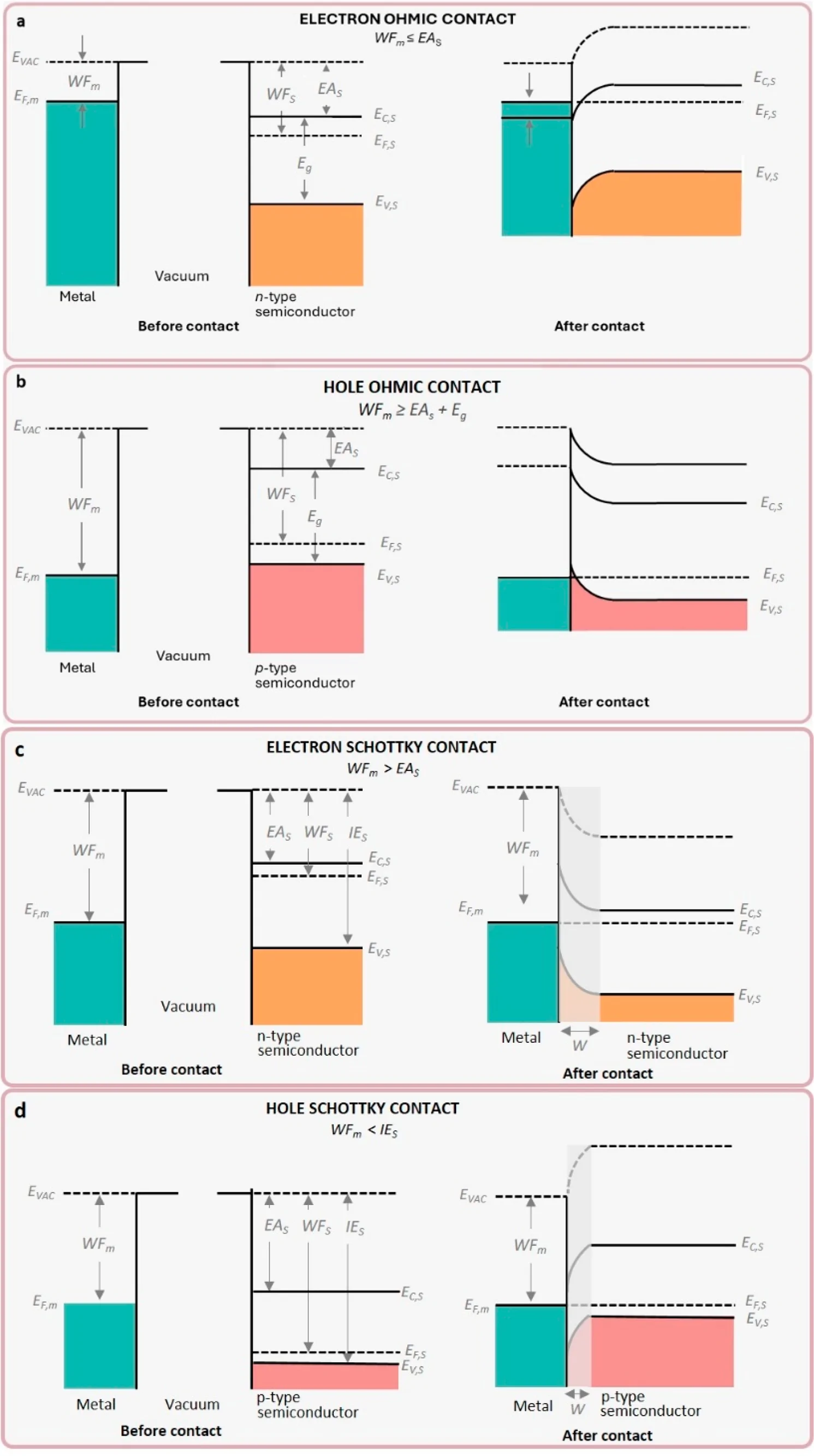
(a) Electron and (b) hole ohmic junction before and after contact. (c) Electron and (d) hole Schottky junction before and after contact.

Similarly, a Schottky junction occurs when the *WF* of the metal is higher than the *EA* of the semiconductor (*WF*_*m*_ > *EA*_*S*_) for electron contact (Figure 6c), and lower than the *IE* of the semiconductor (*WF*_*m*_ < *IE*_*S*_) for hole contact (Figure 6d). Upon contact, the energy barrier at the interface can be high enough to form a depletion region, preventing charge flow. The thickness of the depletion region, *w*, is described by eq 2, in which ϵ is the permittivity of the semiconductor, *V*_bi_ is the built-in potential, *V* is the applied voltage, *q* is the elementary charge, and *N*_*d*_ is the free-carrier concentration. As can be seen, *w* is inversely proportional to the free-carrier concentration, meaning that for very high doping concentration, the barrier can become thin enough to allow the charge tunneling.

2 $$w=\sqrt{\frac{\epsilon \left(V_{bi}-V\right)}{qN_{d}}}$$

The model that provides guidance for proposing energy band diagrams of heterojunctions between two semiconductor materials, relative to the perovskite-CTL interface, is known as Anderson’s rule, also called the electron affinity rule (Figure 7a). It predicts band alignment based on the *EA*s and *IE*s of the two semiconductors in vacuum, where the difference between their *EA* (*E*_*VAC*_ – *E*_*CBM*_) and *IE* (*E*_*VAC*_ – *E*_*VBM*_) determines the energy barriers for electron and hole extraction, respectively.^79^ However, the assumption underlying Anderson’s rule does not include consideration of the interface dipole or charge transfer. Additionally, it ignores defect chemistry and fails to account for bias or illumination conditions. The foundation of this model derives from an earlier model of Mott and Schottky, describing the metal–semiconductor junction and assuming the thermionic emission through the interface barrier, dependent on the energy difference between the *WF* of the metal and the *IE* of the *p*-type or the *EA* of the *n*-type semiconductor.^80,81^ In that case, the energy barrier corresponds to the difference in the *WF*s of the adjacent metal and doped semiconductor. Another model for predicting band alignment at a semiconductor-semiconductor junction is Tersoff’s model (Figure 7b), which accounts for interfaces rather than aligning the vacuum level. The Fermi level alignment depends on the charge neutrality level, which represents the energy level at which the density of interface states is neutral.^82,83^

**Figure 7:**
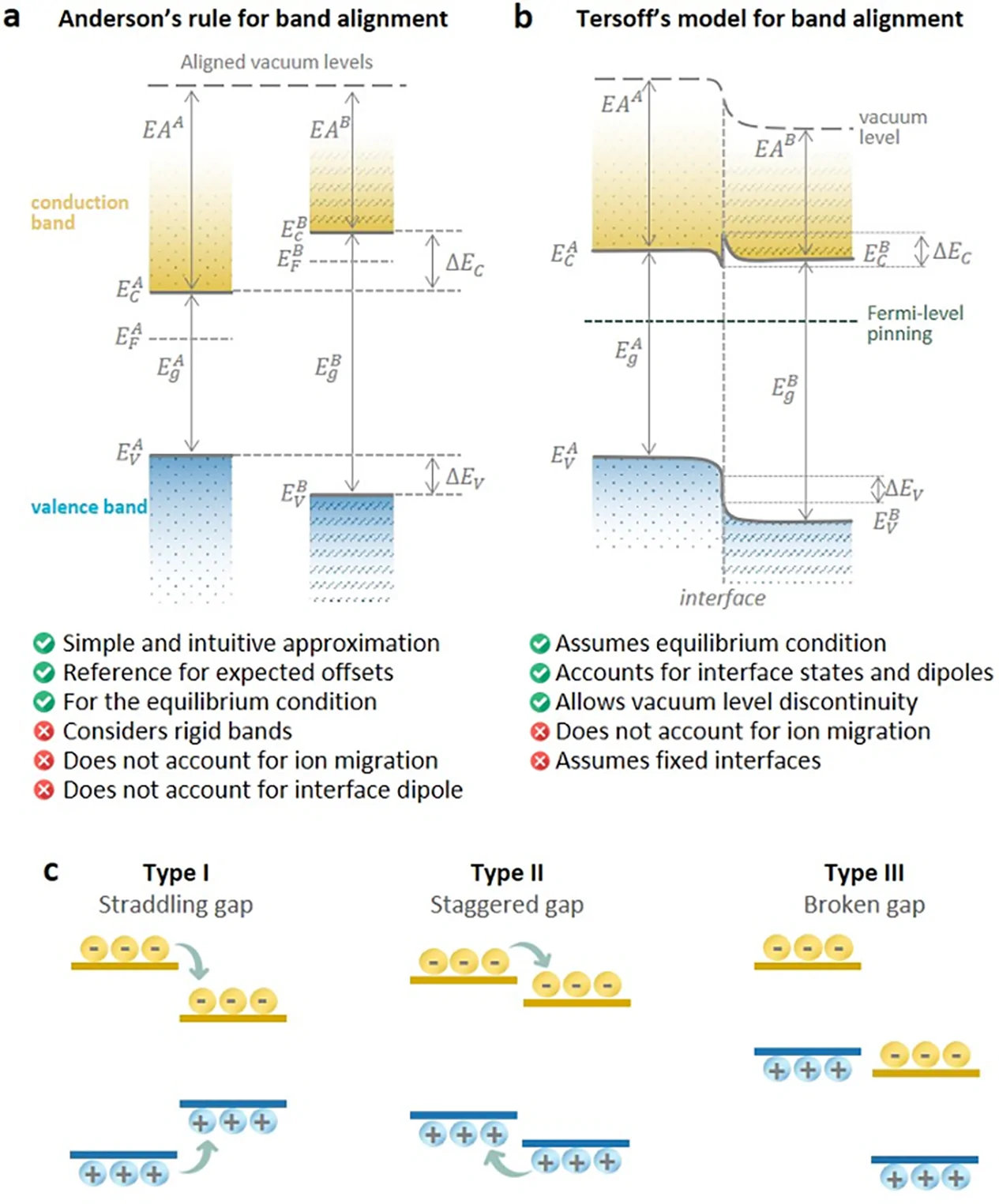
Band alignment schemes of the junction of two semiconductors A and B according to (a) Anderson’s rule and (b) Tersoff’s model. *E*_*A*_^*A*,*B*^: electron affinity; *E*_*C*_^*A*,*B*^: conduction band minimum; *E*_*V*_^*A*,*B*^: valence band maximum; *E*_*g*_^*A*,*B*^: bandgap; Δ*E*_*C*_^*A*,*B*^ and Δ*E*_*V*_^*A*,*B*^: conduction and valence band offsets between materials A and B. *E*_*F*_^*A*,*B*^: Fermi level. (c) Different types of band alignment at the junction between two semiconductors.

Over the past two decades, significant progress has been made in extending band alignment models to hybrid interfaces comprising organic and inorganic layers.^84–87^ A key advancement in this context has been the investigation of the induced density of interface states (IDIS), which provides a framework for understanding charge redistribution at complex interfaces.^88^ Earlier models that accounted for polaronic effects were later refined through a more rigorous treatment of the electrostatic potential profile in organic semiconductors, based on the solution of the Poisson equation.^87^ This led to a more general model capable of distinguishing between the idealized Schottky–Mott limit and cases where substantial charge transfer occurs during interface formation. These conceptual developments have been enabled by the emergence of advanced computational techniques for electronic structure calculations, particularly at surfaces and interfaces, as well as by improvements in experimental capabilities. Photoemission spectroscopy (PES), which will be described in detail later, allows direct, quantitative determination of band-edge positions relative to the Fermi level and of band offsets across semiconductor heterojunctions.^89^ In semiconductor heterojunctions, the interfaces can be categorized into three types (Figure 7c):

1. *Straddling (Type I)*: where both the CBM and VBM of the transport layer lie within the band gap of the perovskite. While this alignment is generally unfavorable for charge separation, it is helpful for carrier confinement. Type I alignment allows for radiative recombination in quantum wells.
2. *Staggered (Type II)*: the CBM and VBM of a transport layer should either (i) both lie below the respective band edges of the perovskite, such that the CBM falls in the gap of the perovskite (as is often the case for electron transport layers and as shown in Figure 7c), or (ii) both lie above the respective band edges of the perovskites, such that the VBM falls in the gap of the perovskite (often found for hole transport layers).
3. *Broken-gap (Type III)*: In this case, both the band edges of one material are completely above the bands of the other, which can create barriers for charge extraction and lead to recombination. When these materials are interfaced, Fermi-level equilibration leads to strong band bending and enables efficient interband tunneling. This characteristic has allowed the application of such a heterojunction in tunneling devices, such as tunneling field-effect transistors (TFET) or Esaki diodes, as well as used for charge transfer doping.^90–94^

The different types of band alignments offer distinct trade-offs in charge extraction and injection, as well as in recombination. Specifically, a slight Type II band alignment is often preferred in photovoltaic devices, as it facilitates directional charge extraction while maintaining selectivity, thereby minimizing charge carrier recombination. This principle governs the design of the two primary PSC architectures: the inverted (*p*-*i*-*n*) and regular (*n*-*i*-*p*) configurations,^95^ which are schematically represented in Figure 8.

**Figure 8:**
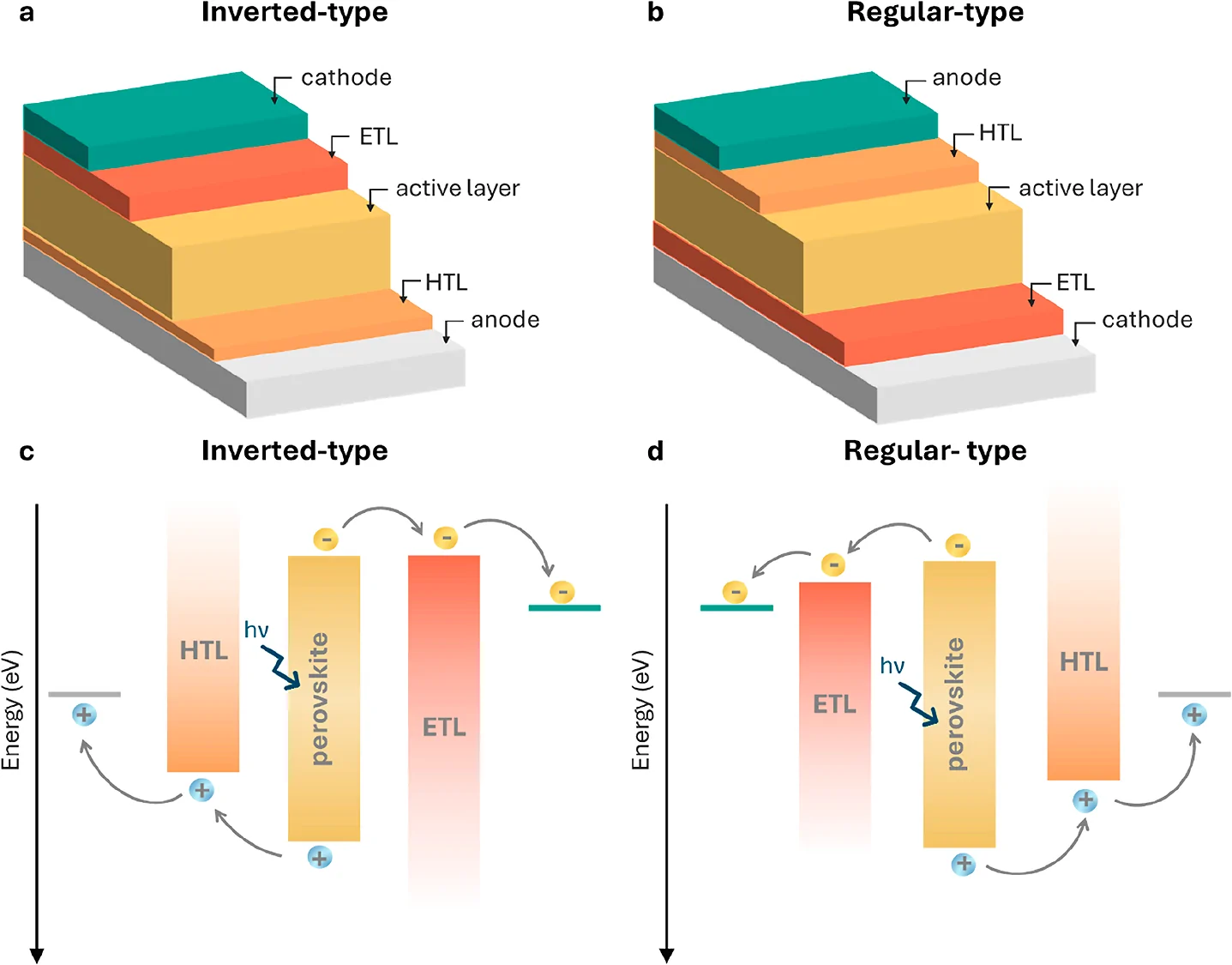
(a) Schematic diagram of perovskite solar cells in inverted (*p-i-n*) and standard (*n-i-p*) architecture. Band alignment of (c) inverted and (d) standard perovskite solar cells.

In the inverted *p*-*i*-*n* architecture (Figure 8a), the device stack consists of a transparent conductive oxide (TCO) hole-collecting electrode, a hole transport layer (HTL), the perovskite active layer, an electron transport layer (ETL), and a metal electron-collecting electrode.^96^ This configuration is well-suited for low-temperature processing due to its frequent use of organic CTLs, which makes it compatible with flexible substrates.^97,98^ The standard *n-i-p* architecture (Figure 8b) represents the historical traditional configuration for PSCs. In this design, the ETL is positioned on the TCO that acts as the electron-collecting electrode, followed by the ETL, the perovskite active layer, the HTL, and the hole-collecting electrode. While historically significant, this layout can suffer from interfacial stability issues attributed to interactions between the perovskite and common oxide ETLs, such as TiO_2_ or SnO_2_.^99,100^ Upon illumination, photogenerated electrons are extracted from the perovskite active layer to the ETL, and subsequently to the electron-collecting electrode. Simultaneously, holes are transported to the HTL and to the hole-collecting electrode. The corresponding charge-transfer of the photogenerated charge carrier from the perovskite to the charge-selective layer is shown in Figure 8d.

Although both architectures operate under the same fundamental principles, their different arrangements have a significant impact on various aspects. For example, the growth of the perovskite layer varies depending on the underlying layer. Additionally, there are optical transparency criteria for the ETL/HTL. Specifically, in the *p-i-n* architecture, the HTL must be transparent in the visible spectrum, whereas in the *n-i-p* architecture, this transparency is required of the ETL (the opposite is true in the case of the PSC used in a tandem device, where the light enters through the top electrode). Other factors influenced by the architecture include environmental stability and compatibility with different manufacturing methods.

Band diagrams of the devices offer insights into the internal fields, extraction dynamics, and barriers, which can contribute to series resistance, as well as charge accumulation, which may act as a source of shunt resistance. Figure 9a shows a band diagram of a generic perovskite solar cell in equilibrium (dark, 0 V). As the Fermi energies of all layers shifted to the same level, the difference in the vacuum levels of the two electrodes reveals the built-in potential (*V*_*bi*_) across the junction. It represents the idealized energetic landscape of the device before operation. Under illumination (Figure 9b), free charge is generated in the perovskite absorber, enabling the split of electron and hole quasi-Fermi levels (*E*_*F,n*_ and *E*_*F,p*_, respectively). The voltage builds up across the device, with a maximum of open-circuit voltage, $V_{OC}=\frac{E_{F,p}-E_{F,n}}{q}$ . At this open-circuit condition, no net charge is collected, as it all accumulates at the interfaces with the charge-transport layers due to the high energy barrier.

**Figure 9:**
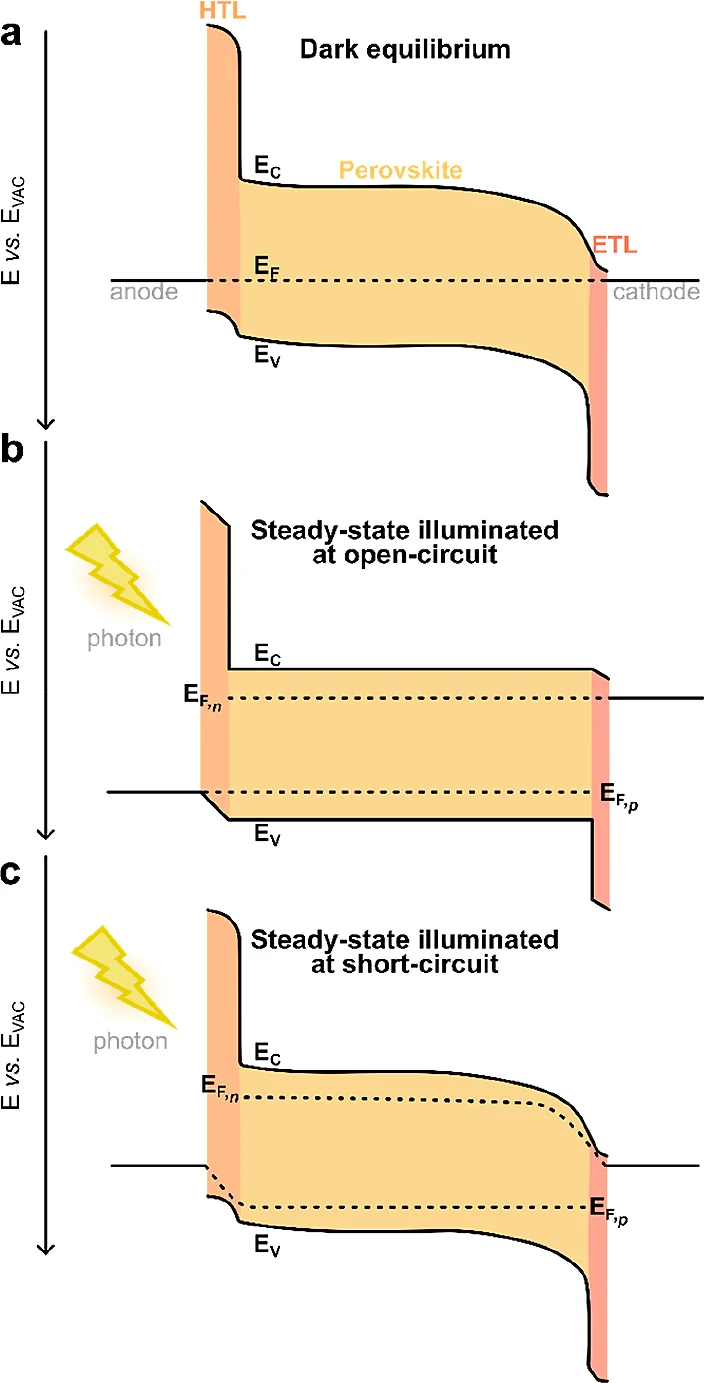
Band diagrams at (a) the dark equilibrium, (b) steady-state illuminated open-circuit, and (c) steady-state illuminated short-circuit conditions illustrating charge extraction. Adapted with permission from ref 104. Copyright 2025 American Chemical Society. Creative Commons Attribution 4.0 International. Unported License.

The most efficient charge extraction occurs at the short-circuit condition (illumination, 0 V), as shown in Figure 9c. Here, the quasi-Fermi levels are nearly flat within each contact, and the series resistance due to the extraction barriers is clearly visible. Only a few groups have presented the band diagram at the short-circuit condition.^78,101,102^ The difficulties in reaching consensus lie in the presence of mobile ions, which are known to contribute to the charge redistribution across the device, as well as can be linked to the charge extraction materials, including the potentially mobile dopants of these organic films, interface chemistry, and defects, prone to forming interface dipoles. As a result, the analytical derivation of band diagrams from the homogeneous field drop, layer thickness, and dielectric contacts almost never matches that of experimentally obtained devices. The efforts in the definition of a realistic band diagram and the relationships between the *V*_*OC*_, quasi-Fermi level splitting (QFLS), and *V*_*bi*_ include the measurements of the surface photovoltage (explained in detail in Section 4), that, performed on the complete and incomplete device stack, revealed the contribution of individual interfaces to band bending and the effective built-in potential.^103,104^

#### 3.1.1 Impact of Ion Migration

In the previous section, we discussed the ionic bonding characteristics of perovskites and their contribution to defect tolerance in the material. Although ionic point defects, such as vacancies, typically do not create deep trap states in MHPs, they trigger ion migration,^30^ which impacts halide segregation, particularly in multihalide (or multication) MHP compositions, as well as solar cell performance, stability, and current–voltage hysteresis. It is important to note that ions can migrate not only within the perovskite itself but also from adjacent layers. For example, ions such as Ag^+^, Au^+^, or Al^3+^ from the top metal electrode can move toward the perovskite layer, and Li^+^ ions from the Li­(TFSI) (TFSI: bis­(trifluoromethanesulfonyl)­imide) salt typically used to dope Spiro-OMeTAD HTL in *pin*-type PSCs can also migrate. This ionic movement in the device can profoundly affect the perovskite’s energetics, potentially leading to suboptimal band bending at the interfaces and complicating charge extraction. Additionally, chemical reactions can occur, forming insulating materials (for instance, the reaction between Ag^+^ and I^–^ can produce insulating AgI), which also deteriorates device performance and limits long-term stability.

In addition to ion migration across the electrodes and charge-selective layers, the perovskite itself also undergoes significant ion migration. This ionic movement in the perovskite layer is widely regarded as the primary cause of the hysteresis observed in the current–voltage curve. Such a phenomenon occurs because the ionic current generated during the voltage sweep contributes to the total electric current, thereby altering the electric field within the device. As a result, different device parameters are observed under forward (lower to higher voltage scan) or reverse (higher to lower voltage scan) measurements. The migration of ions in perovskite structures is facilitated by vacancies, namely *V*_*A*_ *, V*_*B*_, or *V*_*X*_ (Figure 10a). The activation energy required for ion migration varies depending on the type of ion and the composition of the perovskite. For example, in MAPbI_3_, the migration of Pb^2+^ ions is very unlikely due to its high activation energy, which was calculated by Eames et al. to be 2.5 eV.^30^ In contrast, the migration of MA^+^ ions found by the same group is 0.84 eV, whereas the lowest activation energy of 0.58 eV is found for I^–^ ions.^30^ Experimental efforts report even lower values, in the range of 0.253–0.29 eV,^105,106^ making these ions more likely to migrate, giving rise to the hysteretic behavior.^107,108^

**Figure 10:**
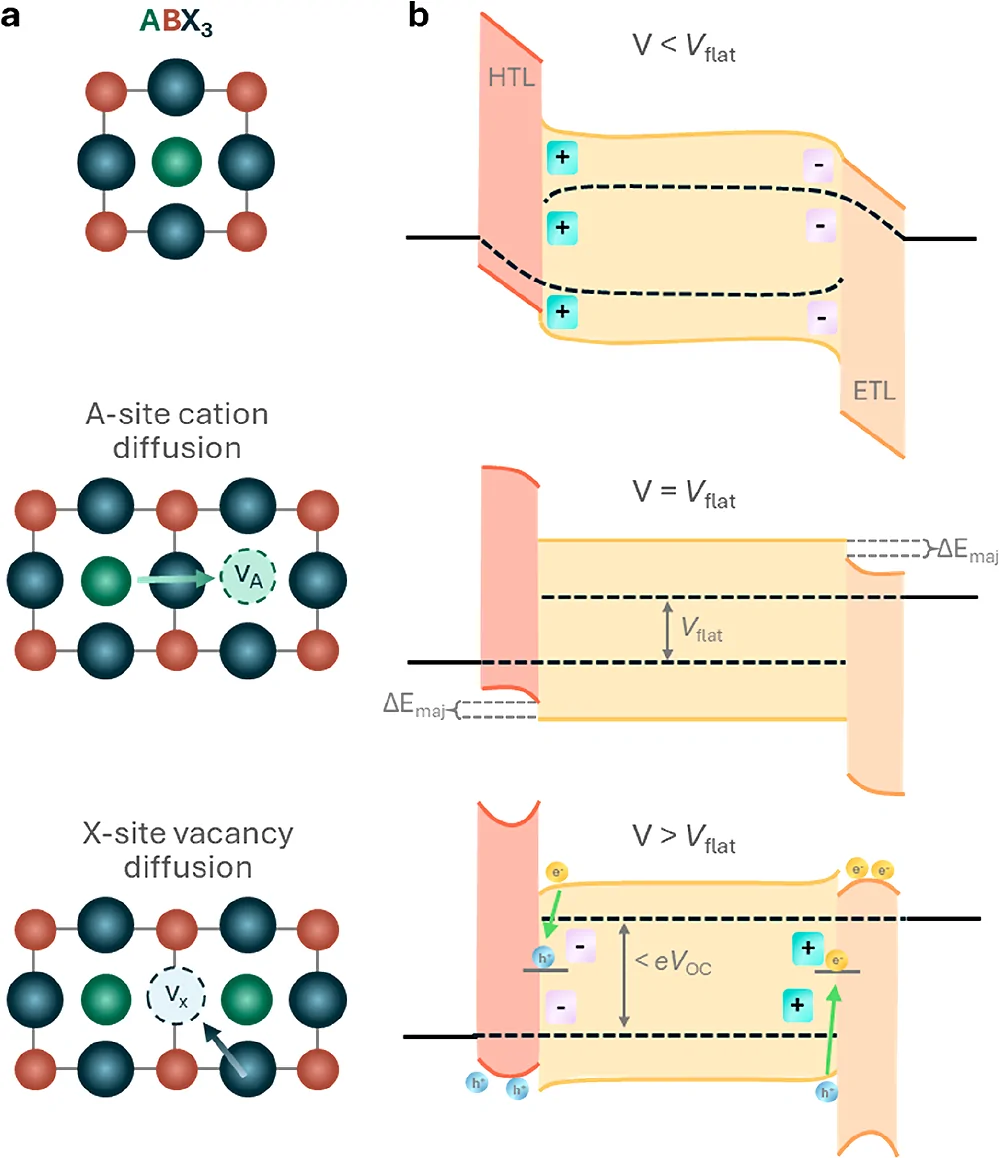
Schematic illustration of (a) the ionic transport of A- and X-site species mediated by vacancies, and (b) the impact of mobile ions on the energetics of the perovskite solar cell. Here, *V_flat_* is the flat band condition, V is the applied voltage, and Δ*E*_*maj*_ is the offset in energy between the perovskite conduction (valence) band and that of the ETL (HTL). Note that, when *V* > *V_flat_*, the e*V*_*OC*_ is maximized due to the redistribution of mobile ions. The green arrow indicates bimolecular recombination. (a) Adapted with permission from ref 109. Copyright 2018 American Chemical Society. (b) Adapted with permission from ref 110. Copyright 2024 Royal Society of Chemistry. Creative Commons Attribution 3.0 Unported License.

It is important to highlight that early interpretations of ionic migration in metal halide perovskites were strongly influenced by the highly dynamic nature of the hybrid lattice, leading to the assumption that organic A-site cations (MA^+^, FA^+^) might be responsible for the slow electrical responses and hysteresis observed in early devices. This view was supported by the perceived rotational freedom of the cations and by the similarity between perovskite hysteresis time scales and those of dipolar reorientation. Additionally, the photoluminescence (PL) measurements indicated decay times 3 orders of magnitude shorter for MA^+^ than for iodide.^111,112^ Consequently, many early studies proposed that long-range MA^+^/FA^+^ migration or dipole alignment may be a central mechanism behind bias-induced hysteresis phenomena,^113^ which is additionally supported by the decrease in ion migration upon partial substitution of the organic cations with Cs^+^.^114^ However, later reports on ion migration in fully inorganic CsPbX_3_ prompted the search for mobile ions elsewhere.^115,116^ In addition, first-principles calculations and experimental efforts demonstrated that the barriers to migrations for organic cations are far too high (≈0.8–1.2 eV) for significant transport under standard operating conditions, while halide vacancies exhibit much lower barriers (≈0.1–0.6 eV) and low formation energies.^30,112^ Direct evidence from halide segregation, using drift-diffusion measurements, operando microscopy, and spectroscopy, further confirmed that iodide and bromide vacancies constitute the dominant mobile species.^117^

In this sense, the contemporary consensus is that ionic migration in metal halide perovskites is governed primarily by halide vacancy motion, while A-site organic cations contribute only through local dipolar reorientation rather than long-range transport.^63,118,119^ This shift in understanding has unified the interpretation of hysteresis, band bending, and long-time scale device responses across the perovskite literature. It is worth noting that the activation energy for ion migration depends on the composition of the perovskite; hence, mixed-halide and further, mixed-halide and mixed-cation perovskite alloys are characterized with a significantly suppressed ion migration compared to their single-cation/halide counterparts.^65,66,120–122^ These trends can be attributed to the smaller, more electronegative nature of bromine and chlorine compared to iodine, leading to shorter Pb–Br bonds. The Cs^+^ incorporation also has a similar effect, due to its smaller ionic size compared to MA^+^ or FA^+^. Such characteristics make the crystal lattice more compact and rigid, preventing ion migration.

Ion migration also affects the band structure of MHPs. For example, the mobile ion has been found to cause band flattening in the perovskite (Figure 10b), which tends to increase the density of charge carriers within the perovskite, thereby increasing nonradiative recombination and thus decreasing device performance. Additionally, at low applied voltages, mobile ions at the perovskite/CTL interface screen the electric field, reducing charge carrier extraction and thus decreasing *V*_*OC*_.^123^ Interestingly, the same effect has also been demonstrated to increase solar cell performance. For example, Hart et al. combined experimental and theoretical approaches to determine whether the presence of mobile halides could increase *V*_*OC*_, and therefore, device performance.^110^ They have shown that mobile ions redistribute to the opposite minority-carrier accumulation region at interfaces depending on the applied voltage (Figure 10b). This results in electrostatic passivation of interface defects, reducing recombination and enabling *V*_*OC*_ to surpass the classical limit.^124,125^ Furthermore, in conditions with a high density of mobile ions, the *V*_*OC*_ becomes less dependent on the energetic alignment between the perovskite and the CTLs, which could, in principle, allow less restrictive rules for selecting appropriate materials. Importantly, for this effect to be significant, Hart et al. pointed out that the perovskite should have a high density of mobile ions.^110^ Furthermore, the *V*_*OC*_ must be greater than the voltage at flat-band conditions (*V_flat_*), which is facilitated for devices with a higher offset between the highest occupied molecular orbital (HOMO) (or lowest unoccupied molecular orbital, LUMO) of the HTL (ETL) and the VBM (CBM) of the perovskite, as this high offset restricts the built-in voltage, lowering *V*_*flat*_. In addition, the device should operate under low injection limits to reduce bulk-dominated recombination losses.

### 3.2 Losses due to Band Misalignment

Perovskite-based devices rely on multilayer architectures in which the absorber is interfaced with several charge-selective and contact layers. The presence of multiple heterointerfaces introduces additional electronic and chemical degrees of freedom, increasing the overall complexity of the device and making precise interface engineering essential. One critical aspect of this engineering is controlling interfacial energetics, as deviations from optimal band alignment (Figure 11a) can exacerbate loss mechanisms. In this section, we explore the origins and consequences of this misalignment on the solar cell performance.

**Figure 11:**
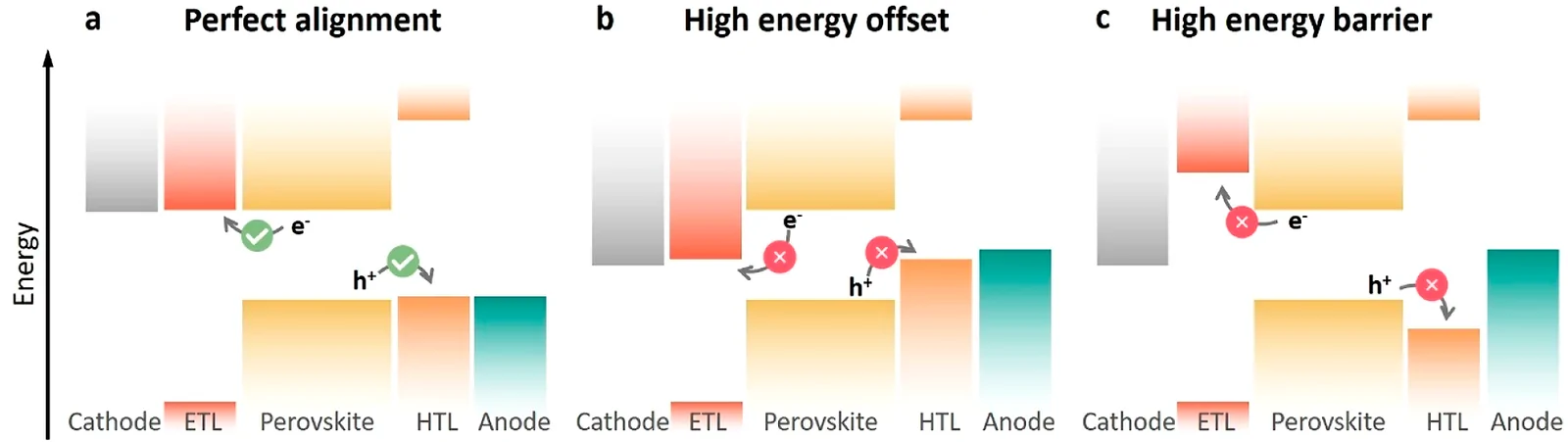
Schematic diagrams of energy level configurations in PSC. (a) Perfect energy alignment, (b) high energy offset impeding the efficient energy transfer (kinetic limitation), (c) energetically unfavorable barrier, blocking layer (themodynamic limitation).

#### 3.2.1 Energy Barriers

Energy barriers at the perovskite/transport layer interface can be broadly divided into kinetic limitations and thermodynamic barriers. Kinetic limitations arise when charge transfer is thermodynamically downhill (Figure 11b), but the significant energy offset between the perovskite and the transport layer weakens the charge transfer at this interface, thereby reducing the charge transfer rate. For instance, if the ionization energy of the HTL is significantly shallower than the perovskite valence band maximum, hole extraction remains energetically allowed, yet proceeds inefficiently due to reduced interfacial coupling. This leads to interfacial carrier accumulation and increased recombination. In contrast, the thermodynamic barrier arises when charge transfer is energetically unfavorable, requiring the charge carrier to surpass an uphill potential (Figure 11c). For example, if the electron affinity of the ETL lies above the conduction band minimum of the perovskite, electron extraction requires thermal activation or tunneling, which slows the extraction process and enhances recombination. Thus, both thermodynamically unfavorable and kinetically hindered charge transfer can limit carrier extraction, increasing series resistance and lowering the fill factor (FF).

The impact of energy barriers has been illustrated by Torres Merino et al.,^126^ where the perovskites with different band gaps and VBMs were matched with HTLs of different *IE*s. Along with an observable link between the band alignment and the solar cell performance, the most drastic change was observed when the *IE* of the HTL was 0.5 eV below the VBM of the perovskite, leading to a 50% decrease in *FF* when compared to the optimized device, which heavily impacted the PCE. In another example, Endres et al.^127^ examined the band alignment between CsPbBr_3_ or MAPbBr_3_ and the hole-transport layer of poly­[bis­(4-phenyl)­(2,4,6-trimethylphenyl)­amine (PTAA). Ultraviolet photoemission spectroscopy (UPS) and X-ray photoemission spectroscopy (XPS) measurements showed that undoped PTAA introduces a 0.2–0.5 eV barrier for hole extraction, attributed to band bending and/or an interfacial dipole. p-doping was shown to eliminate this barrier by shifting the *IE* of PTAA by ∼ 0.2 eV above that of CsPbBr_3_, facilitating hole extraction.

#### 3.2.2 Voltage Losses

In a photovoltaic device, upon illumination, photogenerated electrons and holes populate the conduction and valence bands, respectively. This process leads to the formation of distinct quasi-Fermi levels for electrons and holes (*E*_*F*__,*n*_ and *E*_*F*__,*p*_, respectively) as shown in Figure 12a. The difference between *E*_*F*__,*n*_ and *E*_*F*__,*p*_ defines the quasi-Fermi level splitting (QFLS, eq 3), which is constrained by the semiconductor’s bandgap and sets the upper limit for the *V*_*OC*_ of the solar cell. To quantify the QFLS, the photoluminescence quantum yield (PLQY) can be experimentally measured under one-sun illumination and at open-circuit conditions.^128^ The PLQY fundamentally relates the ratio of emitted and absorbed photons. Ideally, and not considering multiple exciton generation phenomena, this ratio should equal 1, indicating that all excitons undergo radiative recombination. However, imperfections in the perovskite and at its interfaces can lead to nonradiative recombination, which decreases the PLQY. This reduction, in turn, affects the QFLS and the maximum achievable *V*_*OC*_. The total loss in *V*_*OC*_ due to nonradiative recombination is expressed in eq 4, in which *k* is the Boltzmann constant, and *T* is the temperature in Kelvin.

3 $$QFLS=E_{F,n}-E_{F,p}$$

4 $$\Delta V_{OC}=-\frac{kT}{q}\ln\left(PLQY\right)$$

**Figure 12:**
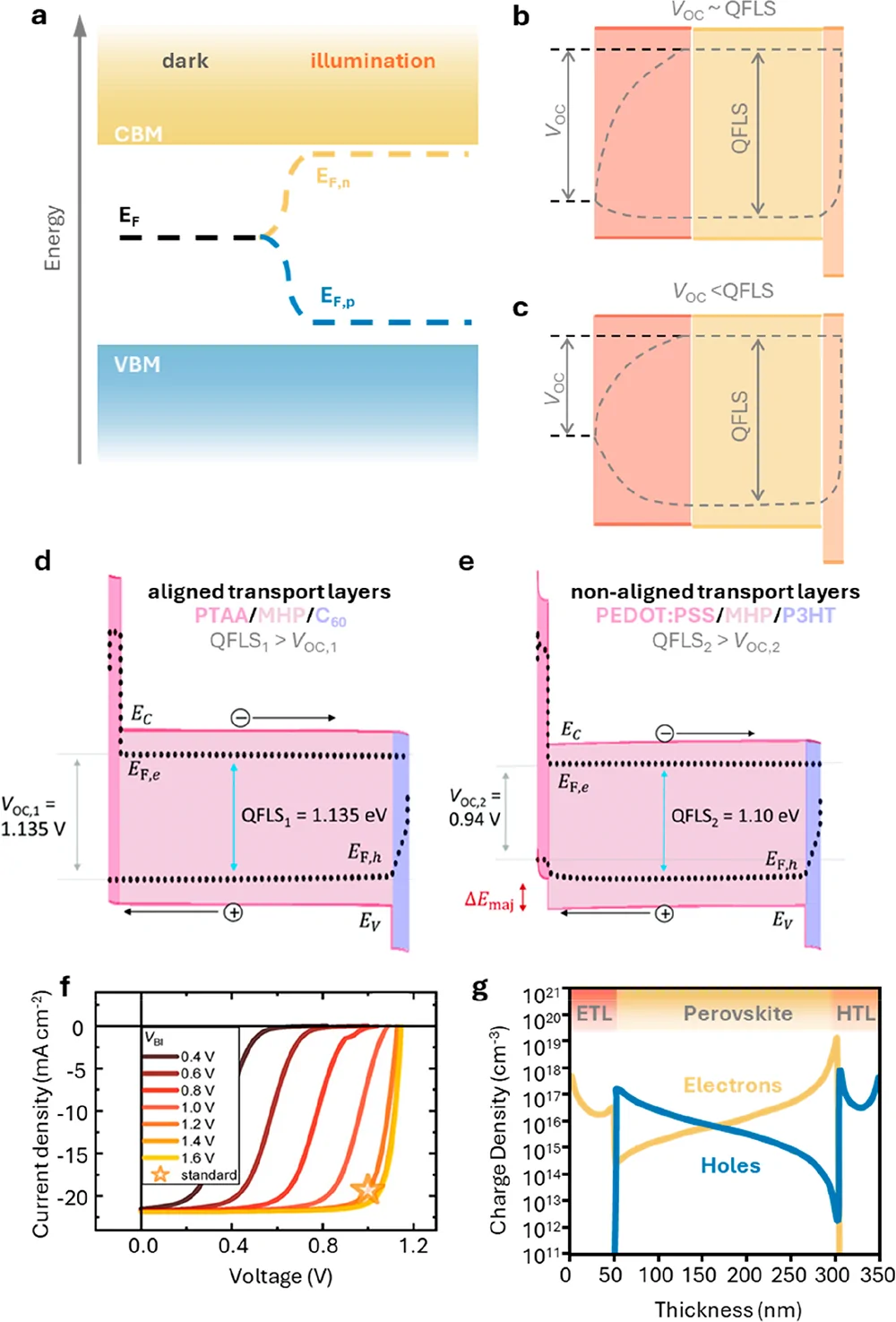
(a) Fermi level splitting into two distinct quasi-Fermi levels for electrons and holes. Schematic illustration showing the energy level alignment and the position of the electron and hole quasi-Fermi level in a device for the situation where (b) no band bending is observed, and therefore *V*_*OC*_ ∼ QFLS, and (c) band bending is observed, causing *V*_*OC*_ < QFLS. (d,e) Simulation of the energy level diagram showing the QFLS and *V*_*OC*_ in the case where the energy level of the transport layer is (d) aligned and (e) not aligned to the perovskite. (f) Dependence of the *V*_*OC*_ and FF on the *V*_*bi*_. (b,c) Adapted with permission from the ref 130. Copyright 2023 John Wiley & Sons, Inc. Creative Commons Attribution 4.0 International License. (d,e) Reproduced with permission from the ref 128. Copyright 2019 Royal Society of Chemistry. (f) Reproduced with permission from ref 118. Copyright 2025 American Chemical Society, Creative Commons Attribution 4.0 International License. (g) Reproduced with permission from ref 134. Copyright 2019 American Chemical Society.

Perovskites stand out from traditional semiconductors because they have a high *V*_*OC*_-to-*E*_*g*_ ratio. This is mainly due to their sharp band edge, low density of states near the band edges, and the antibonding nature of the valence band edge, which, as discussed, confers the material defect tolerance.^129^ These features give perovskites high charge mobility and help reduce nonradiative recombination. As a result, perovskite solar cells can achieve *V*_*OC*_ values up to 100 mV higher than silicon at similar band gaps, which can lead to a 10% relative increase in potential efficiency.^129^

In a solar cell, the junction between the perovskite and the ETL and HTL should ideally result in a continuous distribution of the *E*_*F*__,*n*_ and *E*_*F*__,*p*_ though the entire extension of the junction (Figure 12b).^130^ However, in real devices, the quasi-Fermi level bends at the interfaces, potentially leading to significant losses in *V*_*OC*_ (Figure 12c). Stolterfoht et al. explored this quasi-Fermi level bending across various combinations of perovskite, ETL, and HTL.^128^ Drift-diffusion modeling shows that when the energy levels of the CTL are aligned with those of the perovskite, the quasi-Fermi levels remain flat across the perovskite and extend to the electrodes, resulting in minimal voltage loss (Figure 12d). In contrast, when the CTL has misaligned energy levels in relation to the perovskite, the quasi-Fermi levels for electrons and holes bend near the interface (Figure 12e). This bending not only reduces the QFLS within the perovskite but also triggers charge accumulation and nonradiative recombination near the interfaces. In this sense, the authors demonstrate that minimal *V*_*OC*_ loss occurs when there is only a slight majority carrier band offset (Δ*E*_*maj*_) between the perovskite valence (conduction) band and the *IE* (*EA*) of the HTL (ETL).

Despite compelling evidence linking the energetic alignment between the perovskite and CTLs to the device’s *V*_*OC*_, the literature remains divided, with several studies reporting little or no dependence.^131,132^ For instance, Belisle et al. studied HTMs with varying ionization energies and work functions in a standard *n-i-p* PSC architecture.^131^ The experimental evidence demonstrates no changes in *V*_*OC*_ across all the HTMs investigated. The observed results can be explained by ion accumulation at the perovskite/HTL interface, which likely screens variations in HTM energetics,^131^ specific interfacial recombination triggered by the HTM,^132^ and/or change of the band alignment upon illumination (which will be discussed in more detail in Section 4.6).^133^

In summary, although it is evident that the QFLS sets the upper limit for the attainable *V*_*OC*,_ there is still no consensus on the extent to which the energy levels of the charge transport layers contribute to this limit. While some studies interpret shifts in *V*_*OC*_ as a direct consequence of HTL or ETL energetics, other reports demonstrate that the variations are statistically insignificant, suggesting that the apparent impact of CTL alignment may depend strongly on interfacial quality and device context (e.g., architecture, chemical composition of the interlayers, deposition methods, etc.). The question remains: does the band alignment impact the performance of perovskite solar cells, and if so, to what extent? In fact, determining PSC energetics is challenging, and, as a consequence, relationships between QFLS, *V*_*OC*_, and energy-level alignment are difficult to establish. The effect of the energy level of the CTLs on the *V*_*OC*_ can be obscured or averaged out by ionic contributions or defect states present at the interfaces. Note that this picture is even more complex if one includes the contribution of *V*_*bi*_. For example, Gutierrez-Partida et al., demonstrated that to translate QFLS into *V*_*OC*_, a significant *V*_*bi*_ (typically around 1 eV) needs to be present in the device (Figure 12f).^104^ The authors suggest that *V*_*bi*_ in a PSC arises mainly from asymmetry in the work functions of the electrodes.

In general, one can conclude that there is not a straightforward way to predict or explain the energetics in a PSC. The conventional energetic model, in which the *IE* of the HTL must lie above the perovskite VBM and the *EA* of the ETL must lie below the perovskite CBM, which is often successful for selecting CTLs in organic solar cells, may not apply to perovskites. However, numerical simulations can offer a more detailed insight into the physical origins of the experimentally observed trends. It was found that PSCs can indeed, to a certain extent, recover from interfacial band misalignment.^134^ For instance, due to the high density of holes in the HTL, electrons start accumulating at the perovskite/HTL interface, resulting in the formation of an interfacial dipole (Figure 12g),^134,135^ which pins the *E*_*F,p*_ close to the valence band, thus increasing the QFLS of the device and facilitating hole transfer. Dipole formation is possible due to low recombination losses, either in the bulk or at the interface, which is achievable in high-quality perovskite films and can be a determining factor in conflicting literature reports. Consistent with these findings, numerical simulations based on the energy levels of actual transport materials demonstrate that, under high interfacial defect densities, the ionization energy of the HTL has a substantially greater influence on *V*_*OC*_. In contrast, in low-defect regimes, the impact of band alignment is comparatively diminished.^136^

#### 3.2.3 Recombination Losses

The current loss in a device is primarily due to nonradiative electron–hole recombination. This can be due to energetic misalignment between the perovskite and the CSL, or to defect states, which are often concentrated at the interfaces of the MHP film. Importantly, defect formation at the surface can be further influenced by external conditions, both during and after the fabrication.^137^ Notable examples include changes that happen after exposure to moisture^138–140^ or oxygen.^141^ This characteristic is also observed in thin-film silicon photovoltaics, where high crystalline quality of the absorber layer enables carrier lifetimes in the millisecond range, vastly exceeding the microsecond-scale diffusion time to the interface, rendering bulk recombination negligible compared to interfacial recombination.^142^ In this sense, the surface properties can vary significantly from those of the bulk, and device performance is largely dependent on its interfaces.^143^ With that in mind, we will now focus on the main current losses in the heterojunctions of semiconductor devices.

The recombination current, *J*_*S*_, of electrons at a hole-selective contact at the surface (or interface) is expressed in eq 5,

5 $$J_{S}=qSn_{0}e^{\Delta E_{F}/k_{B}T}$$

where *q* is the elementary charge, *S* the surface recombination velocity, *n*_*0*_ the equilibrium electron density at the hole contact, Δ*E*_*F*_ the quasi-Fermi level splitting, and *k*_*B*_ *T* the thermal energy. For a given Δ*E*_*F*_, *J*_*S*_ increases proportionally with both *S* and *n*_*0*_. The recombination velocity *S* is primarily governed by the density of surface defects acting as recombination centers, while *n*_*0*_ depends on the potential drop across the electrodes and is thus linked to the built-in electric field. A stronger built-in field reduces *n*_*0*_ at the hole contact and, correspondingly, lowers the equilibrium hole density *p*_*0*_ at the electron contact. Minimizing *J*_*S,*_ therefore, requires interfaces with minimal defect density and band alignment that preserves the maximum built-in field. In crystalline silicon (c-Si) solar cells, this has been achieved by passivating selective contacts that permit the transport of only one carrier type while blocking the other via asymmetry in carrier concentration and energy-level alignment.^144^

While carrier recombination intuitively reduces the *J*_*SC*_ to minimize the available carrier concentration, its effect on the *V*_*OC*_ is even more critical, as the balance between generation and recombination directly determines *V*_*OC*_. Formally, the open-circuit condition is described by eq 6.

6 $$V_{\mathrm{OC}}=\frac{nk_{B}T}{q}\ln\left(1+\frac{J_{L}}{J_{0}}\right)$$

where *J*_*L*_ is the light-generated current density, *J*_0_ the dark saturation current density, *n* the ideality factor, and *q* the elementary charge. Since *V*_*OC*_ reflects the QFLS within the perovskite absorber, any increase in recombination, whether at interfaces or within the bulk, raises *J*_0_, thereby narrowing the QFLS and lowering *V*_*OC*_. This explains why studies of band alignment and interfacial losses often focus on *V*_*OC*_, even when *J*_*SC*_ is not significantly affected; recombination at misaligned contacts can still severely limit the achievable photovoltage.

Taken together, these considerations underscore that interfacial recombination processes predominantly govern current and voltage losses in perovskite solar cells. In particular, energetic misalignment and defect-rich interfaces lead to carrier accumulation, increased surface recombination velocities, and reduced quasi-Fermi level splitting. As a result, experimental techniques capable of selectively probing interfacial charge accumulation, recombination, and extraction dynamics are essential for identifying the dominant loss pathways in operating devices.

### 3.3 Impact of the Band Alignment on the Ionic Redistribution

Band alignment at the perovskite/transport-layer interfaces plays a crucial role not only in carrier extraction but also in governing ionic dynamics, which in turn impacts device stability. In perovskite solar cells, mobile ions (for example, iodide anions and iodide vacancies) and electronic carriers are generally driven toward their respective electrodes under operating conditions. Importantly, such ion accumulation is localized within narrow interfacial space-charge regions and does not imply a uniform halide depletion across the perovskite bulk, which generally remains close to stoichiometric under typical operating conditions. However, when a valence band offset exists at the perovskite/HTL interface, for instance, in the case of a misaligned HTL’s *IE*, relative to the perovskite VBM, holes accumulate within the perovskite near the interface. This accumulation establishes a local electric field that promotes the migration of negatively charged halides toward the interface, as confirmed by transmission electron microscopy (TEM), which shows iodide enrichment at both the HTL and ETL interfaces.^126^ Such ionic redistribution is detrimental, as local halide-rich regions act as low-band gap domains that funnel charge carriers, thereby enhancing nonradiative recombination.^145^ Likewise, conduction band offsets at the perovskite/ETL interface can induce electron accumulation in the perovskite bulk, attracting cations toward the interface. The simultaneous presence of both anions and cations at misaligned interfaces indicates a complex interplay between electronic band offsets and ionic motion, in which interfacial electric fields generated by carrier accumulation drive ion migration. Importantly, it has been suggested that devices with well-aligned band edges at both contacts may exhibit reduced interfacial ion accumulation, thereby mitigating halide segregation and enhancing the long-term photostability of perovskite solar cells.^126,146,147^

### 3.4 Concluding Remarks on the Interfacial Energetics

The energetic alignment of the charge transport layers with the perovskite absorber is widely recognized as a critical factor in device design. Yet, the exact nature of the losses arising from band misalignment remains difficult to disentangle. These losses originate from a complex interplay of mechanisms, including barriers to carrier extraction, enhanced interfacial recombination, and limitations to the built-in potential, all of which may act simultaneously. Numerical simulations have proven valuable for providing insight into these processes; however, they necessarily rely on assumed parameters, which can lead to oversimplification or inaccuracies given the large number of variables involved and the possibility of unknown loss channels. Experimental efforts to isolate the contribution of band alignment likewise face challenges, as surface and bulk effects are often intertwined and difficult to separate. Taken together, while interpretations must be made with caution, the consensus of the literature suggests that careful band alignment is generally beneficial for minimizing losses and improving device performance.

Additionally, the chemical and structural quality of these interfaces greatly influences device performance and stability. Defects such as trap states, surface roughness, or poor crystallinity can cause recombination, decrease carrier lifetimes, and exacerbate degradation issues, including ion migration and moisture ingress.^148^ Moreover, interfacial properties determine how effectively the perovskite integrates with the underlying or overlying layers during fabrication. Since perovskites are sensitive to processing conditions, variations in surface energy, wettability, or lattice structure can hinder the formation of a uniform film, leading to defects.^149^ Therefore, optimizing the chemistry, morphology, and electronic properties of interfaces is essential not only for achieving high efficiency but also for developing scalable, reproducible, and stable PSC devices.

To improve the band alignment in the perovskite device, many strategies have been employed, including the choice of charge-transport layers tailored so that the resulting band alignment comes as close as possible to the ideal case, followed by tuning the energy levels of transport layers via doping,^150–153^ using dipole interlayers to adjust vacuum level alignment,^154–156^ or introducing passivation layers that mitigate interfacial defects.^157–159^ These interlayer-focused approaches, as well as electrical doping of MHPs, will be discussed in detail in Section 5. However, having established the principles governing band alignment, its role in performance losses, and the available strategies to control it, the next step is to examine how absolute energy levels and interfacial band structures can be determined with high accuracy, and how the device operating conditions can impact them. This is where direct measurement techniques, such as photoemission spectroscopy, complemented by surface potential difference measurements using the Kelvin probe, become indispensable for translating conceptual energy diagrams into experimentally verified representations of device energetics.

## 4 Characterization of Energetic Alignment

This section reviews experimental approaches for characterizing energy band alignment in perovskite solar cells. Among these, photoemission spectroscopy, also known as photoelectron spectroscopy and abbreviated PES, is the most widely applied and reliable technique. Complementary methods, such as contact potential difference measurements (CPD), are also employed to provide additional insight.

All these techniques are surface-sensitive, with important implications for the interpretation of the obtained data. Surface band bending is well-known and extensively investigated in inorganic semiconductors, such as GaN or ZnO.^160,161^ In metal halide perovskites, it arises from several extrinsic and intrinsic factors that modify the surface charge density and, therefore, the local electrostatic potential.^162^ Water or oxygen can adsorb at the surface, acting as effective donors^163–168^ or acceptors,^169,170^ respectively. Intrinsic defects and dangling bonds carry an electrostatic charge that induces an electric field near the surface.^162^ Finally, the atomic perovskite termination (organic vs. inorganic-terminated surfaces) results in different charges, leading to distinct built-in electric fields even in the absence of defects or adsorbed species. For example, density functional theory (DFT) calculations demonstrate that for MAPbI_3_ the difference of CBM and VBM between PbI- and MAI-terminated surfaces can reach ∼ 1 eV.^171^ The trend is confirmed by experimental findings across different perovskite formulations, where *IE* and *WF* of lead halide-terminated surface, determined using PES, are always higher than those of the AX-terminated surfaces.^172–175^ As a result, PES-derived energy-level diagrams often reflect local surface chemistry and reconstruction rather than intrinsic bulk electronic properties, underscoring the need for caution when using PES to infer band alignment in devices, particularly for polycrystalline films with heterogeneous, dynamically evolving surface terminations.

The surface changes induced by the preferential termination or contamination can be resolved by PES depth profiling by sputtering, which sequentially exposes underlying layers. However, the measurements still reflect freshly created surfaces rather than the pristine bulk. On the other hand, sputtering can mitigate artifacts introduced during film fabrication, such as surface terminations, moisture, and oxygen adsorption. The situation is further complicated by the intrinsic chemical complexity of halide perovskites, which comprise multiple atomic and molecular species, as well as by their volatility and reactivity. For these reasons, the scope of information accessible to PES must be considered alongside careful attention to methodological choices that ensure reliable and meaningful data collection.

In the presence of donor- or acceptor-type surface states, the mismatch between their Fermi level position and that of the bulk perovskite forces charges to redistribute to reach the equilibrium. For donor-type surface states, this equilibration drives electrons toward the interface, resulting in an accumulation layer beneath the surface and a corresponding downward band bending. Conversely, acceptor-type states deplete electrons from the near-surface region, inducing upward band bending. The resulting surface band bending, established in the dark, defines the initial electrostatic landscape, which can be examined using surface photovoltage (SPV) measurements.

In response to illumination, the generated electron–hole pairs dissociate in the near-surface electric field and redistribute, thereby reducing the magnitude of the band bending (Figure 13). The resulting shift in the surface electrostatic potential, known as the surface photovoltage, can even lead to band flattening under intense illumination. The sign and amplitude of the SPV depend on whether the surface is initially electron-depleted or -rich, as well as on the density and energy level of surface states and the kinetics of charge trapping and detrapping. In metal halide perovskites, SPV has been repeatedly observed across different formulations^103,176–178^ and can reach values as high as 0.8 eV. This demonstrates, for instance, that an apparent *n*-type characteristic of the perovskite, as often suggested by the PES and Kelvin probe measurement, can result entirely from the surface band bending, with the *E*_*F*_ of the examined MHP landing midgap after applying the SPV.^179^ In this sense, the *n*-type characteristics inferred from surface-sensitive techniques such as PES and KPFM may not necessarily reflect intrinsic bulk doping but can instead arise from surface band bending and charge accumulation, as evidenced by the large surface photovoltage observed under illumination.

**Figure 13:**
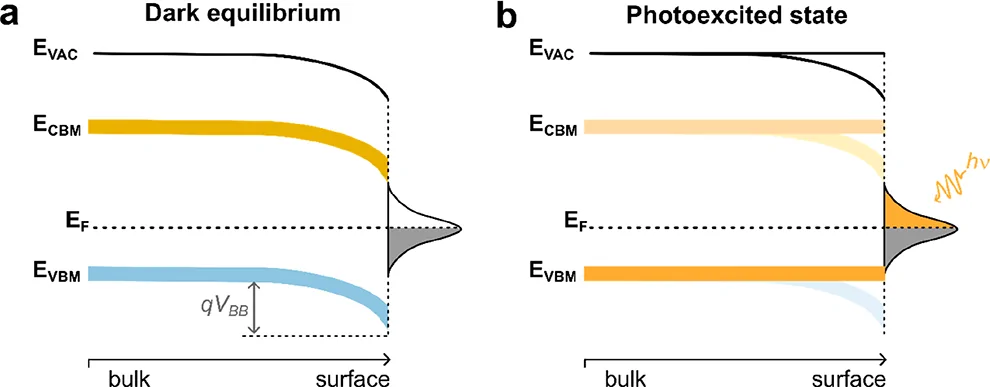
Schematic illustration of surface band bending driven by donor-like surface states, shown for (a) the equilibrium condition in the dark and (b) the photoexcited state, where illumination induces surface photovoltage and partially flattens the bands. Reproduced with permission from ref.^162^ Copyright 2022 Royal Society of Chemistry.

It is important to emphasize that the magnitude and dynamics of SPV are strongly influenced by the dielectric screening within the perovskite.^180^ Screening determines how effectively the material neutralizes internal electric fields arising from surface charges or illumination-induced charge-carrier redistribution. Typically, in the bulk, screening is provided by both the surrounding lattice and electronic polarization, while at the surface facing the vacuum directly, these polarization channels are inherently reduced, leading to less efficient screening. For example, selective probing of the surface and bulk regions of MAPbBr_3_ thin films by transient absorption spectroscopy reveals distinct carrier dynamics and spectral signatures in the two regions, i.e., surface and bulk, with an energy gradient of approximately 100 meV, with the surface exhibiting a larger effective band gap.^181^ Such an energetic offset is consistent with reduced polarization screening at the surface, where lattice relaxation and dielectric response are constrained compared to the bulk. This example helps illustrate that surface-sensitive measurements may overestimate band edge energies by millielectronvolts in metal halide perovskites, due to reduced polarization screening at the surface.

Finally, the dielectric screening in metal halide perovskites can be influenced by ionic migration, which contributes a much slower, time-dependent screening component.^182^ On short time scales relevant to UPS and XPS, only the fast channels respond, so the effective dielectric constant is lower, and changes in surface charge translate into large variations in electrostatic potential.^183^ Over longer illumination times, ionic motion further flattens the bands, reducing the apparent SPV.^184^ This hierarchy of screening processes means that the surface potential, and thus the measured energy levels, depend sensitively on the probe depth, photon flux, illumination history, and measurement time scale.^162^ Understanding these mechanisms is therefore essential for interpreting photoelectron spectra and assessing how closely surface-sensitive measurements reflect the true bulk electronic structure.

### 4.1 Photoemission Spectroscopy

Among the various PES techniques, ultraviolet photoemission spectroscopy (UPS) and inverted photoemission spectroscopy (IPES) are the most widely used to characterize the electronic structure at band edges, offering valuable insight into device operation principles. In a typical thin-film semiconductor device, charge transport involves a sequence of interfaces, such as metal–semiconductor, semiconductor-semiconductor, or semiconductor–insulator, each of which requires a precise energetic description. At the junction between these interfaces, PES techniques are valuable to directly determine the band offsets. This is important because the conduction band offset dictates electron transfer, while the valence band offset governs hole extraction.

Several key energetic parameters can be derived from such measurements, including the *WF* and *IE*. IPES provides access to unoccupied states, enabling determination of the CBM and *EA*, thereby completing the experimental description of the electronic structure. Conceptually, *IE* reflects the minimum energy required to extract a valence electron from the material surface, while *EA* represents the maximum energy released when an electron from vacuum is captured at the CBM of the material. These experimentally determined quantities serve as key input parameters for transport models. In the simplest and most commonly applied frameworks, such as the Mott–Schottky model for MHP/metal interfaces or the Anderson rule for semiconductor heterojunctions, the interface barriers are derived directly from the relative differences in *EA* and *IE* of the adjoining materials. However, these models inherently assume vacuum-level alignment, a condition rarely achieved in practice. Consequently, caution is required when interpreting PES results within such simplified schemes, and more advanced descriptions accounting for interface dipoles or an induced density of interface states should also be considered.^87,185^

While UPS and IPES are the principal tools for probing band gaps and band edge positions, other PES variants also provide valuable insights. For example, X-ray photoemission spectroscopy (XPS) enables the determination of surface elemental composition and chemical bonding environments, whereas hard X-ray photoemission spectroscopy (HAXPES) extends this capability deeper into the material, allowing access to buried interfaces.

#### 4.1.1 Working Principle

Fundamentally, PES employs the photoelectric effect. Figure 14 illustrates the three-level model of the PES process, all phenomena occurring nearly simultaneously. In the first step, the sample is irradiated with a gas-discharge lamp, X-ray tube, laser, or synchrotron source, and the incident photons (with energy *hυ*) excite electrons via the photoelectric effect. In the second step, the excited electrons are transported to the sample’s surface. Some of the excited electrons arrive elastically at the surface and retain their initial energy in the valence band; others undergo inelastic scattering, losing part of their kinetic energy as they travel to the surface, with the amount of energy lost depending on the interactions they encounter along the way. This energy loss leads to the formation of secondary electrons, which contribute to the background signal in the photoelectron spectra.^186,187^ Only electrons that acquire sufficient kinetic energy to overcome the sample’s work function can escape into the vacuum and be detected. As a consequence, a PES spectrum contains contributions from both primary photoelectrons and secondary electrons, with the secondary electrons dominating the low-kinetic-energy region near the secondary electron cutoff. However, some electrons avoid inelastic scattering, retaining their kinetic energy and escaping to the vacuum in the third step, i.e., the emission step. The emitted primary electrons exhibit well-defined kinetic energies that, to a first approximation, directly reflect the binding energies of their initial states within the solid.^188^ As a result, their spectral signatures encode information about the atomic and chemical environment of the emitting material, producing element- and compound-specific features in the energy distribution curve (EDC).

**Figure 14:**
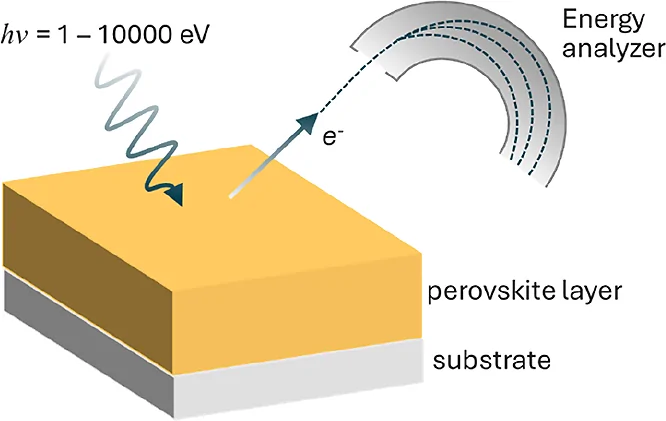
Schematic representation of the general working principle of PES.

Given this fundamental discussion, the photoemission process can be described by eq 7. Here, *E*_*kin*_ denotes the measured kinetic energy of the emitted electron, *E*_*bin*_ the initial binding energy of the electron in the solid, and *WF*_*spec*_ the work function of the spectrometer. A wide range of the electronic states can be probed, from deep core levels, through shallow core states to valence band electrons, depending on the photon energy used for the photoexcitation. The energy resolution of a photoemission experiment is influenced by several factors, including the energy spread of the photon source, the resolution of the electron energy analyzer, and the intrinsic width of the energy state from which the electron is ejected.

7 $$E_{kin}=h\nu -E_{bin}-WF_{spec}$$

An important limitation of photoelectron-based techniques is sample charging. During photoemission, the release of electrons from the surface creates photoholes, which accumulate and produce a positive electrostatic potential. This effect becomes particularly problematic in materials with poor electronic conductivity, which can cause the sample to charge, or in materials with intrinsic (photo)­chemical instabilities, as is often the case for halide perovskites. As a result, the emitted photoelectrons experience an additional energetic barrier and escape with reduced kinetic energy. In this sense, the charging artifacts manifest as erroneous shifts in photopeak positions.

In UPS, charging effects cause a peak shift toward a slightly higher binding energy due to the buildup of uncompensated positive charge on the surface.^189^ In IPES, the influx of electrons to the surface results in negative surface charging, thereby decreasing the binding energy. As a result, key parameters, such as the work function and VB onset, cannot be reliably determined. Accurate determination of band energy values demands careful sample preparation and calibration, with several procedures established in the literature,^190,191^ repeated scans to confirm spectral stability and reliability, measurements using different film thicknesses to exclude charging-induced artifacts,^189^ as well as cautious data interpretation. In an ideal scenario, the sample should be sufficiently conductive and thin enough for the charges left behind to flow out of the sample. Finally, given the limitations imposed by charging artifacts, it is crucial to cross-validate UPS results with complementary experimental techniques. Only through such corroboration can the reliability of energetic diagrams be ensured for poorly conductive or unstable materials.

#### 4.1.2 Probing Depth

The information depth in PES is fundamentally restricted by the limited escape length of photoelectrons, which in turn is governed by their inelastic mean free path (IMFP) within the solid. The IMFP represents the average distance an electron with a given kinetic energy can travel before undergoing an inelastic scattering event and losing energy to its surroundings.^192^ The function of the IMFP to electron kinetic energies, a so-called “universal curve”, shows that the IMFP reaches a minimum of about 0.5–1 nm for electron kinetic energies in the range of 50–100 eV, and increases both at lower and higher energies (Figure 15).^193^ In practice, the effective probing depth of PES is roughly three times the IMFP, with over 60% of the signal originating within the first IMFP of the surface,^192^ resulting in an accessible depth range of 1 to 15 nm, depending on the PES technique. At low electron kinetic energies, the IMFP is strongly influenced by electron–phonon scattering, which is material-dependent; accordingly, the actual probing depth can vary by up to an order of magnitude across different materials.^186,191,194^ More accurate estimates are generally obtained using the TPP-2 M semiempirical formula, as proposed by Tanuma and co-workers.^195^ This demonstrates that PES is intrinsically surface-sensitive, with the detected signal originating primarily from the outermost nanometers of the material. This characteristic is both a strength, as it enables unparalleled access to surface chemistry and electronic structure, and a limitation, since bulk properties remain largely inaccessible.

**Figure 15:**
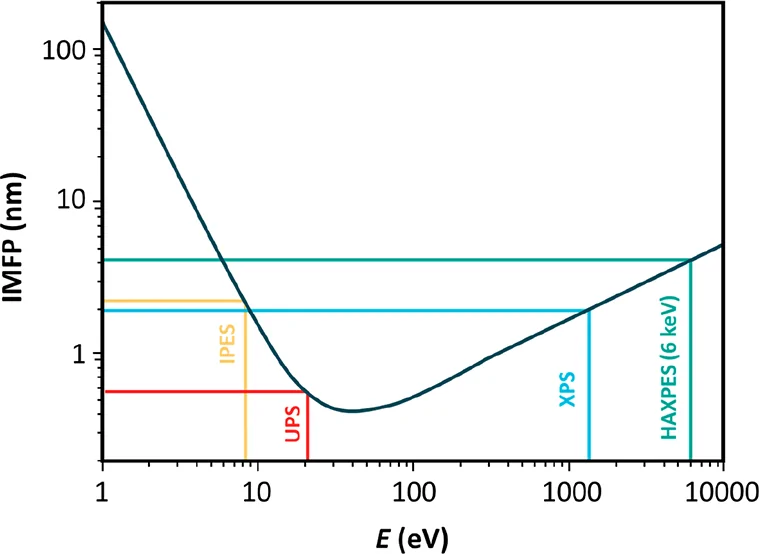
Inelastic mean free path (IMFP) of electrons in pure elements as a function of their kinetic energy, showing the “universal curve” reported by Seah and Dench. Characteristic energy ranges of different PES techniques are marked with colored lines. Adapted with permission from ref 196. Copyright 2020 John Wiley & Sons, Inc., originally based on ref 193. Copyright 1979 John Wiley & Sons, Inc.

In addition to inelastic scattering described by the IMFP, elastic processes can be accounted for by incorporating the effective attenuation length (EAL). Elastic scattering events, while not altering the electron’s energy, can modify its trajectory and angular distribution, thereby changing the effective path length before escape. As a result, the apparent probing depth may increase depending on sample geometry, electron density variations, and the detection angle.^190^

Additional depth-resolved information can also be extracted by considering energy loss features in PES spectra. Such losses may arise from core-hole excitations or from the interaction of the photoelectron with the solid on its way to the surface.^197^ Recent advances in simulation software have made it possible to account for these effects quantitatively,^198–200^ enabling studies of surface roughness and texture, as well as vertical compositional gradients in complex materials.^201^ These methodological refinements extend the reach of PES beyond simple surface analysis, providing more detailed insights into the vertical distribution of chemical species and the nanoscale structure of interfaces.

#### 4.1.3 Operating Vacuum Conditions

PES experiments are typically conducted under ultrahigh vacuum (UHV) conditions. Pressures in the range of 10^–8^ to 10^–10^ mbar are typically required to ensure reliable data acquisition. Two main reasons justify this necessity. First, UHV minimizes surface contamination of the sample. Clean surfaces are critical in PES, as even a single adsorbed monolayer can dramatically alter the measured spectral features. For instance, at a background pressure of 10^–6^ mbar, a freshly prepared surface can be covered with a complete monolayer of CO molecules within only one second, rendering surface-sensitive measurements essentially meaningless. Hence, UHV provides the temporal stability needed to probe pristine sample surfaces. Second, the low pressure ensures that the emitted photoelectrons reach the analyzer without being scattered by residual gas molecules. This is particularly important for low-energy electrons, which are highly susceptible to inelastic scattering processes that would otherwise degrade the signal-to-noise ratio and spectral resolution.^202^

The background pressure during the measurement also affects the *WF*. As the residual gas in the HV mainly comprises water vapor,^203,204^ the changes mostly carry the signature of water-induced degradation, leading to *WF* increase from 4.22 to 4.35 eV for MAPbI_3–*x*_Cl_*x*_ when the pressure changes from 10^–8^ to 10^–6^ mbar.^169^ On the other hand, many perovskites were shown to degrade upon exposure to vacuum,^205–207^ leading to the formation of impurity phases such as lead iodide and metallic lead. This highlights, once again, the caution that must be exercised in data interpretation and to comparisons across different experiments.

The requirement of UHV conditions has historically limited PES experiments to idealized laboratory environments, often far removed from the realistic operating conditions of devices. However, recent advances in vacuum technology and spectrometer design have enabled the development of near-ambient pressure PES (NAP-PES), which allows experiments to be performed at pressures up to tens of millibars.^208^ This technique utilizes a dedicated cell that encloses the sample, with the spectrometer connected via a small aperture that preserves sufficient differential pumping to maintain analyzer functionality. In this way, it becomes possible to monitor surface–gas interactions under conditions that more closely mimic those relevant to catalysis, corrosion, or device degradation. For halide perovskites, such approaches have proven useful: NAP-XPS studies can, for example, directly track the impact of adsorption and surface reactivity with gases (such as oxygen, water, or solvent vapor) on the energy levels.

#### 4.1.4 Impact of Oxygen and Water Exposure

Oxygen strongly affects the perovskite surface under ambient conditions. For instance, MAPbI_3–*x*_Cl_*x*_ samples transferred from a N_2_-filled glovebox to vacuum and subsequently exposed to 50 mbar of O_2_ exhibited a concurrent increase in work function and a shift of the *E*_*V*_ to higher binding energies.^169,170^ Unlike water, oxygen therefore shifts *E*_*F*_ toward the midgap, reducing the apparent *n*-type character of pristine surfaces.^169,170^ This behavior has been linked to the reduction of Pb^2+^ to Pb^0^ at the surface, as observed for MAPbBr_3_ films.^209^ Furthermore, oxygen can act as an electron acceptor, as upon photoexcitation it captures an electron, forming superoxide.^210^ This species initiates MAPbI_3_ degradation by deprotonating the MA^+^ cation, which subsequently desorbs from the surface, leaving PbI_2_ behind.^210^ Remarkably, as-prepared samples exposed to air showed nearly identical shifts to those observed under pure O_2_, with increased work function and an *E*_*V*_ shift to lower binding energy detectable after only 15 min.^169,211^ These results indicate that, in ambient air and on short time scales, the electronic effect of oxygen dominates that of water due to its much higher partial pressure.^169,211^

Nevertheless, water is known to play a critical role in the instability of MHPs, particularly at long time scales. For example, water can hydrate MAPbI_3_ films into its precursors, such as PbI_2_ and MAI.^212^ Probing the effects of water at different partial pressures is thus essential for understanding its influence on both structural stability and electronic properties, as well as on the interpretation of PES data.^213^ Even exposure to extremely low water pressure (10^–6^ mbar) results in a marked decrease in the work function of MAPbI_3–*x*_Cl_*x*_ films.^169^ This effect is attributed to the dipole moment of adsorbed water molecules, and the change can be reversed by mild heating or prolonged UHV storage. At higher exposure (e.g., 4 mbar of water partial pressure), water not only decreases the work function but also shifts the *E*_*V*_ onset, suggesting increased *n*-type character.^169^ XPS measurements on evaporated halide perovskite films confirmed these trends.^170^ The corresponding shift of *E*_*F*_ within the band gap has been linked to the formation of additional defect states, likely associated with an increase in metallic Pb^0^ content.^214^

Overall, these findings highlight the significant roles of both oxygen and water in altering the electronic structure of MHPs. This observation is particularly important given that MHP films are often deposited under controlled humidity conditions. Furthermore, it emphasizes the need to use encapsulation techniques across the entire film or device to prevent the migration of water and oxygen, which can lead to external degradation of the perovskite film. Additionally, it highlights the importance of established, consistent protocols for PES measurements, as samples exposed to different environments, or to the same environment but for different durations, will potentially appear to have different electronic structures.

### 4.2 Ultraviolet Photoemission Spectroscopy (UPS)

#### 4.2.1 Spectral Acquisition

UPS involves irradiating a sample with photons from the deep UV range to induce photoelectron emission, primarily from the valence band; hence, it is used to determine the work functions of metal and semiconductor surfaces. In this sense, it bridges material characterization and device function by linking interfacial energetics to operational efficiency, making it an indispensable tool in the design and optimization of high-performance PSCs. Low-kinetic-energy photoelectrons generated by ultraviolet excitation are analyzed using an electron energy analyzer, most commonly a hemispherical analyzer operated at low pass energies to maximize energy resolution. The analyzer selectively transmits electrons with kinetic energies close to the chosen pass energy, while electrons with higher or lower energies are rejected. By scanning the retarding potential applied by the electrostatic lens system, the kinetic-energy distribution of the valence-band photoelectrons is recorded, yielding spectra of electron intensity as a function of binding energy and enabling determination of the valence-band structure and work function.

To perform the UPS measurement, the sample is irradiated with ultraviolet photons, typically from a discharge lamp source of He I (21.2 eV) or He II (40.8 eV). Alternatively, laser radiation or synchrotron radiation in the far UV can be used. UPS probes the kinetic energy distribution of electrons photoemitted from the valence band into the vacuum (Figure 16a). Electrons excited during the measurement originate from across the valence band, providing a representation of the valence band structure relative to the vacuum level. This distribution reveals both the density of states near the Fermi level and the absolute energy positions of the material. The UPS spectrum extends from the Fermi level to energies above the vacuum level, capturing the full VB onset and the kinetic energy of the secondary electron cutoff (SECO).

**Figure 16:**
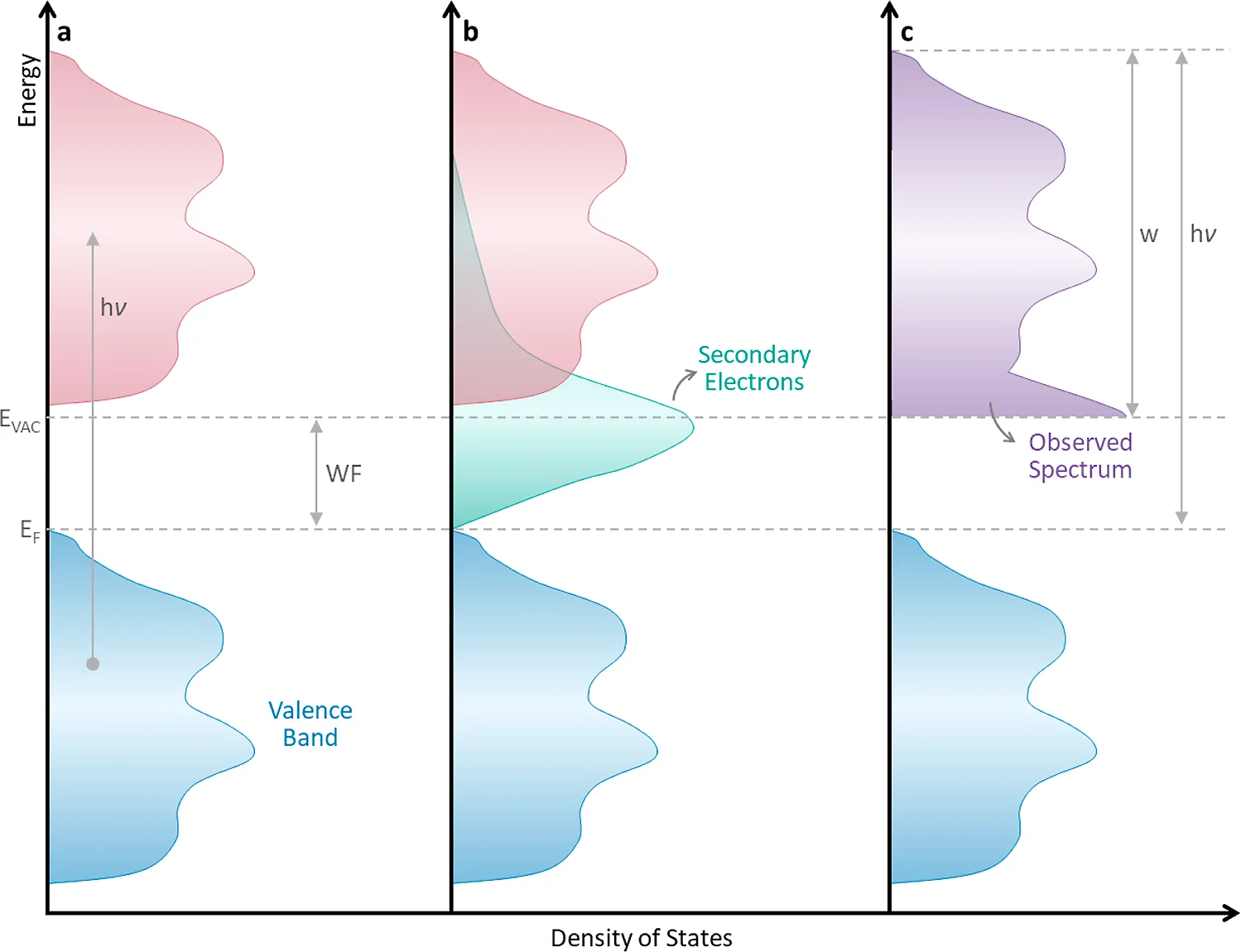
Schematic illustration of the processes leading to a UPS spectrum. (a) Deep UV photons (*h*ν) excite electrons from the valence band of the sample (metal shown) to energies above the *E_VAC_*. (b) Excited electrons travel toward the surface; some lose energy before escaping and form the secondary electron background. (c) The observed spectrum consists of elastically emitted photoelectrons and secondary electrons above *E_VAC_*. *E_F_* denotes the spectrometer Fermi level. The photon energy is the envelope of the spectral width (*w*) and the work function. Reproduced with permission from ref 189. Copyright 2023 Elsevier B.V. Creative Commons CC-BY-NC-ND license.

The Fermi level is defined by the spectrometer and the metallic sample holder to which the sample is electrically connected. It is important to note that the Fermi level of the sample itself may differ from that of the spectrometer unless proper electrical equilibrium is established between them. Assuming Fermi level alignment, the *WF* of the sample surface can be determined from eq 8.:^189^

8 WF=hν−SECO

In conventional UPS measurements using laboratory-based photoelectron spectrometers, the overall energy resolution is typically around 100 meV, although state-of-the-art instruments can be more precise. This enables detailed analysis of the VB density of states, with particular sensitivity to electronic states near the Fermi level. The achievable spatial resolution depends strongly on the photon source and electron analyzer employed, and can be reduced to approximately 10 μm in advanced setups.^215^ Under the most common experimental conditions, i.e., He I excitation at 21.22 eV, the kinetic energies of photoelectrons emitted from the valence band edge and frontier occupied orbitals typically fall in the 15–20 eV range. Because such low-energy electrons have short inelastic mean free paths, UPS is inherently surface-sensitive, probing only the outermost atomic layers. As a result, even minor surface contamination or degradation can significantly distort the observed valence band features, making careful sample handling and preparation essential for reliable characterization.

To reliably detect electrons with very low kinetic energy, which are otherwise difficult to measure due to analyzer limitations, a negative bias (*V*_*Bias*_, typically −5 to −10 V) is often applied to the sample. This potential accelerates the photoelectrons, effectively shifting the entire spectrum toward higher kinetic energies and allowing the SECO to be clearly recorded.

The inherent capability of UPS to determine *WF* and *IE* makes it a valuable tool for characterizing dipole-modified surfaces of MPHs. The “positive” or “negative” dipoles result in the increase or decrease of the *WF* of the perovskite’s surface, respectively. This has been demonstrated, for example, by using 4-CF_3_-PEAI to decrease the *WF* of the MHPs at the junction with PCBM in the inverted PSC architecture, and by using an organometallic complex based on osmium as the metal center to improve the band alignment with Spiro-OMeTAD.^154,216^

#### 4.2.2 Limitations and Pitfalls

##### 4.2.2.1 Low Density of States

One of the earliest strategies for estimating the *E*_*V*_ in semiconductors was introduced by Kraut and co-workers, who proposed extracting the onset from a linear extrapolation of the leading edge in the XPS spectra of crystalline Ge (110) and GaAs (110).^217^ Their results were consistent with density-of-states (DOS) calculations, validating the method for clean, crystalline surfaces. The same approach was later adopted for other material classes, such as conjugated organic semiconductors, where the delocalized π-system produces sharp frontier orbital onsets.^218^ However, this linear-fitting method becomes problematic for systems where the DOS near the band edges is either broadened by tail states or intrinsically low. MPHs fall into the latter category. Their reduced DOS at the band edges makes the onset difficult to resolve in conventional PES spectra, often leading to artificially deep *E*_*V*_ values, which result in overestimated band gaps. Such difficulty has also been observed for PbS-based semiconductors, which share chemical similarities with lead halide perovskites.^219^ The underlying reason for the weak DOS near the band edges in MHPs is linked to the electronic structure itself. This is because the valence band maximum originates from antibonding interactions between B 6*s* and X 5*p* orbitals, while only a small DOS contribution appears at the R-point of the Brillouin zone.^220^ This inherent characteristic underscores why standard PES methods struggle to define sharp band edges in halide perovskites.

This might cause difficulties, especially when the UV excitation is not perfectly monochromatic. Laboratory UPS light sources, which consist of discharge lamps, emit radiation from atomic transitions, resulting in a very narrow energy spread that does not limit overall energy resolution. However, depending on the discharge lamp pressure,^221^ the emitted radiation includes contributions from satellite lines, which account for a few percent of the overall intensity. These satellite lines include He Iα, He Iβ, and He Iγ at 23.09, 23.75, and 24.05 eV, respectively, and can introduce weak satellite structures in the UPS spectrum. While this is generally not problematic, it can significantly affect studies focusing on small densities of states near the valence band maximum, typical for metal halide perovskites. A proposed solution to this problem is to subtract those lines from the measured spectrum, as shown in Figure 17, where the raw CsPbI_3_/NiO_*x*_ data contain an estimated <3% satellite contribution.^221^ When plotted on a semilogarithmic scale, the original spectra show a noticeable background that obscures an accurate determination of the VBM. After satellite removal, the onset becomes much clearer, making the VBM extraction significantly more reliable. Another approach is the use of monochromatic photon sources or synchrotron facilities, which can significantly reduce satellite features and the inelastic background. This leads to cleaner spectra and enhances the accuracy of band-edge determination.

**Figure 17:**
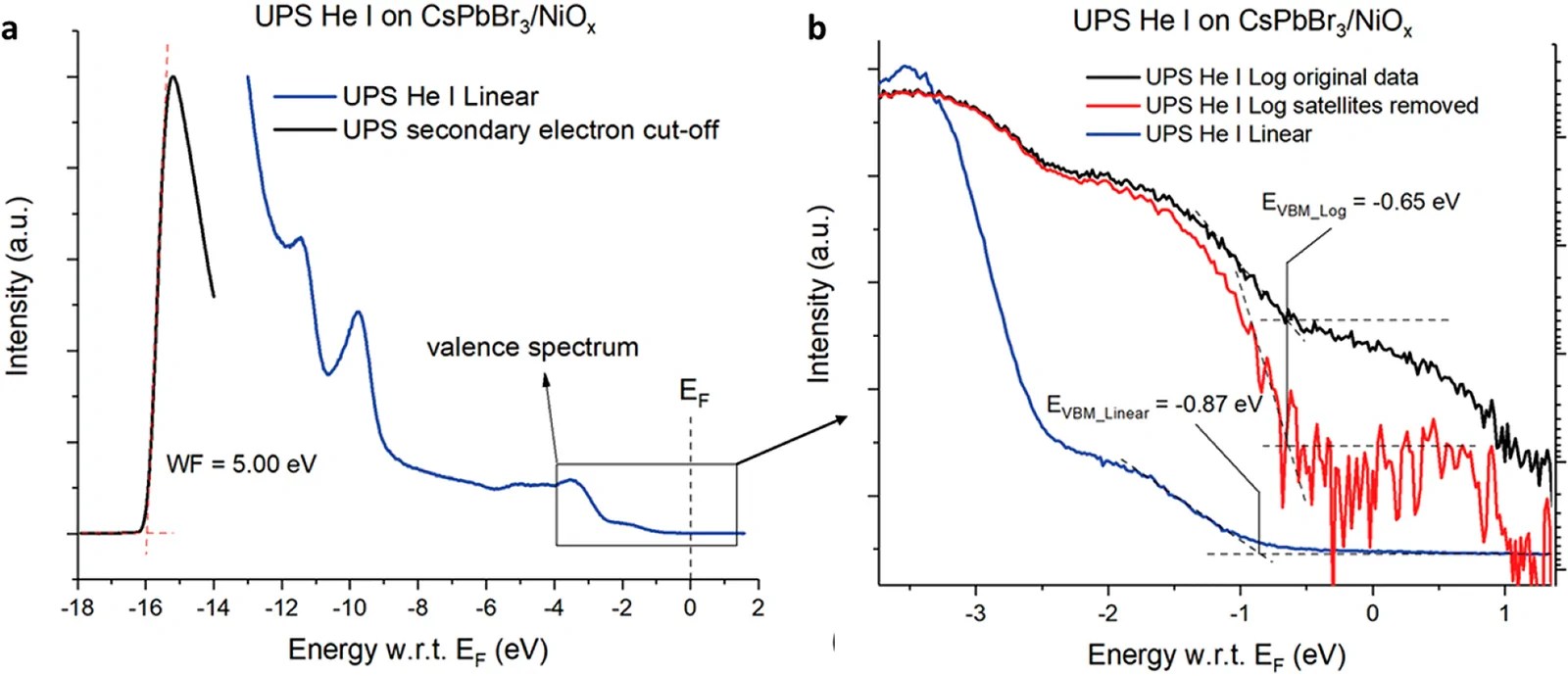
UPS He I on CsPbBr_3_ plotted in (a) linear and (b) logarithmic scale, with and without the satellites removed. Reproduced with permission from ref 221. Copyright 2020 John Wiley & Sons, Inc.

Semilogarithmic (semilog) plots are commonly used to visualize defect states, whose DOS are not apparent on a linear scale.^222,223^ However, the commonly used He I discharge lines yield a minimum measured DOS (background level) that is only 2–3 orders of magnitude below the VB signal, limiting resolution and quantitative analysis. Therefore, even though applying a semilog scale to metal halide perovskites can be technically justified, since their intrinsic DOS near the band edges can also be very low, it becomes less evident to distinguish these low-DOS intrinsic states from genuine in-gap defect states and the background noise of the instrumentation. Hence, the straightforward use of a semilog scale for UPS spectra of MHPs is debatable. In this regard, Endres et al. proposed using semilog to enhance the visibility of weak signals near the band edge. They demonstrated that this enables a good fit to the DFT findings, whereas the readout from the linear scale yielded an error of ∼ 0.3 eV for MAPbBr_3_.^224^ On the other hand, in the work of Emara et al. a good agreement with DFT was obtained using the linear representation, rather than the semilog one.^211^ Additionally, Yang and Yang proposed that the most reliable result can be obtained by plotting the spectra recorded at the *M*-point, using the angle-resolved UPS (ARUPS), in a linear scale and adding a fixed energy value, calculated with the DFT, while by using the semilog scale, the *E*_*V*_ readout depends on the excitation energy.^225^ However, this approach cannot be applied to polycrystalline thin films typically used in devices, where the measured signal is a mixture of contributions from all wavevectors.

The quantitative UPS analysis can be further exacerbated by surface contaminations. Philippe et al. demonstrated the impact of carbon-based surface contamination on the measured DOS near the valence band edge of the perovskite film.^226^ The contribution of this contamination is especially enhanced when low photon energies are used (as in standard UPS measurement), as compared with more bulk-sensitive hard X-ray photoemission spectroscopy (HAXPES, discussed in more detail in Section 4.5). Other factors could also contribute to different readouts of *IE* values, notably the density of electrically active vacancies, which can be influenced by the precursor’s ratio, sample degradation, and exposure to environmental factors, such as oxygen, water, or solvent vapors. As a result, the literature reports a wide range of *IE* values for the same perovskite composition.. Figure 18 shows that the *IE* uncertainty can reach even 1 eV for MAPbI_3_ (the most widely reported MHP) and >0.5 eV for other formulations. Obtaining a good UPS spectrum requires minimizing the exposure of the sample to possible contaminants and atmosphere, possibly limiting the acquisition time, and using a monochromatized light source.

**Figure 18:**
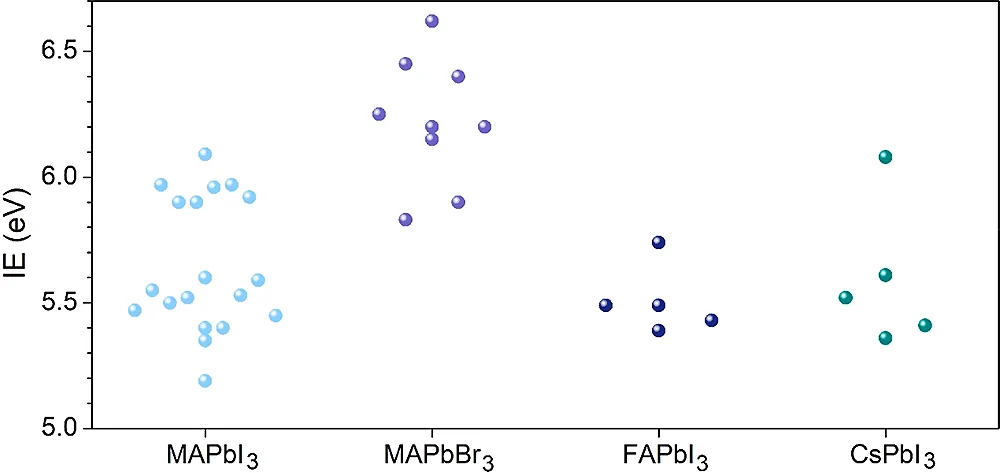
Ionization energies reported for MHP films of different formulations. The values of *IE* are reproduced from references.^222,223,235–244,227,245,246,228–234^

Faced with these inconsistencies, Menzel et al. performed a complex PES study on a triple cation Cs_0.05_(MA_0.17_FA_0.83_)_0.95_Pb­(I_0.83_Br_0.17_)_3_, comparing the traditional UV source of He I line (21.2 eV) and the near-UV source (NUPS, 6.5 eV) with the results obtained using constant final state yield spectroscopy (CFSYS).^247^ CFSYS is a photoemission technique that combines the determination of photoelectron yield with the constant final state mode of detection.^248^ In practice, the method relies on calculating the photoelectron yield, defined as the ratio of the photoelectron count rate to the absorbed photon flux, while varying the excitation photon energy. Keeping the final state of the detected photoelectrons constant removes the influence of variations in excitation intensity across the spectral range. Within the reported experiment, the signal-to-noise ratio is several orders of magnitude higher for CFSYS than for the reported UPS data, enabling a more precise detection of the DOS.

Figure 19a shows a plot of photoemission intensity for the He I discharge lamp, NUPS, and for CFSYS. The detection limit of He-UPS remains relatively high (≈10^19^ cm^–3^ eV^–1^), and its noise floor intersects the exponential tail of the perovskite DOS at an arbitrary energy. In contrast, NUPS and CFSYS employ a double-grating monochromator that improves monochromacy, eliminates satellite corrections, and lowers the detection limit by several orders of magnitude (≈10^16^ and 10^15^ cm^–3^ eV^–1^, respectively). The comparison clearly shows that many features appearing as “tail states” or defect states in He-UPS spectra may simply reflect limited signal-to-noise ratio rather than real electronic structure. When plotting these results on a semilog scale, it is evident that the exponential tail of the DOS gains disproportionate weight, and the extracted VBM position strongly depends on whether the signal lies above or within the noise (Figure 19b). Because He-UPS noise overlaps the low DOS region, it yields an artificially deep VBM (*E*_*V,log*_ ≈ −1.19 eV). In contrast, the higher signal-to-noise of NUPS (−0.81 eV) and especially CFSYS (−0.68 eV) shifts *E*_*V,log*_ progressively closer to the Fermi level. This trend indicates that the apparent energetic position of the VBM on a semilogarithmic plot is primarily determined by the detection limit of the technique and the scale used for the value extraction, rather than by intrinsic physics. When evaluated on a linear scale, which is less sensitive to the noise floor, the different methods converge within ∼ 150 meV, whereas the semilog-derived values differ by >800 meV. This illustrates that while semilogarithmic plots are helpful for visualizing low DOS, they can be misleading for quantitative VBM determination, especially when the signal-to-noise ratio is insufficient.

**Figure 19:**
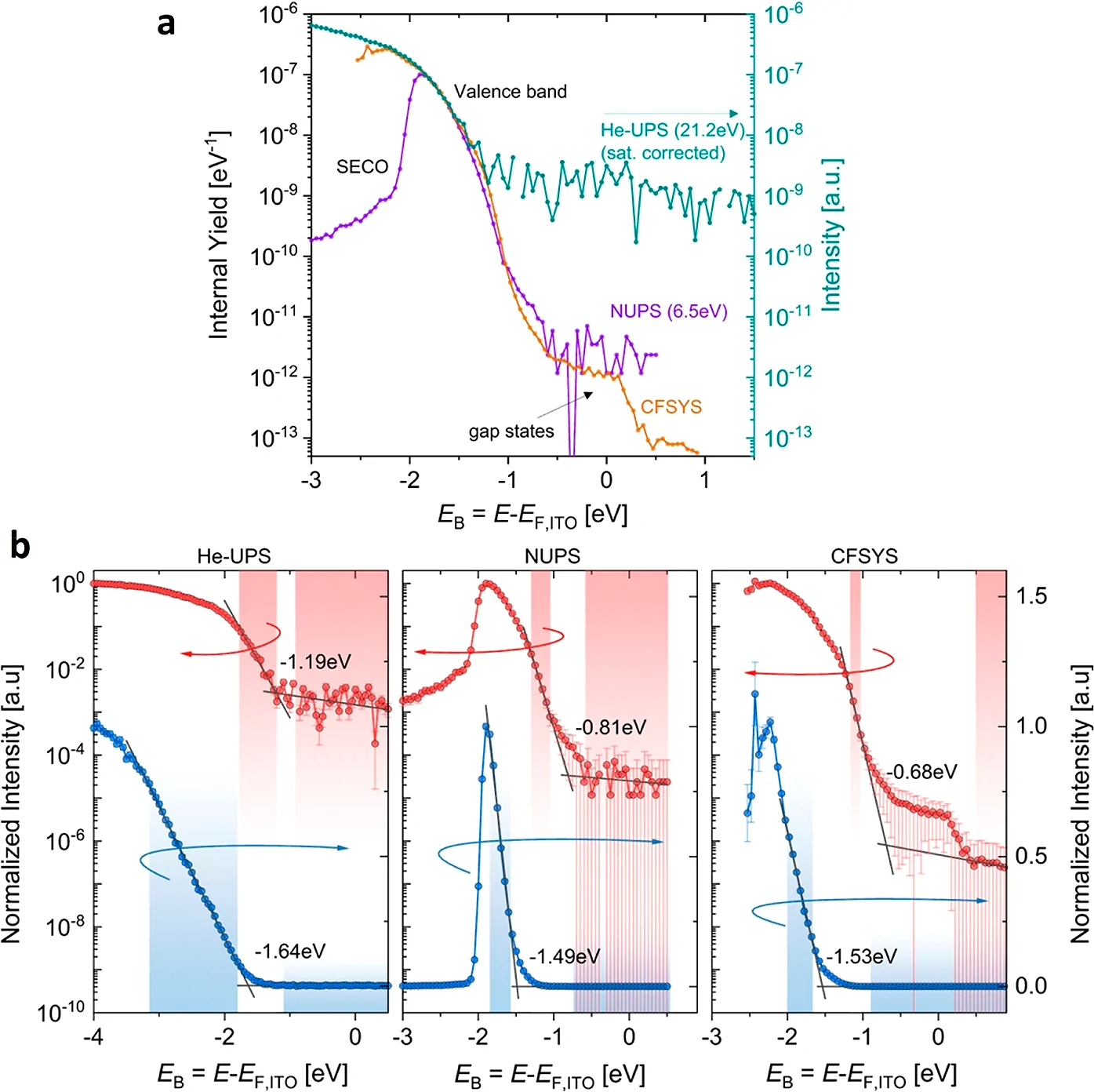
(a) The photoemission signal of Cs_0.05_(MA_0.17_FA_0.83_)_0.95_Pb­(I_0.83_Br_0.17_)_3_, obtained using two different sources (21.2 and 6.5 eV), as well as with CFSYS. For the NUPS and CFSYS spectra, the internal yield is measured, whereas the photoemission intensity of the He-UPS spectrum is scaled to fit with the other two measurements in the corresponding VB region. (b) The three spectra plotted in the linear (blue) and semilog (red) scale, with extracted values of energy with respect to the E_F_ for each measurement and each data presentation, demonstrating a discrepancy up to 0.85 eV. Reproduced with permission from.^247^ Copyright 2021 American Chemical Society.

##### 4.2.2.2 Reproducibility

Most in-laboratory protocols for preparing PSCs via wet-chemistry techniques, such as spin coating or blade coating, remain largely artisanal in nature and are therefore highly sensitive to subtle variations in processing conditions. Even vacuum-based, fully evaporated devices can suffer from batch-to-batch variability. Beyond the inherent limitations associated with the deposition procedure itself, environmental factors such as temperature, humidity, solvent atmosphere, and residual oxygen, as well as the choice of precursor materials, their purity, and the solvents used, all play a decisive role in determining film formation and interfacial quality. In this regard, Emara et al.^211^ investigated the correlation between the electronic properties of MAPbI_3_ films prepared via different methods and the efficiency of the corresponding perovskite solar cells. Using UPS, they analyzed 40 MAPbI_3_ films and found that variations in fabrication conditions resulted in a wide range of electronic properties, with *IE* values spanning approximately 0.8 eV (Figure 20). This variability was attributed to fluctuations in the Pb/N ratio. Similarly, Wang et al.^249^ reported a dependence of the work function on the deposition method, although the observed spread in *IE* values was less pronounced than in the study by Emara et al.^211^ In both cases, changes in the electronic structure were correlated with modifications of the surface stoichiometry, as determined by XPS. Fassl et al. showed that even when the same deposition method is used, unintended variations in the perovskite precursor stoichiometry may modify the *IE* by 0.4 eV.^250^ These studies highlight the utility of PES in linking surface composition to electronic properties in halide perovskites. At the same time, they demonstrate a significant sample-to-sample variability, and ambiguity in surface electronic properties can occur, even when other metrics, such as device performance, remain largely unaffected.

**Figure 20:**
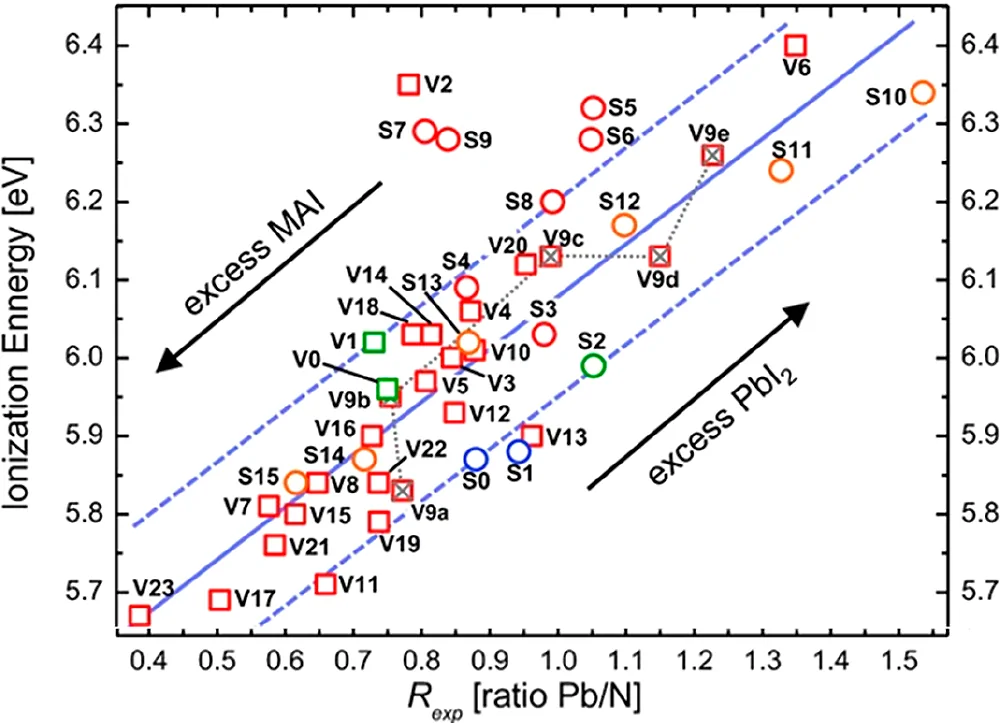
Ionization energy as a function of MAPbI_3_ stoichiometry. Different symbols represent different fabrication methods, i.e., dip coating (blue circle), 2-step solution (green circle), co-solution with additive (red circle), co-solution without additive (orange circle), 2-step evaporation (green square), and co-evaporation (red square). Reproduced with permission from ref 211. Copyright 2016 John Wiley & Sons, Inc.

##### 4.2.2.3 UV-Illumination

The characterization of perovskites by PES, as well as their operation as light-harvesting layers in photovoltaic devices, inherently involves exposure to electromagnetic radiation. Recent studies have shown that SPV of up to 0.7 eV can develop in a halide perovskite film when the UV photon flux is varied during UPS measurements under standard laboratory conditions.^176^ This photovoltage arises from the generation of electron–hole pairs upon UV excitation, which can significantly compensate for the band bending at the perovskite surface. The group of Prof. Koch reports a more pronounced, light-induced phenomenon associated with Pb^2+^ reduction and trap formation. These trap states tend to pin the Fermi level at the surface and induce downward band bending, mimicking intrinsic *n*-type behavior.^176^ Upon UV excitation, this bending is partially released by a surface photovoltage, shifting the Fermi level by several hundred meV toward the midgap. However, this effect is only partially reversible, as prolonged illumination enhances Pb^0^ formation and iodine deficiency, eventually pinning the Fermi level close to the CBM and even creating metallic aggregates at the surface. As a consequence, the electronic structure probed by UPS under illumination may differ significantly from the equilibrium one in the dark, and apparent interfacial energy offsets can reflect dynamic, light-induced charge accumulation rather than fixed band positions. This illumination-induced band realignment can substantially modify the apparent electronic structure of metal halide perovskites.^179,251–253^ Thus, without careful consideration, the UPS-derived picture of interfacial alignment may misrepresent the actual device operating conditions or even lead to misleading conclusions in device modeling.

#### 4.2.3 New Directions in the Study of MHPs by UPS and Related Methods

##### 4.2.3.1 High-Sensitivity Ultraviolet Photoemission Spectroscopy (HS-UPS)

Future developments in ultraviolet photoelectron spectroscopy are increasingly focused on enhancing sensitivity and extending the dynamic range to enable reliable detection of very low densities of gap states. Considerable progress has already been made in this direction. Sueyoshi et al. demonstrated that by dispersing the He I light source with a grating, combining it with two-dimensional photoelectron detection, and suppressing electrical noise from connectors and cables, the detection limit could be reduced to below 10^18^ states cm^–3^. This advancement, termed high-sensitivity UPS (HS-UPS), enabled meaningful analysis of photoemission data via logarithmic signal scaling.^224,240,254–257^ Building on this, Boehm et al.^258^ showed that replacing the conventional He I excitation with the narrow line width 10.2 eV hydrogen Lyman-α emission significantly improved the signal-to-noise ratio by up to 2 orders of magnitude while simultaneously mitigating radiation-induced damage to sensitive samples.

More recently, HS-UPS employing photon energies tunable below that of He I (hν-dependent HS-UPS) has emerged as a powerful approach. This technique achieves a dynamic range exceeding 6 orders of magnitude, far beyond that of regular UPS, enabling a much more accurate determination of the density and energy distribution of electronic gap states.^259^ A recent investigation using variable-energy HS-UPS has, for example, enabled the direct detection of deep-gap states in Br-based MHPs, revealing that these states are also present in MAPbBr_3_ single crystals and are not solely related to polycrystallinity, with the density suppressed upon incorporation of the 2D perovskites precursors.^257^ Moreover, the dependence of photoemission intensity on excitation energy provides additional information: it can be used to approximate the wave function of the initial state, since the intensity distribution at a constant initial energy is directly related to the Fourier transform of the electronic state.^260^ Together, these advances pave the way for UPS to not only achieve unparalleled sensitivity for probing defect-related states in metal halide perovskites and other semiconductors but also to provide deeper insights into the fundamental electronic structure beyond the valence-band edge.

##### 4.2.3.2 UPS Depth Profiling

While UPS is a surface-sensitive technique, its combination with a soft etching method, such as argon cluster sputtering, enables probing of the energetic landscape in the bulk and across buried interfaces (Figure 21a). The method, termed UPS depth profiling, was initially developed for organic photovoltaics,^261,262^ but recently was also applied to perovskite solar cells.^263^ For example, Sutanto et al. applied UPS depth profiling to study 2D/3D perovskite interfacial energy alignment in a standard *n-i-p*-type PSC. As the etching depth increases, the UPS shape changes accordingly (Figure 21b), indicating probing of different layers and enabling reconstruction of the 2D/3D interface energetics (Figure 21c).^264^ The 2D/3D heterointerface was formed in situ after applying a solution of the ammonium-based organic halide salt, thiophenemethylammonium (TMA^+^), onto the preprepared 3D perovskite. Interestingly, the authors found that the energetic alignment at the interface depends on the choice of the halide counterion used for the formation of the 2D layer (Figure 21d–f). Specifically, while Cl-based salts led to an energetic barrier for hole extraction (Figure 21i), Br and I led to the formation of a *p*–*n* junction, facilitating efficient charge extraction (Figure 21g,h). In particular, using Br-containing salts for 2D layer formation forms an effective electron-blocking layer, eliminating interfacial recombination losses at the 2D/3D perovskite interface, and leading to a *V*_*OC*_ as high as 1.19 V. This work highlights the relevance of deep-profile techniques for analyzing the energetics at semiconductor-semiconductor interfaces. It also shows that even minor alterations in the molecular structure of perovskite modifiers can lead to significant changes in the interface’s energetics.

**Figure 21:**
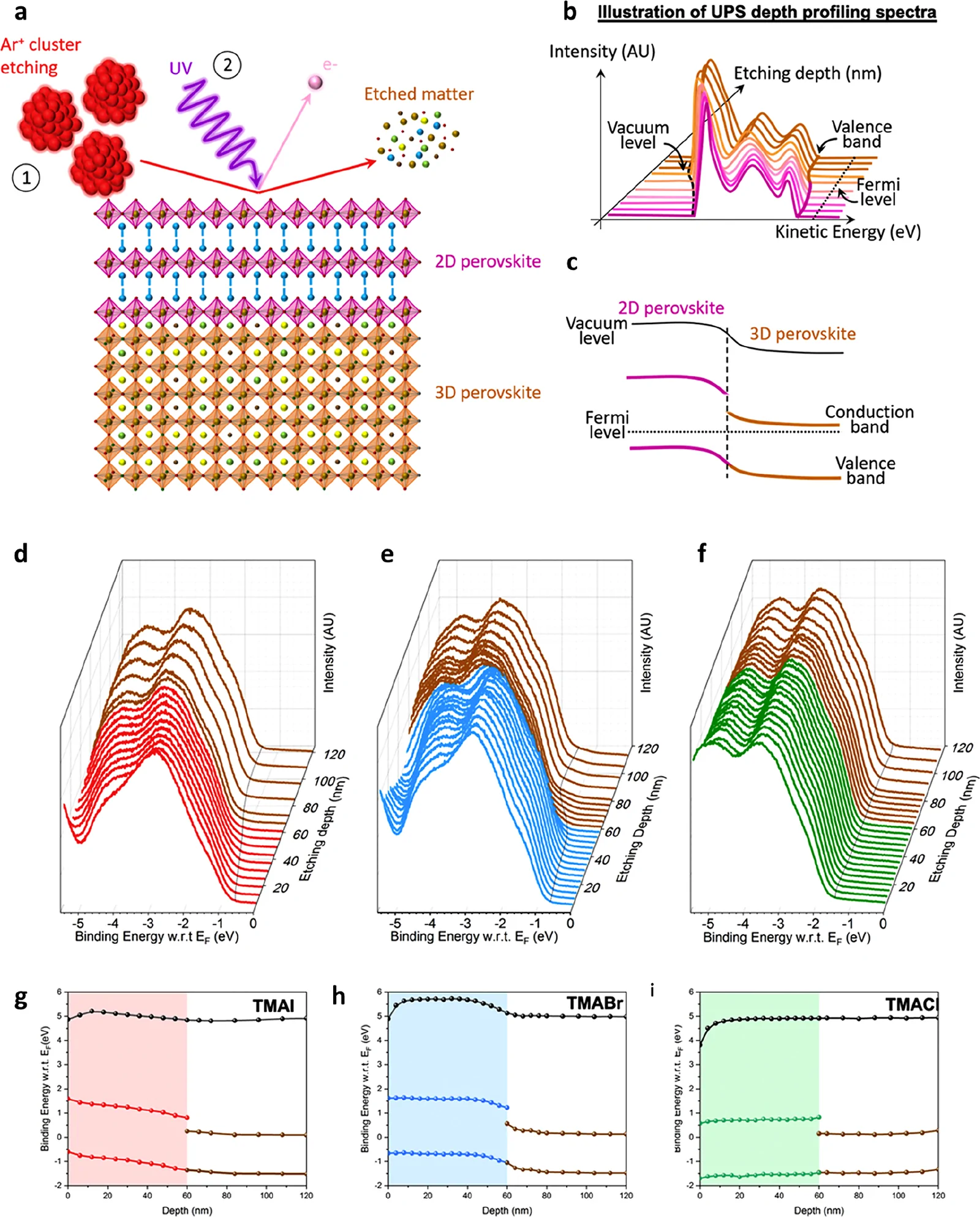
(a) Schematic representation of the UPS depth profiling technique, which combines (1) etching by Ar ion clusters with (2) UPS measurements. (b) Illustration of the evolution of the UPS spectra as a function of depth. (c) The corresponding energy level diagram is extracted from (B). (d–i) Measured UPS spectra and corresponding energetic level diagrams of 2-TMAI (d and g), 2-TMABr (e and h), and 2-TMACl (f and i) with the 3D perovskite layer. Note: dashed gray line in UPS spectra is a guide to the eye. Reproduced with permission from ref 264. Copyright 2021 Elsevier Inc.

Another advantage of UPS depth profiling is its ability to analyze the full-stacked device, enabling the tracking of energetic changes throughout the device’s lifetime. Kress et al. exploited this possibility to study the impact of ion migration on the energetic landscape of inverted architecture MAPbI_3_ devices.^265^ The authors revealed that upon biasing the device in the dark, iodine migrates toward the PCBM ETL, resulting in the band bending of the perovskite energy levels. Consequently, the device’s built-in potential was increased, resulting in higher *V*_*oc*_ and fill factor. Theoretical calculations by DFT and drift-diffusion modeling confirmed that this effect is persistent, i.e., present long after the bias has been removed. These results suggest that the energetic landscape of perovskite solar cells evolves during the device lifetime, opening new directions for future studies.

### 4.3 Inverse Photoemission Spectroscopy (IPES)

Inverse Photoemission Spectroscopy (IPES) is a complementary technique to UPS, as it facilitates the investigation of unoccupied states in the conduction band. In an IPES experiment, low-energy electrons are directed onto a sample and are captured into unoccupied states, followed by the emission of photons, which are subsequently detected.^266^ This allows the unoccupied density of states above the Fermi level to be mapped.^266^ This process reveals the conduction band structure and the energetic position of the CBM relative to the Fermi level. Combining the UPS with the IPES allows for a comprehensive description of the electronic band gap, including the determination of *IP*, *WF*, and *EA*.

Similar to UPS, the investigation of metal halide perovskites by IPES is affected by sensitivity to light, moisture, and oxygen, as well as vacuum-related and reproducibility issues inherent to the material; however, IPES also presents additional limitations that are specific to the technique itself. The most notable limitation, particularly for MHPs, is its inherently low signal intensity. The emission of a photon after electron capture is very unlikely, so spectra often require long integration times. This not only makes experiments time-consuming but also increases the risk of sample degradation, mainly due to prolonged exposure to the electron beam.^267^ The low-energy electron beam used in IPES can trigger ion migration, induce the formation of defect states, or even cause decomposition of the perovskite surface. Such alterations can obscure the intrinsic electronic structure and make the interpretation of unoccupied states uncertain.

A rigorous analysis was presented by Endres and co-workers,^224^ who combined UPS and IPES to determine both *E*_*V*_ and *E*_*C*_ of MAPbI_3_, MAPbBr_3_, and CsPbBr_3_ thin films. By aligning the experimental spectra with DFT calculations including SOC corrections, they extracted reliable values for ionization energy (5.2 eV) and electron affinity (3.6 eV) of MAPbI_3_ deposited on TiO_2_. In this investigation, the semilogarithmic representation was critical for visualizing the transport gap between valence and conduction bands. Later on, by combining UPS, IPES, and DFT, Tao et al.,^43^ were able to investigate the energy levels of 18 different tin- and lead-based perovskites. The observed trends in ionization energy, electron affinity, and bandgap (Figure 22) were rationalized using a tight-binding analysis, which revealed the key physical factors governing energy-level alignment. In particular, the effective atomic energy levels of the metal cations and halide anions, their hybridization strengths, and structural parameters such as lattice size and distortion were identified as decisive parameters. This systematic study helped shed light on fundamental aspects of perovskite energetics. Additionally, it serves as a starting point for optimizing interfaces in optoelectronic devices and engineering the energy levels of more complex mixed-halide compositions.

**Figure 22:**
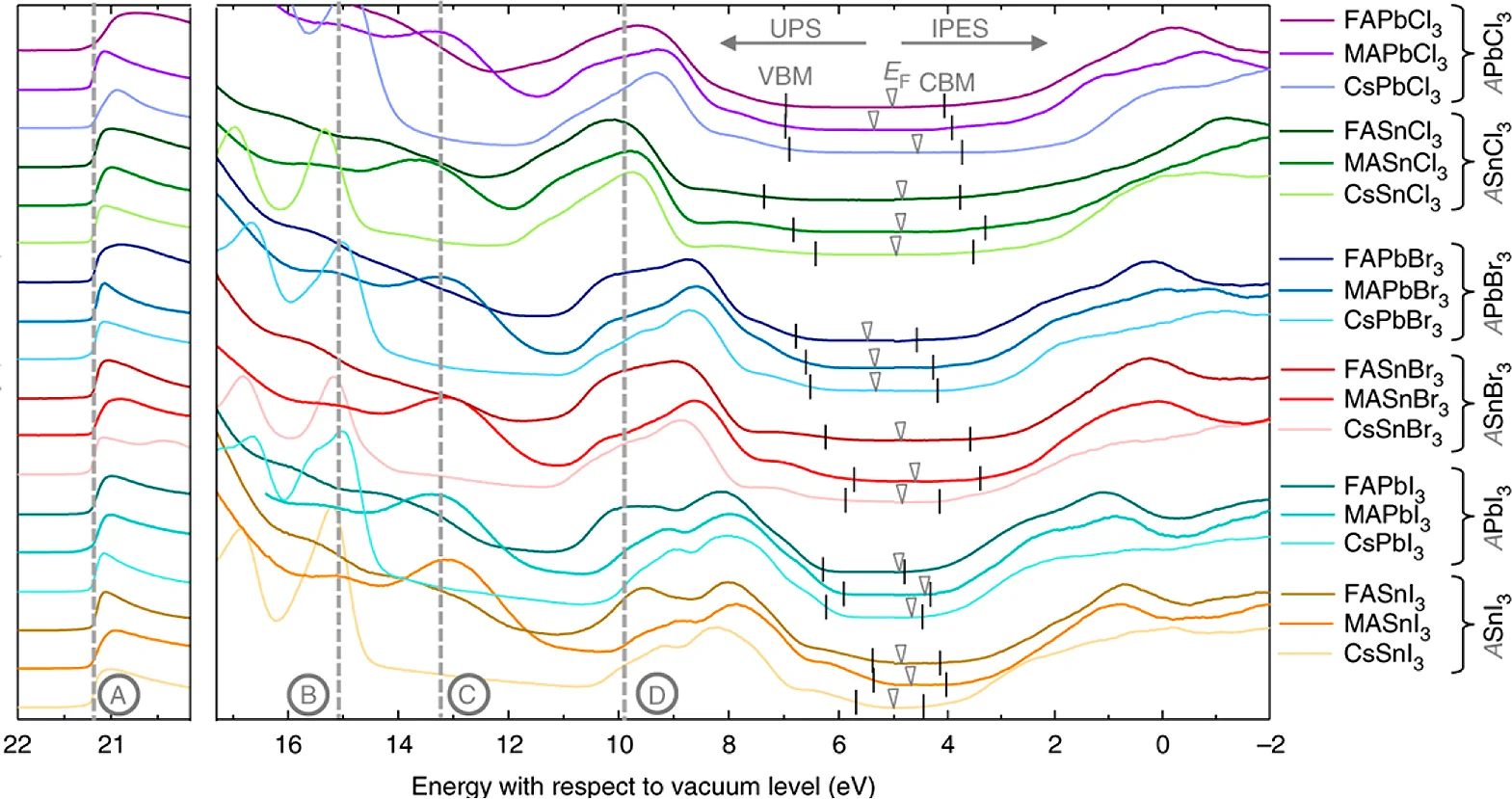
Representative UPS and IPES spectra of the 18 metal halide perovskites plotted on the linear scale. Reproduced with permission from ref 43. Copyright 2019 Springer Nature. Creative Commons Attribution 4.0 International License.

The complementarity of UPS and IPES is particularly useful for characterizing energy levels in 2D and quasi-2D perovskites, as well as their heterojunctions with 3D perovskites, which commonly provide better energetic alignment between the active layer and the CTL, and also serve as a moisture-protecting layer.^268,269^ For instance, for the quasi-2D Ruddlesden–Popper perovskites BA_2_MA_*n*–1_Pb_*n*_I_3*n*+1_ (BA: butylammonium, *n* = 1–5), the frontier energy levels have been established, revealing a type-I nested band gap heterostructure with its 3D counterpart, in which the band gap decreases with increasing *n* and converges toward the 3D MAPbI_3_ limit.^270^ A similar conclusion was made on other perovskite formulations.^271^ More about the formation of 2D and 3D interfaces and their impact on energetics will be discussed in Section 5.3.

### 4.4 X-ray Photoemission Spectroscopy (XPS)

#### 4.4.1 Spectral Acquisition

X-ray Photoemission Spectroscopy (XPS) is a widely used surface-sensitive technique, particularly useful for investigating band bending and interfacial dipoles, both of which are crucial for understanding energy alignment at heterojunctions. XPS operates by irradiating a material with X-rays (commonly Al Kα at 1486.6 eV) and measuring the kinetic energy of emitted photoelectrons.^190^ The most common method for analyzing electrons of varying kinetic energies involves an electron energy analyzer, typically composed of two biased hemispheres. Electrons with energies slightly above the pass energy (*E*_*P*_) are detected closer to the outer hemisphere, whereas those with lower energies are nearer to the inner hemisphere. By scanning the retardation voltage within the lens system, one can generate plots of electron intensity against energy.

This process allows the identification of the elemental composition and core-level binding energies, which are sensitive to local electronic potential and can therefore provide information about oxidation state and/or interfacial band bending when different materials are brought into contact to form an interface.^190^ Shifts in the XPS spectra can indicate, for instance, charge redistribution across the junction, which results from differences in work functions or Fermi level positions before contact. As discussed for UPS (Section 4.2.3.4), these shifts can be monitored as a function of depth using techniques such as sputtering or angle-resolved XPS. However, these methods are destructive and only provide information in the dark and at equilibrium. In-operando XPS on a device cross-section is also challenging, because the X-ray spot size is larger than the typical PSC cross-section. The group of Jägermann addressed this limitation by preparing a tapered cross-section (TCS), in which the device is cut at a shallow angle to create an extended cross-section that can be scanned with a micrometer-scale beam.^272,273^ With this geometry, they monitored the continuous core-level shifts across the stack and directly mapped the built-in potential (Figure 23a). The difference between the dark and illuminated scans gave the local photopotential, revealing that, for their mesoscopic device, the photovoltage originates at the perovskite/ETL interface, with an XPS-derived value of −0.64 V. As the time-consuming TCS-XPS measurement is likely to induce degradation, the same sample type was characterized by faster TCS-UPS, which yielded a photoinduced shift of 1.2 eV, consistent with the device *V*_*OC*_ ≈ 1.1 V. Using material-specific binding energy difference between core level and valence band maximum (Δ*E*_*BCV*_), they reconstructed the complete valence band alignment across the device (Figure 23b), confirming an *n*-type perovskite and showing that the built-in potential drops mainly inside the hole-transport layer.

**Figure 23:**
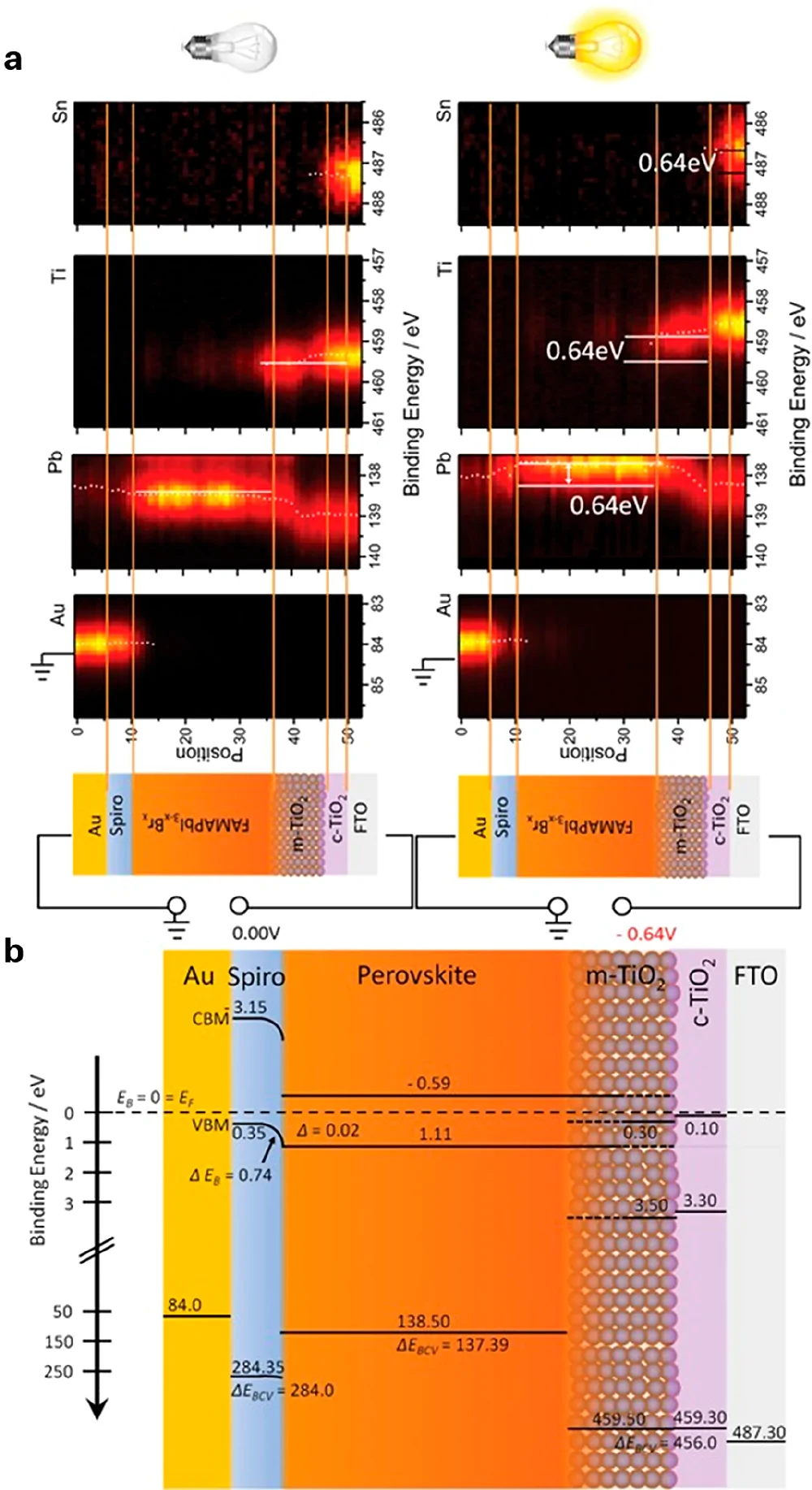
(a) XPS intensity contour plots performed on the tampered cross-section of the device stack shown below. The scans of the Au 4f_7/2_, Pb 4f_7/2_, Ti 2p_3/2,_ and Sn 3d_5/2_ core levels measured at open-circuit conditions, in the dark (left) and under illumination (right). (b) Schematic band diagram of the same device in the dark, obtained from the core-level map in a, and published bandgap values from charge-transporting layers to obtain the CBM. Reproduced with permission from ref 272. Copyright 2020 John Wiley & Sons Inc.

XPS is also effective in probing interfacial dipoles.^226^ These dipoles arise from abrupt changes in electrostatic potential at the interface, often due to surface treatments, molecular interlayers, or chemical reactions (see Section 5). Interfacial dipoles can alter the local vacuum level and shift energy levels, thereby affecting overall energy-level alignment.^155,274,275^ Such dipoles manifest as systematic shifts in the vacuum level and core-level positions across the interface, which can be resolved by carefully analyzing changes in binding energy before and after interface formation.^276,277^

#### 4.4.2 Limitations and Pitfalls

Careful interpretation of the XPS data is essential because the measurement itself, i.e., exposure to energetic radiation in vacuum, can induce surface modifications to the sample. This issue has been recognized since the earliest PES investigations of perovskite solar cells, in which radiation-induced defect formation and surface chemical degradation posed significant challenges.^202,278^

##### 4.4.2.1 X-ray Illumination

High doses of X-ray radiation during the spectra acquisition using, for example, synchrotron sources, were found to be the primary cause of the sample degradation, impacting mainly defect formation, as demonstrated by Stuckelberger et al.^279^ What is observed in short time scales at synchrotron facilities, can take hours in a standard laboratory, and even longer for poorly monochromatized radiation sources. For instance, an early study showed no significant changes in the perovskite’s core-level spectra after up to 15 h of X-ray irradiation in the absence of additional illumination.^280^ However, other studies report notable compositional changes upon X-ray exposure.^281^ During the early stages of irradiation, up to approximately 4.5 h, these changes are characterized by the formation of iodine and methylammonium vacancies, *V*_*I*_ and *V*_*MA*_, respectively. Steirer et al. suggest that these vacancies act as self-compensating defects, counterbalancing each other and leaving the positions of valence and core levels largely unaffected. This observation may reflect the proposed defect tolerance of perovskites at early irradiation stages and could explain the minimal X-ray beam damage reported in some XPS studies.^214^

Over even longer exposure times, however, the surface composition evolves, with losses of methylammonium and iodine corresponding to the degradation of the perovskite into its precursors, for example, of MAPbI_3_ into PbI_2_ and MAI.^282,283^ Additionally, the phenomenon of the Pb^2+^ reduction to metallic lead is inconsistently reported in the literature, even under ostensibly similar experimental conditions,^284,285^ raising questions about both the underlying degradation pathways and the strategies to mitigate beam-induced effects. An early work by Shkrob and Marin, using electron paramagnetic resonance spectroscopy, attributed the emergence of Pb^0^ clusters to radiolysis initiated by high-energy X-ray irradiation.^286^ However, other factors may also contribute, including photolysis under visible illumination^179^ or degradation processes linked to the intrinsic instability of the perovskite phase itself. It is noteworthy that light-induced degradation is negligible in nitrogen-filled environments and primarily occurs under vacuum or in atmospheres containing at least 1% oxygen.^287,288^

Practical measures to minimize artifacts caused purely by X-ray induced modifications have long been limited to coarse guidelines, such as minimizing acquisition times (<30 min) and protecting samples from light exposure, as suggested by McGettrick et al.^283^ More recently, however, systematic studies have begun to disentangle the mechanisms of Pb^0^ formation under XPS conditions. Kerner and co-workers^289,290^ demonstrated that this process cannot be explained solely by PbI_2_ photolysis. Instead, their results point to the formation of Pb–N species (lead amides) as an intermediate step, with the reaction significantly accelerated in the presence of catalytic overlayers such as gold. In particular, pristine MAPbI_3_ and PbI_2_ films remained stable under irradiation, whereas MAPbI_3_ covered with a thin gold film showed pronounced Pb^0^ generation. This finding supports a mechanism consistent with underpotential deposition, where Au catalyzes the reduction of Pb^2+^ to metallic lead.

In another study, X-ray illumination was found to significantly alter the valence band density of states, leading to a pronounced increase in occupied gap states and a more pronounced exponential band tail. This highlights that X-ray exposure can induce defect states and band-edge broadening in halide perovskites, which must be carefully considered when interpreting photoemission measurements.^247^

##### 4.4.2.2 Nonmonochromatized X-ray Sources

The use of nonmonochromatized X-ray sources, particularly at high anode power, can induce bremsstrahlung that leads to considerable energy-level shifts in perovskite films.^291^ In contrast, measurements performed with monochromatized X-rays show negligible band realignment within typical operating conditions, underscoring the importance of careful source selection. More broadly, the interpretation of XPS data at perovskite interfaces must account for possible charge accumulation and interfacial dipoles, which can shift both core levels and valence band onsets relative to the vacuum level. If such realignment effects are overlooked, energy-level diagrams derived from XPS may not accurately reflect the true interfacial energetics in working optoelectronic devices. Therefore, establishing standardized measurement protocols that minimize or explicitly account for band realignment is essential to achieving reliable, reproducible insights into the perovskite electronic structure.

##### 4.4.2.3 Data Misinterpretation

Li et al.^292^ have highlighted four main types of errors that frequently compromise the reliability of XPS analysis in halide perovskites. The first is a misinterpretation of the XPS mechanism, often arising from a flawed understanding of chemical-shift origins. For example, some studies incorrectly attributed higher *E*_*bin*_ shifts of Pb 4*f* levels to increased local electron density, whereas, in reality, such shifts reflect the influence of electronegativity and core-hole screening rather than direct changes in valence charge density. The second error is misinterpretation due to disturbed chemical environments, leading to contradictory results from unregulated or inconsistent experimental operations. The same surface treatment or additive has been reported to induce both upward and downward Pb 4*f* shifts in different studies, suggesting that experimental artifacts, rather than genuine chemical differences, are responsible for the discrepancies. A third source of error stems from a lack of awareness of the properties of the passivator. Many works attribute binding energy shifts solely to interactions between passivating agents and undercoordinated Pb, without establishing a consistent mechanistic link between the expected electron donation and the observed shifts. As a result, contradictory interpretations have been proposed. For instance, a Lewis base reported to increase electron density at Pb paradoxically associated with an upward *E*_*bin*_ shift, which would indicate the opposite. Finally, misquoted references have been identified as a significant issue. In several cases, conclusions from earlier works have been uncritically cited in contexts where the experimental conditions or observed trends were fundamentally different, leading to biased or logically inconsistent interpretations. A notable example is the repeated assumption that quaternary pyridinium acts as a Lewis base passivator. However, the lone pair of pyridine is no longer available in quaternary pyridinium (e.g., methylpyridinium), so it cannot act as the electron donor.

Together, these four categories of errors, named mechanistic misinterpretations, artifacts from disturbed chemical environments, incorrect assumptions about passivator properties, and careless citation practices, underscore the necessity for rigorous calibration, careful data interpretation, and a solid grounding in both the fundamentals of XPS and the chemistry of halide perovskites.

### 4.5 Hard X-ray Photoemission Spectroscopy (HAXPES)

Conventional X-ray photoelectron spectroscopy (XPS) is a surface-sensitive technique with a typical probing depth of about 5–10 nm, determined by the inelastic mean free path of photoelectrons excited by Al Kα or Mg Kα sources (∼1–1.5 keV). This makes XPS ideal for detecting surface composition, chemical states, and band bending, but it limits its ability to probe buried interfaces or bulk properties. Hard X-ray photoelectron spectroscopy (HAXPES) uses much higher photon energies (typically 3–10 keV), increasing the photoelectron kinetic energy and extending the probing depth to 20–50 nm or more, depending on the material. As a result, HAXPES can access deeper interfaces, bulk electronic structure, and buried junctions with reduced surface sensitivity. However, its reduced cross sections result in lower signal intensity and poorer energy resolution than in lab-based XPS, making subtle chemical shifts harder to resolve. Together, XPS and HAXPES provide complementary information: XPS is optimal for surface chemistry and band alignment at the top few nanometers, while HAXPES enables nondestructive access to buried layers and deeper electrostatics.

In-operando HAXPES studies further highlight that interfacial photovoltage generation and band-alignment changes can be directly monitored in functioning perovskite solar cells in ways that conventional XPS cannot. Because hard X-rays provide a much greater probing depth, HAXPES allows simultaneous detection of buried layers, such as the gold back contact, the HTM, and the perovskite, without the need for destructive sample thinning or tapered cross sections. In the device operated at open circuit, illumination induced distinct core-level shifts across these buried layers, with the most significant shift observed at the HTM/Au interface, demonstrating that a substantial fraction of the photovoltage originates at this contact.^133^ Complementary valence band spectra revealed that the band alignment itself changes under illumination, consistent with illumination-induced charge redistribution and chemical modifications, including ion migration such as iodide diffusion into the HTM. In contrast to conventional XPS, which is too surface-sensitive to access buried interfaces without destructive preparation, HAXPES provides a direct view of the internal potential profile, enabling operando analysis of voltage generation, interfacial band bending, and chemical evolution under realistic working conditions. Figure 24 shows time-resolved HAXPES intensity maps of the Cs_0.17_FA_0.83_PbI_3_/P3/Au interface (P3 being a polymertic ETL) under short-circuit conditions in the dark (top row), where the binding energies of all core levels remain essentially constant. In contrast, under illumination at open-circuit (middle row), the core levels shift toward higher binding energies relative to short-circuit. This shift is attributed to photovoltage, which splits the electrochemical potential between the grounded front contact (FTO) and the Au back electrode. As a result, the back contact becomes positively biased, increasing the energy barrier for photoelectron emission and leading to an apparent shift toward higher binding energies. For comparison, averaged core-level spectra measured during short-circuit in the dark and under open-circuit before, during, and after illumination are shown below. This study demonstrates that HAXPES can determine where in the stack the photovoltage is generated and how the internal electric field redistributes under illumination, providing capabilities essential for understanding the full device physics. Related measurements on LED devices showed behavior consistent with that observed here.^293^

**Figure 24:**
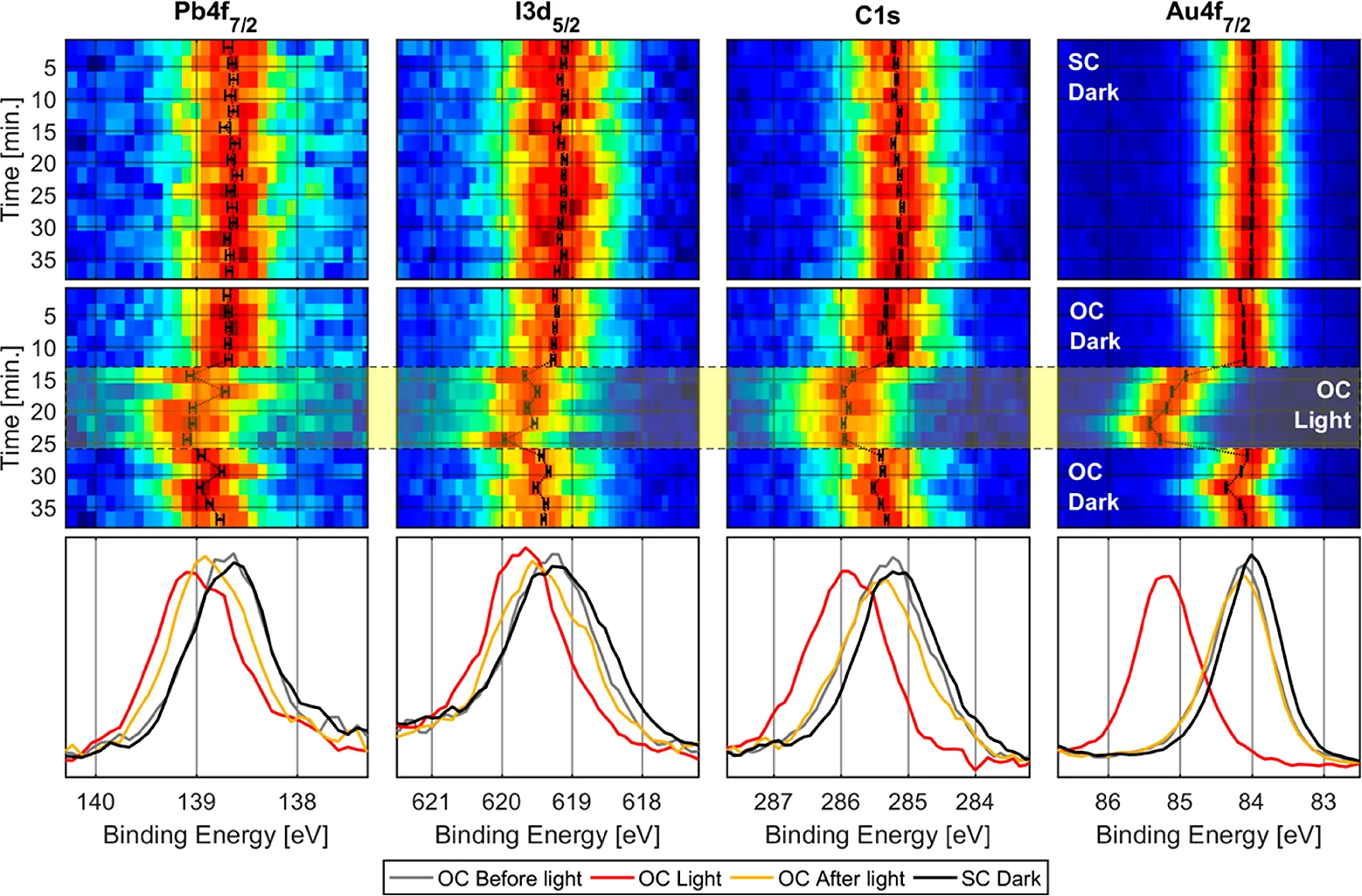
Top: HAXPES intensity contour map of the Cs_0.17_FA_0.83_PbI_3_/P3/Au interface under short-circuit conditions in the dark. Middle: Corresponding maps under open circuit, recorded in the dark and under illumination. Bottom: Averaged core-level spectra for short circuit in the dark and for open circuit before, during, and after illumination. Binding energies are referenced to the Au 4*f* level measured under short-circuit dark conditions (84 eV). Reproduced with permission from ref 294. Copyright 2023 Royal Society of Chemistry.

### 4.6 Contact Potential Difference Measurement (CPD)

#### 4.6.1 Working Principle

The CPD quantifies the difference in work function between a reference probe and a sample surface. When two conductive materials are brought into electrical contact, electrons flow from the material with the lower work function to that with the higher work function until their Fermi levels equilibrate, giving rise to an electrostatic potential difference. By measuring this potential difference, CPD techniques provide direct access to the sample’s surface work function relative to the probe. This principle underpins methods such as the Kelvin probe, which operates in a noncontact and nondestructive manner, making it particularly well-suited for delicate or air-sensitive materials. In practice, an oscillating reference probe induces an AC current proportional to the CPD, which is compensated by applying an external bias, allowing the work function difference to be determined with high sensitivity. As a result, CPD measurements are widely employed to map spatial variations in work function across heterogeneous surfaces, monitor changes induced by surface treatments, doping, or adsorption, and investigate the electronic properties of metals, semiconductors, and organic films. Owing to their sensitivity and spatial resolution, these techniques provide valuable insight into interfacial energetics and are widely used to optimize interfaces in devices such as solar cells, transistors, and sensors. This measurement can be coupled with scanning probe microscopy, often using atomic force microscopy (AFM), providing a spatial resolution of the work function across the measured film.

As an advanced AFM mode, Kelvin probe AFM (KPFM) measures the CPD between a conductive probe and the sample surface. KPFM is particularly valuable for mapping the electronic properties of heterogeneous and multilayered structures common in PSCs, where interfaces between the perovskite absorber and charge-transport layers are essential for efficient charge separation and extraction. By scanning across different layers or grains, KPFM can visualize local variations in surface potential that arise from differences in material composition, crystallinity, grain boundaries, interfacial dipoles, or trap states. In terms of energetic alignment, KPFM is widely used to determine the relative work function differences across interfaces and among various functional layers in complete or partial device stacks. This information can, for instance, be used to assess whether the energy levels are well-aligned for effective electron and hole transfer. One of KPFM’s key advantages is its ability to operate in ambient or controlled environments and to measure devices under both dark and illuminated conditions. This capability enables the study of photoinduced charge redistribution and surface potential shifts. It has been widely used to examine in-operando the cross-section of organic^295–297^ and inorganic^298^ solar cells.

In early studies, KPFM was used to investigate the electric field distribution in the working device based on the mesoscopic structure, which could be derived from the internal electrical potential distribution (Figure 25).^299^ The study suggested asymmetrical hole accumulation at the perovskite/HTL interface, pointing it as the energy barrier and the reason for limited carrier extraction. More recently, this technique has been used to investigate the efficiency of the charge extraction by different charge-transporting layers.^300,301^ The original findings provided a boost to later studies on the origins of hysteresis in PSCs, with the discussion always starting with charge accumulation at the interface of the perovskite and the charge-selective material.^182,302,303^ Qualitatively, these results are consistent with the formation of an interfacial dipole layer, as discussed in Section 3.2.2 and shown in Figure 12g, suggesting that the effects of ion migration, defect passivation, and energy band alignment are difficult to disentangle.

**Figure 25:**
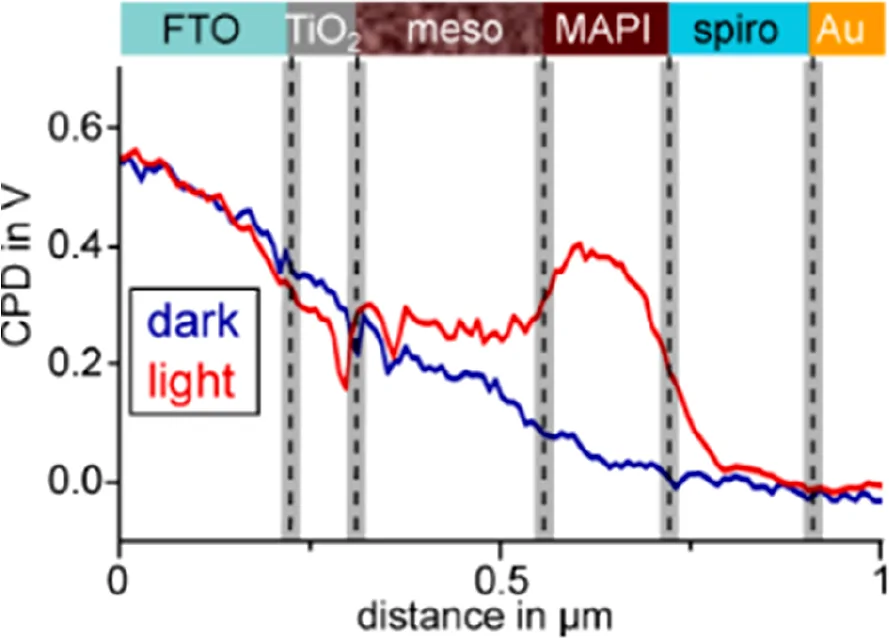
Contact potential difference of the cross-section of the mesoscopic MAPbI_3_-based solar cell in the dark and under illumination at short circuit. Reproduced with permission from references^299^ and.^304^ Copyright 2014, Springer Nature Limited and 2018 Beilstein Institute, Creative Commons Attribution License, respectively.

KPFM has also proven to be a powerful tool for probing the electronic and ionic properties of the perovskite film itself.^305^ Correlative KPFM and scattering-type Scanning Near-field Optical Microscopy (s-SNOM) studies on MAPbI_3_ have revealed that the local built-in potential at GBs drives electrons toward the GBs and holes toward the grain interiors (GIs), which promotes charge separation and reduces recombination losses.^69^ Further, KPFM performed on mixed-cation perovskite solar cells under applied bias has shown that grain boundaries also play a role in ionic dynamics.^306^ The application of bias, regardless of polarity, alters the CPD, with negative bias producing more substantial shifts due to enhanced ion accumulation at the interfaces. After removing the bias, CPD relaxation typically took hundreds of seconds, directly evidencing slow ion migration.^307^ Notably, this relaxation occurred faster at GBs than at GIs, which was attributed to higher ionic diffusivity and/or stronger ion segregation at the GBs. Together, these findings demonstrate that KPFM not only resolves the electronic contribution of GBs to charge-carrier separation but also highlights their key role as channels for ion migration in perovskite films.

It must be mentioned that frequency mode KPFM (FM-KPFM) consistently provides more quantitative and reliable surface potential data than amplitude mode KPFM (AM-KPFM).^304^ Since, in FM mode, detection is dominated by the tip–sample interaction, with minimal influence from the cantilever, it yields more accurate relative potentials and more reproducible absolute values. In contrast, AM-KPFM suffers from significant cantilever contributions, causing lateral averaging, reduced potential contrast, and shifts in the absolute potential, particularly in the presence of stray fields. For these reasons, FM-based methods are recommended for quantitative device characterization, while AM lift mode should generally be avoided unless stray-field mitigation strategies are carefully implemented.

#### 4.6.2 Limitations and Pitfalls

The susceptibility of MHPs to light- and vacuum-induced degradation can lead to artifacts in CPD measurements. Under supragap illumination during KP-SPV experiments, halide loss from the perovskite surface can occur, leading to irreversible changes to the surface work function. At the same time, these degradation processes can also alter the Kelvin probe tip, further compounding the risk of artifacts. If not carefully recognized and controlled, these effects may result in misleading conclusions about the intrinsic electronic properties of the perovskite. Figure 26 shows the evolution of the *WF* of the MAPbBr_3_ layer as well as of the Kelvin probe tip when left in the vicinity of a degrading perovskite. Sample illumination with red light for 60 s led to no change in measured values, while the illumination with green light for 15 s resulted in the increase of the perovskite’s *WF* by 0.10 eV, as well as the increase of the *WF* of the tip by 0.18 eV. Neglecting the tip calibration would lead to an erroneous conclusion that the *WF* of the perovskite decreases under illumination.^221^ Therefore, stringent precautions and measurement protocols are required to minimize degradation and ensure reliable CPD data. In this context, it is important to emphasize that surface contamination can readily introduce artifacts in CPD measurements, as this technique is inherently surface-sensitive. Adsorbed species, such as hydrocarbons, residual solvents, oxygen, or moisture, can modify the surface dipole and alter the local potential. Consequently, the measured CPD should be regarded as proportional to, rather than strictly equal to, the difference between the Fermi levels of the sample surface and the reference probe.

**Figure 26:**
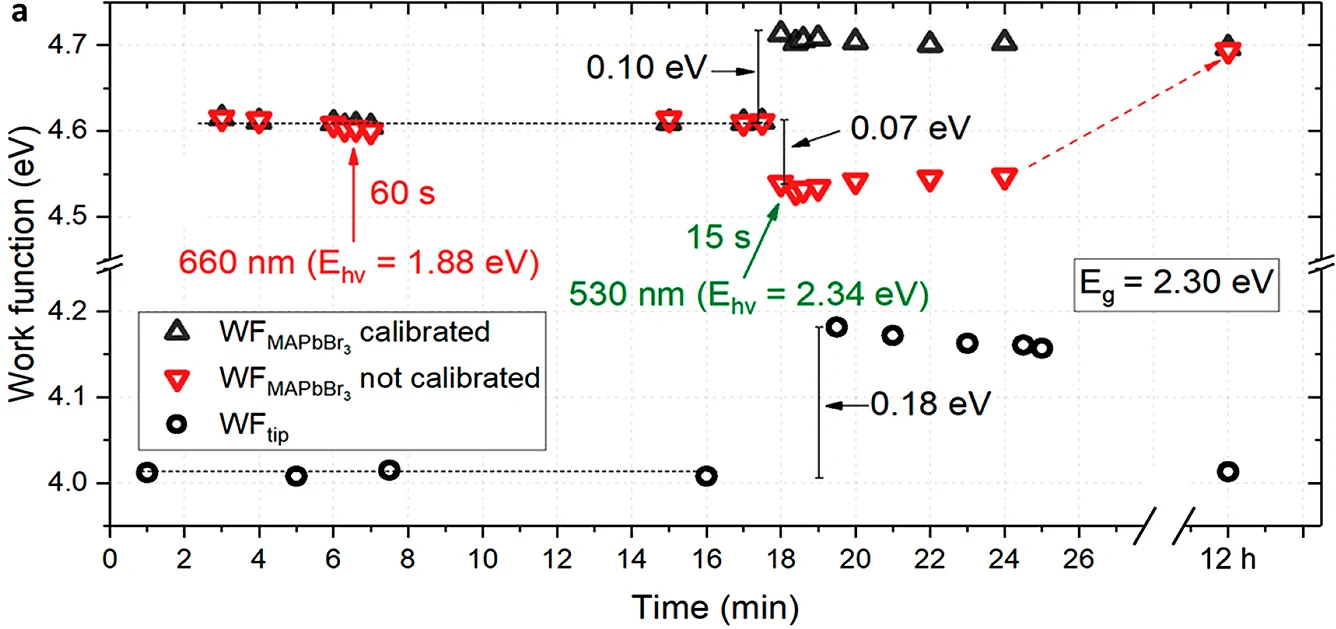
Work function of MAPbBr_3_ before, during and after illumination, measured using calibrated (black triangles) and noncalibrated (red triangles) Kelvin probe as well as of the Kelvin probe tip (black circles). Reproduced with permission from ref 221. Copyright 2020 John Wiley & Sons, Inc.

### 4.7 Concluding Remarks on the Characterization of the Energy Level Alignment

While energy-level characterization can be performed using UPS and IPES, the results obtained from these methods tend to depend strongly on sample preparation, ambient storage conditions (air, nitrogen, vacuum), and measurement conditions. Following Béchu et al.,^196^ the correct data interpretation must consider the following aspects:

- *Sample History and Extrinsic Factors*: The entire sample stack, including substrates, architecture, and transport layers, along with the growth conditions, is crucial for interpreting PES results. The presence of compositional inhomogeneities can also affect electronic properties and needs to be tracked. Sample history, particularly prior exposure to air, varying humidity, and illumination, can significantly alter the results and should be reported. Air exposure should be minimized because oxygen can generate superoxide species that degrade perovskite materials and cause artificial surface doping. Oxygen is especially detrimental for Sn-based perovskites, where Sn^2+^ is susceptible to oxidation to Sn^4+^.
- *Chemical Reactions and Degradation*: MHP films can undergo extensive chemical reactions both before and during the PES experiment. X-ray and UV light exposure during data acquisition can cause degradation, leading to apparent changes in chemical composition and affecting quantitative analysis. To mitigate these artifacts, the shortest possible acquisition times are recommended.
- *Data Acquisition and Analysis Challenges*: While PES is a powerful tool for determining key electronic properties, such as band onsets and the Fermi level position, data acquisition and analysis pose specific challenges. The accurate definition of the VB edge is difficult due to the low DOS and the presence of defect states. Accurately fitting the band edge is difficult due to its low value. Best practices involve using theoretical modeling and corroborating findings with complementary techniques, such as Scanning Tunneling Spectroscopy (STS) or KPFM. Although these techniques cannot resolve fitting band-edges on their own, they are valuable complementary tools. It is also important to note that Fermi level pinning to defect states induced by the measurement conditions can alter the pristine MHP surface.

Concerning the future direction of energy level characterization, a notable and exciting possibility is in-operando PES measurements, in which PES analysis is performed in parallel with other controlled experiments under modulated electrical and/or light bias. This approach aims to capture relevant physical and chemical properties, such as work function and stoichiometry, on time scales compatible with chemical processes, such as ion migration. Additionally, in-operando experiments can capture changes in energy levels that occur during the operation of solar cells. Understanding these changes can provide insights into the performance loss and degradation commonly seen in MHPs-based optoelectronic devices. Furthermore, these experiments enable tracking of dynamics at interfaces, highlighting the importance of careful management, including the implementation of passivation strategies, to minimize reactivity and reduce the defect density. Another direction is near-ambient-pressure PES, which permits measurements during interaction with various gases, allowing quantification of reactivity and the impact of degradation on energy alignment.

When discussing the energetic alignment of the perovskite devices, it is important to note that the band edge energies of different materials are sometimes shown together, even though they were measured by different methods. For instance, HOMO and LUMO levels of organic materials (often used as charge-selective materials in PSCs) are commonly estimated using electrochemical techniques. The differences in energy values obtained using different methods can, in some cases, exceed 1 eV for the same organic species.^308^ Combining electrochemical and spectroscopic methods to compare adjacent materials would require assuming that the difference in solvation effects between an electrolyte solution and a solid-state environment is constant across different compounds, which is not always the case. In that case, the information from the cyclic voltammetry is a crude estimate at best and should be regarded as such.

Additionally, there is suspicion that certain relevant molecules used for solar cell fabrication, such as 2PACz, which is commonly employed as a hole-transport material in inverted *p-i-n*-type PSC, may undergo electrochemically irreversible degradation. This can manifest as a decrease in the peak current upon repeated cycling^309^ or as an asymmetric cyclic voltammogram.^310^ While it is still under debate whether such behavior is reproduced in solid-state devices, the electrochemical instability of SAMs can indeed be a complication.

Moreover, the use of optical data should be considered with great care, as it neglects the exciton binding energy.^311^ This omission is especially severe for organic semiconductors, where this value is notably nonzero. Consequently, many band diagrams in the literature showing alignments involving organic species should be regarded as approximations and subject to uncertainty.

## 5 Interfaces and Energetic Alignment: Functions, Mechanisms, and Control

Several factors can contribute to the success or malfunction of a perovskite solar cell. These factors include the energy band alignment of the materials in the stack, the defect landscape at the interfaces, and the dynamics of ionic motion. These phenomena are, in practice, difficult to disentangle; hence, all of them are often proposed as possible mechanistic explanations of the experimentally observed changes in *V*_*OC*_, *FF*, and *J*_*SC*_. Metal halide perovskites, with their soft ionic lattice, electrically active defects, and mobile ions, are known for a dynamic evolution of the energetics at the interfaces of the device. As discussed earlier, the energy levels of their band edges can change when in contact with the extraction layers and electrodes, due to defect formation or passivation, chemical reactions, or redistribution of mobile charge carriers and ions. This dynamic landscape can be controlled by targeted strategies, starting with selecting the perovskite composition and the electrode materials with work functions suitable for efficient, selective charge extraction. A common approach to modulate the interface properties is to add very thin layers between the perovskite and the charge-selective contacts. These interlayers can alter the interface chemically and/or energetically. However, in practice, it is difficult to separate these two effects. This is because the interfaces in MHP are naturally very reactive, so almost any material that interfaces with the perovskite can affect both its surface chemistry and surface energy.^312–314^

In the following Section, we will examine the role of interfaces in PSCs, including the mechanisms that can occur within them and affect their performance, and outline mitigating strategies tailored to the targeted mechanism. We will focus on methods for adjusting energy levels, such as shifting the vacuum level, altering the work function, or modifying the energetics of the charge-selective layer relative to the perovskite. Furthermore, we will examine methods to passivate defects, which aim to reduce the nonradiative recombination rate in the device. While these two strategies are often synergistic in practice, they arise from distinct physicochemical mechanisms, and separating them conceptually helps in understanding their individual contributions to device performance.

### 5.1 Compositional Engineering

In PSCs, the very first step in designing an efficient device is selecting a suitable perovskite composition, which defines the band-edge energy levels, and selecting the materials for selective contacts, for which work function is a key property. Appropriate energy-level alignment facilitates unidirectional charge extraction and enables the formation of built-in fields at perovskite/contact interfaces, facilitated by the ETL/HTL. A diagram of the *IE*s and *EA*s of different MHPs together with the *WF*s of the electrode materials is presented in Figure 27. As shown, composition engineering offers powerful leverage to tune the perovskite electronic structure and transport properties. In ABX_3_ perovskites, the B- and X-site cations have a strong influence on the electronic properties, such as the bandgap, carrier mobility, and diffusion length. For example, already early works reported that a partial replacement of I^–^ with Cl^–^ in MAPbI_3_ produces MAPbI_3–*x*_Cl_*x*_ with greatly enhanced carrier diffusion coefficients (0.042 cm^2^ s^–1^ for electrons and 0.054 cm^2^ s^–1^ for holes) and diffusion lengths (1069 nm for electrons and 1213 nm for holes), compared with only 129 and 105 nm for MAPbI_3_, respectively, suggesting strong compatibility with planar CTL-free device configurations.^315^

**Figure 27:**
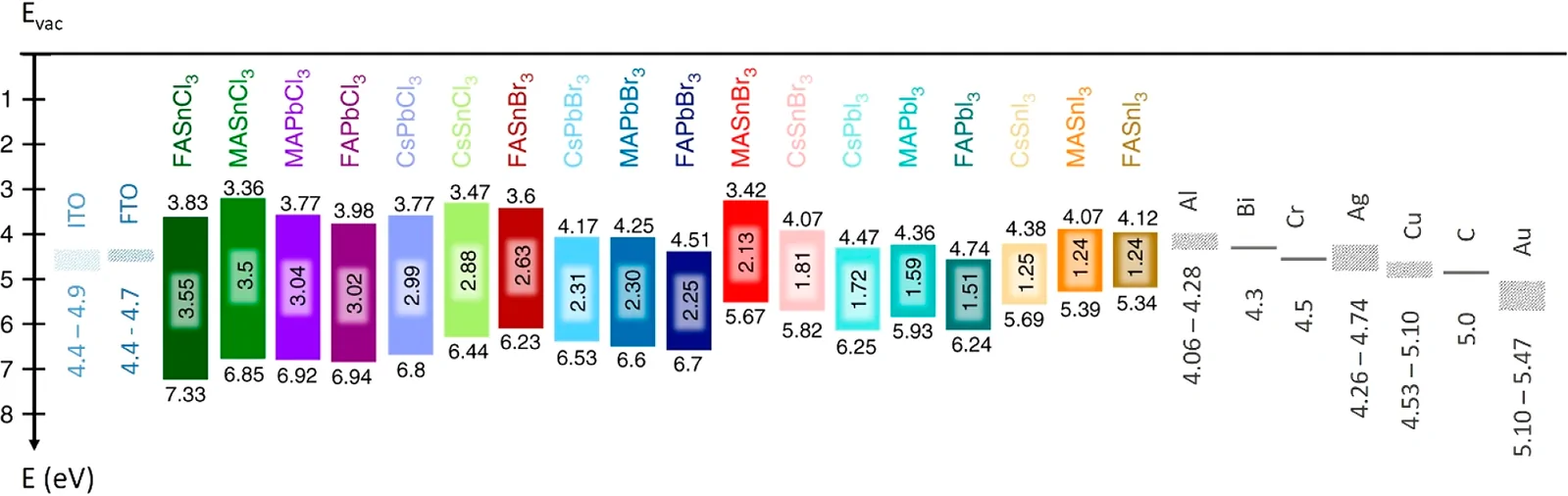
*IE* and *EA* of different perovskites and the *WF*s of commonly used electrodes. For most materials, work function depends strongly on surface treatment, crystal orientation, and contamination/adsorbates, so reported values often span a small range, which is represented by a shaded area. Reproduced in part from ref 43. Creative Commons CC BY license. Springer Nature.

### 5.2 Interfacial Dipoles

At a fundamental level, the presence of a work-function asymmetry between the perovskite and the charge-selective contacts is an important aspect for the operation of a solar cell. One way to generate or improve such asymmetry is to introduce a thin interlayer of organic, inorganic, or polymeric materials between the perovskite and the electrodes or the CTL, creating a dipolar layer that shifts the surface work function and helps direct the photogenerated charge carriers toward the corresponding electrodes. For example, when the dipole arrow points from the perovskite (δ^–^) to the contacting layer (δ^+^), the resulting positive dipole produces a downward shift of the vacuum level at the contact region, decreasing the work function (Figure 28a), which improves the electron extraction when the perovskite interfaces with an ETL. Conversely, when the dipole arrow points from the contacting layer (δ^–^) to the perovskite (δ^+^), the resulting negative dipole produces an upward shift of the vacuum level at the interface, increasing the work function (Figure 28b), which aids hole extraction at the perovskite-HTL interface. These changes are purely electrostatic and do not necessarily require the formation of a chemical bond, although chemical interactions may coexist. In practice, interfacial dipoles typically induce potential shifts of tens to hundreds of meV, sufficient to tune charge-extraction barriers and improve selectivity.

**Figure 28:**
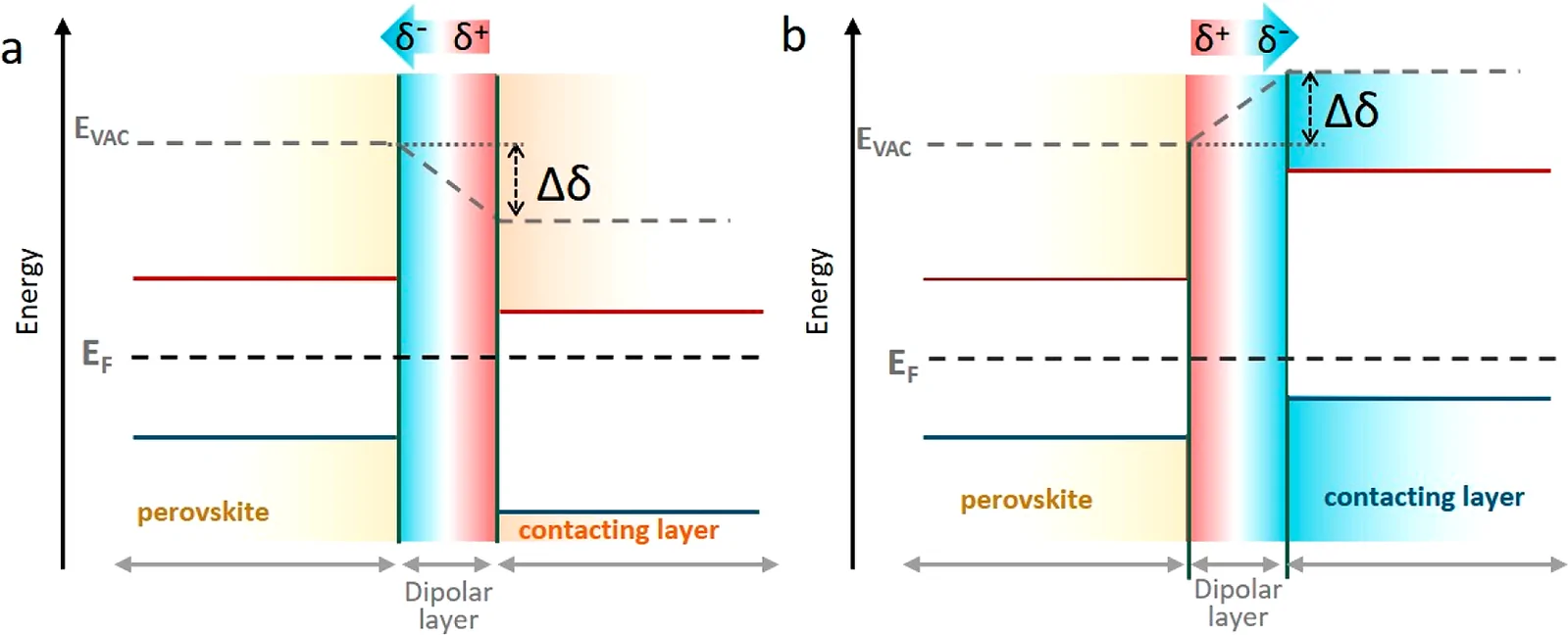
Schematic illustration of interfacial dipole engineering in perovskite solar cells. (a) Positive surface dipole, commonly employed at the ETL/perovskite interface. (b) Negative surface dipole, commonly used at the HTL/perovskite interface.

Regarding dipole formation at interfaces, an important class of molecules that has attracted attention is self-assembled monolayers (SAMs). These molecules comprise three basic units: an *anchor* group that binds the molecule to a substrate, a *spacer* unit that drives the packing of the molecule, and a *terminal* group that modifies the surface chemistry (Figure 29a).^316^ Although comprising the term “monolayer” in its name, it is widely recognized that they do not always form a monolayer of molecules; instead, in some cases, several layers of molecules are stacked on top of each other. The formation of a dipolar interlayer between the SAM/perovskite can be divided into three categories: (i) the intrinsic dipole moment along the backbone of the SAM, (ii) the interface dipole moment between the anchor and terminal groups, and (iii) the bond dipoles resulting from the interaction between the substrate and the anchor group.^155^ Different chemical groups used as anchors, spacers, or terminal groups will alter the dipole at the interface.

**Figure 29:**
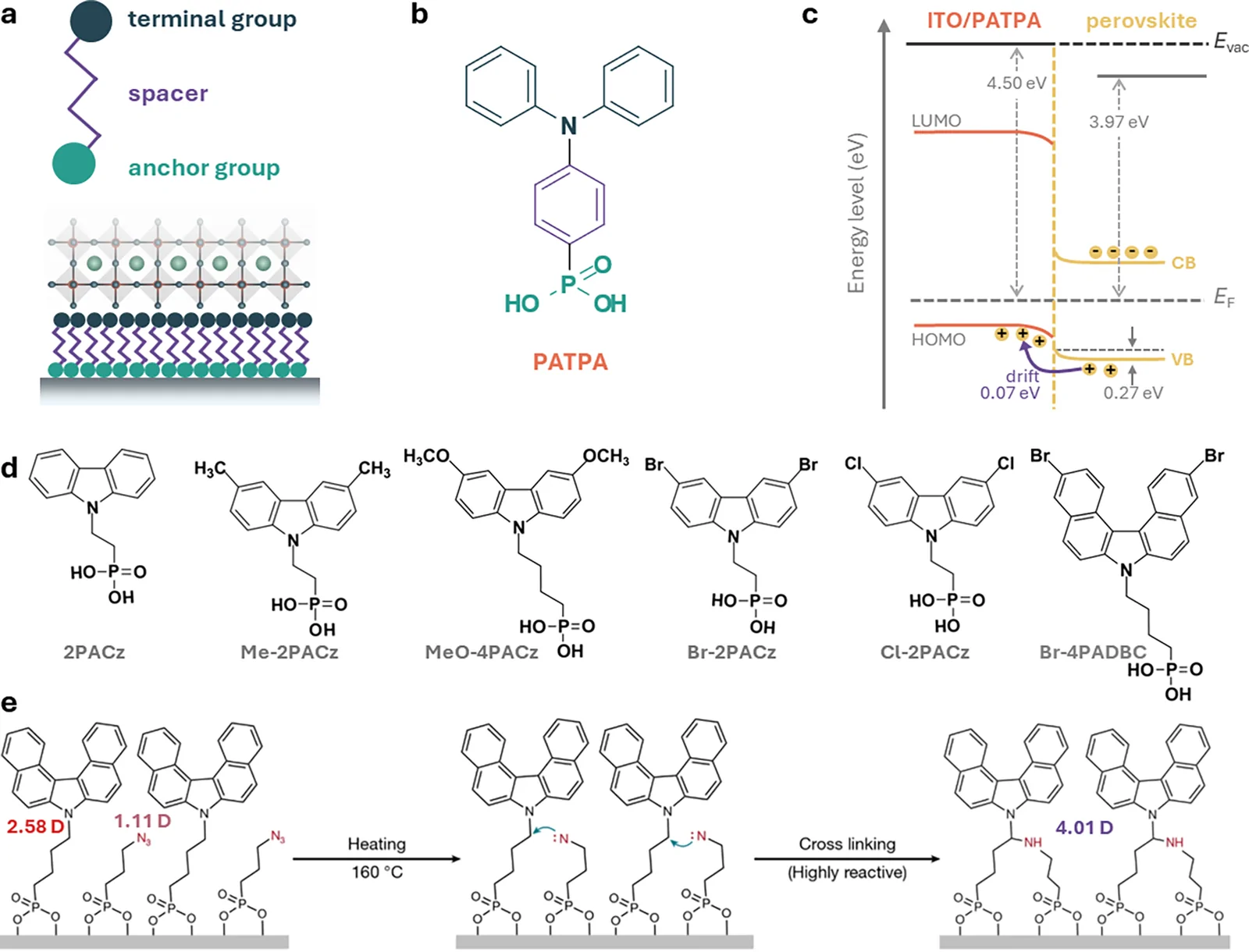
(a) Schematic illustration showing the general structure of a SAM and the interaction to form a close-packed layer. (b) Chemical structure of PATPA, combining a rigid spacer and a flexible terminal moiety. (c) Energy level diagram showing the band bending that occurs at the interface between the PATPA (hole transport layer) and the perovskite, which facilitates hole extraction by decreasing the energetic barrier for the majority charge carriers. (d) Molecular structures of the family of carbazole-based molecules used as SAMs. (e) Cross-linking reaction between the host SAM and the guest molecule. The values indicated in red, pink, and purple are the dipole moment of the SAM, the guest molecule, and the cross-linked SAM, respectively. (b) Adapted with permission from ref 325. Copyright 2017 John Wiley & Sons, Inc. Creative Commons Attribution 4.0 International License. (c, d) Adapted with permission from ref 320. Copyright 2025 Springer Nature. Creative Commons Attribution 4.0 International License. (e) Adapted with permission from ref 322. Copyright 2025 Springer Nature (f) Adapted with permission from ref 327. Copyright 2025 Springer Nature.

Regarding the anchor group, phosphonic acid (−PO­(OH)_2_) is considered the most promising due to its ability to anchor different substrates, forming either mono-, bi-, or tridentate P–O–M (where M is the metal atom on the substrate) binding modes depending on the substrate.^317,318^ It is generally assumed that phosphonic acid derivatives surpass silane-based SAMs due to weaker interactions with the substrates of the latter, and the ease with which individual molecules polymerize via neighboring −OH groups, complicating the formation of a well-defined packing and potentially leading to nonhomogeneous coverage of the substrate. However, the experimental reports are inconsistent, showing improvements in PCE for both anchoring groups.^319–321^ To strengthen the interaction between the SAM and the substrate, an alternative is to design a SAM with multiple anchoring groups.^322,323^ Moreover, substrate treatment approaches that form multiple −OH terminations, via acid or basic treatment, for instance, make the surface more hydrophilic and reactive, thereby strengthening the interaction between the anchor group and the substrate, minimizing the chance of SAM desorption.^319^

The terminal group in a SAM ultimately determines the surface energy of the layer, as it is typically the group that points outward from the substrate surface. Additionally, it influences the molecule’s dipole moment, orientation, and, importantly, its chemical interactions with the perovskite, which affect the crystal growth and quality while also can have a defect passivating ability.^324^ For example, the effect of the terminal group was investigated by Wang et al.^325^ They used several SAMs with a carboxylic acid as the anchor group and a phenyl ring as the spacer, and explored various terminal functional groups (−NH_2_, −OCH_3_, −H, −Cl, and −Br). The authors applied these molecules to modify the work function of NiO_*x*_ in inverted *p-i-n* PSCs, thereby affecting *V*_*OC*_. They found that electron-donating groups, such as −NH_2_, −OCH_3_, create a negative dipole on the NiO_*x*_, decreasing the work function of the oxide layer and lowering the *V*_*OC*_. Conversely, electron-withdrawing groups like −Cl and −Br generate a positive on NiO_*x*_, increasing its work function and raising *V*_*OC*_.

The spacer group affects charge selectivity and conductivity in the molecule and influences intermolecular interactions that facilitate self-assembly into a compact, ordered layer. Additionally, the spacer group influences the energetics of both the substrate and the perovskite. Yang et al. proposed a SAM comprising a phenyl ring, a rigid, conjugated spacer group, and a flexible terminal group (diphenylamine), anchored via a phosphonic acid moiety (Figure 29b).^320^ This design aimed to achieve a balance between efficient charge transport, enabled by the conjugated and rigid spacer, and the relief of interfacial stress, which is facilitated by the flexible terminal group that can adopt different conformations during packing. The authors observed that the resulting molecule exhibited a significantly larger dipole moment and a more continuous electron cloud than a similar SAM in which an ethyl group replaced the phenyl spacer. When deposited onto an ITO substrate and assembled into an inverted *p-i-n*-type PSC, the SAM deepened the Fermi level of the surface, thereby improving VBM alignment with the perovskite, which is beneficial for hole extraction and overall device performance (Figure 29c).

So far, one of the most widely used SAMs uses carbazole as a spacer and phosphonic acid as an anchoring group. Variants of this family of SAMs with different terminal groups or backbone substitutions are shown in Figure 29d, and have been used to fine-tune the dipole-induced Fermi level shift of ITO and to optimize interfacial energy level alignment. Among them, one of the best performing is the Br-4PADBC. Due to the presence of a dibenzocarbazole (DBC) core with two bromine atoms, a significantly larger dipole moment is obtained ascompared to MeO-2PACz.^326^ The larger dipole formed by a bilayer of these two SAMs results in a deeper work function of the ITO, providing better alignment with the perovskite valence band. Using a similar molecule but without the -Br substitution to the DCB unit, Jiang et al. introduced a cross-linkable strategy for SAMs (Figure 29e).^322^ This method uses a guest molecule (comprising a phosphonic acid, an alkyl linker, and an azide, named JJ24) in combination with a host SAM (4PADBC). During the annealing step of the SAM film, the azide of the guest molecule releases N_2_ (*g*), and the remaining nitrogen forms a covalent bond with 4PADBC. The formation of this bond suppresses conformational changes under stress, enhancing the rigidity of the SAM layer and improving surface coverage. Furthermore, the resulting cross-linked molecule exhibits a superior dipole moment compared to the pristine 4PADBC, thereby shifting the ITO work function toward lower energy, and enhancing hole extraction.

In the broader context of dipolar interface modification, π-conjugated polyelectrolytes (CPEs) can be regarded as an extension of SAMs, combining semiconducting backbones with ionic side chains that generate strong interfacial dipoles (Figure 30a). The delocalized π-system enables electronic coupling with adjacent charge-transport layers, while the ionic groups establish a dipole-induced potential step when adsorbed onto oxides or transparent conductive substrates. Compared with SAMs, CPEs form ultrathin polymeric interlayers that provide a uniform coverage, minimize interfacial voids, enhance surface wetting, and guide perovskite nucleation. As a result, CPEs can simultaneously reduce energetic mismatches at the interface and improve the structural quality of the perovskite film. In CPEs, the choice of the cation and anion is an important aspect. For example, Seo and Nguyen demonstrated how the charge state and the use of different anions in CPEs determine their electronic energy levels and interfacial dipole strength.^327^ By systematically examining a family of PFN-based CPEs (Figure 30a), they observed significant variations in HOMO energies, from 1.22 eV (PFN-Br) to 1.68 eV (PFN^+^Br^–^), 1.75 eV (PFN^+^BIm_4_^–^), and 1.15 eV (PFN-SO_3_^–^Na^+^), highlighting that ionic configuration is a dominant factor in setting polymer energetics. Furthermore, when interfaced with gold, cationic CPEs consistently lowered the electron injection barrier, whereas neutral or anionic CPEs lowered the hole injection barrier. This work established a mechanistic framework in which interfacial dipoles arise from the orientation and distribution of charged side chains, providing a direct, tunable route to modulate the vacuum level at semiconductor-metal contacts. Liu et al.^328^ examined the role of anchoring groups in CPEs by comparing CPEs functionalized with −NMe_3_^+^ (Me: methyl), −SO_3_^–^, and −NH_3_^+^ groups deposited on PEDOT:PSS and used this as a hole transport layer in inverted PSCs. It was found that all these groups can modulate the HOMO of the underlying HTL, reducing interfacial energy losses and significantly influencing perovskite nucleation. Among them, −NH_3_^+^ and −SO_3_^–^ derivatives provided the most favorable film growth and interface energetics.

**Figure 30:**
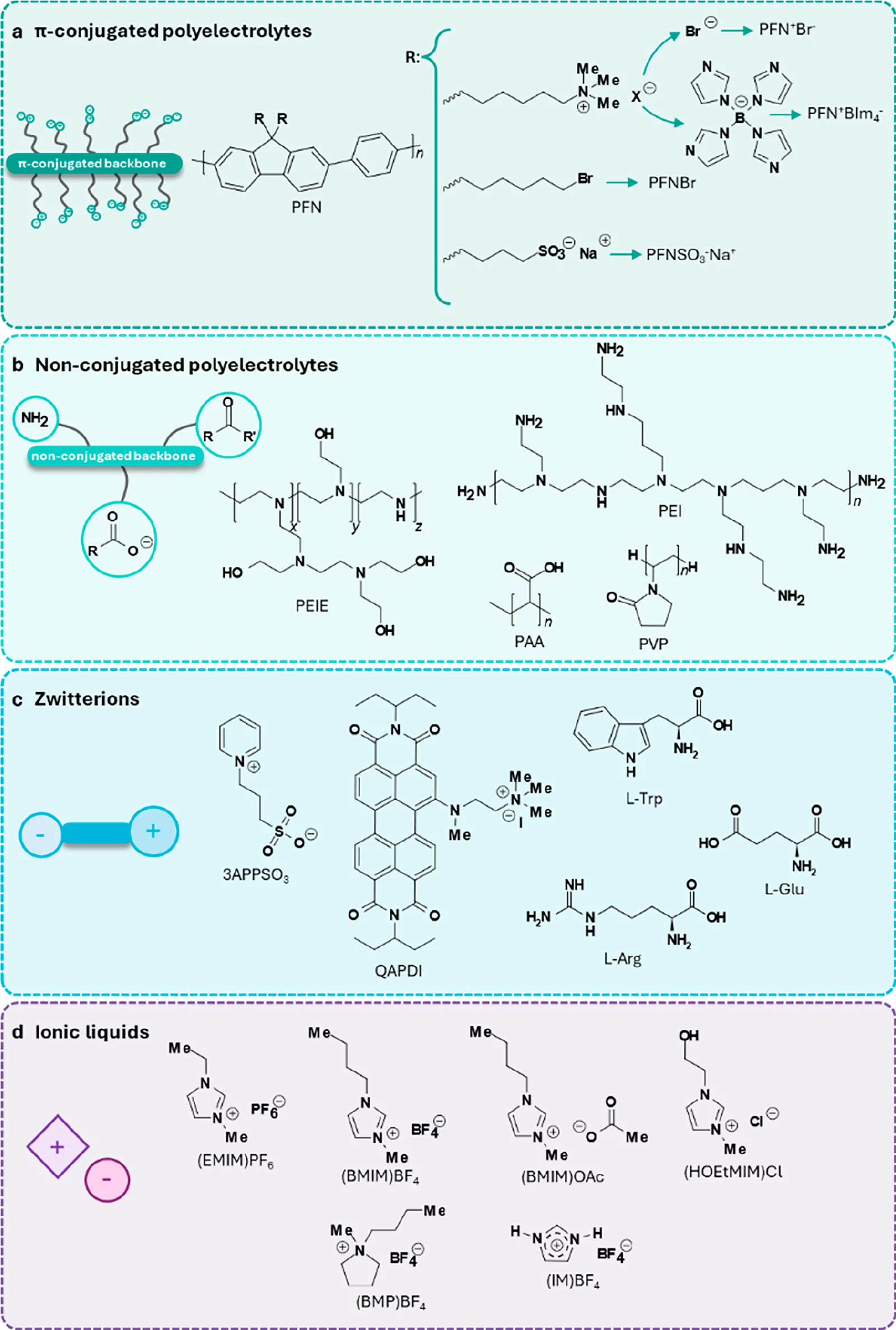
Different classes of molecules are used as a dipolar layer in perovskite solar cells: (a) π-conjugated polyelectrolytes, (b) nonconjugated polymers, (c) zwitterions, and (d) ionic liquids.

Another essential aspect of CPEs, in addition to tailoring interfacial energetics, is their ability to improve the quality of perovskite films. For example, the amphiphilic CPE PFN-Br, while forming an interlayer that shifts the perovskite Fermi level toward shallower energies, thereby promoting hole extraction, also increases the surface polarity, which improves perovskite wetting and crystallization.^329,330^ This molecule has been used to modify the surface energy of the poly­(triarylamine) (PTAA) used as hole transport material in inverted PSC, improving the wettability of the perovskite film.^331–333^ It is also possible to introduce functional groups to CPEs that can interact with defects in the perovskite. For example, the use of thiophene-based CPEs, bearing a carboxylic acid as the anchoring group, allows for the passivation of undercoordinated lead atoms at the perovskite interface, in addition to introducing interface dipoles that maximize quasi-Fermi level splitting.^334^ Similarly, Lyu et al.^335^ showed that a fluorinated conjugated polymer at the perovskite/HTL interface induces a strong local dipole through F–Pb interactions, leading to favorable band bending and improved hole extraction.

Nonconjugated polymers are another class of molecules employed to tune interfacial energetics in PSCs. Their dipole character typically arises from polar functional groups, such as amines, carbonyls, or carboxylates, that generate interfacial electric fields when adsorbed onto transport layers or electrode surfaces (Figure 30b). One of the earliest demonstrations of this strategy was reported by Zhou et al., who introduced poly­(vinylpyrrolidone) (PVP) as an ultrathin interlayer between the perovskite and PCBM in inverted *p-i-n* PSCs (Figure 30b).^336^ The authors observed a positive shift in surface potential, attributed to a permanent dipole moment of the PVP layer oriented toward the metallic contact. This interfacial dipole modified the vacuum-level alignment, enhancing electron extraction and improving device performance. Other examples of nonconjugated polymers include polyethylenimine (PEI) and its ethoxylated derivative (PEIE) are shown in Figure 30b. These polymers are reported to lower the work function of electron-transporting oxides, thereby facilitating electron extraction while suppressing interfacial recombination. Such polymers have been successfully applied across a wide range of electrode materials, including oxides, organic semiconductors, and carbon-based electrodes, in which their interfacial dipoles consistently lower or raise the local work function, thereby tuning the energy-level alignment.^337–346^ Poly­(acrylic acid) (PAA, Figure 30b) and its derivatives have also been employed, with the carboxylic acid anchoring to oxides, which enables interface modification and defect passivation.^347,348^ Nonconjugated polymers, despite lacking π-conjugation and therefore potentially preventing charge transport across the interlayers, can form ultrathin, uniform layers that mimic the function of molecular SAMs. It is crucial to control the interlayer thickness and devise strategies to mitigate the low conductivity of these materials. This can, for instance, rely on creating mixtures of the nonconjugated polymer and conjugated molecules.^349^

Zwitterionic molecules have also emerged as highly effective interfacial modifiers in PSCs. Because they contain both positive and negative functional groups within the same molecule, zwitterions can interact simultaneously with the perovskite surface and the ETL (Figure 30c).^350–352^ This dual affinity enables not only improved charge extraction but also the passivation of a wide range of interfacial defects, including Lewis acidic/basic sites and undercoordinated metal ions. At the SnO_2_/perovskite interface, zwitterions have been shown to promote more uniform SnO_2_ surface coverage by the perovskite solution and to facilitate the growth of high-quality perovskite films.^353^ Additionally, they can also modulate the ETL work function, enhance its conductivity, and reinforce electron selectivity, thereby suppressing interfacial backflow and reducing hysteresis. These combined effects result in improved energy-level alignment and enhanced device performance. For instance, Kim et al.^354^ employed 3-(1-pyridinio)-1-propanesulfonate (3APPSO_3_, Figure 30c) at the SnO_2_/perovskite interface, achieving more efficient charge-carrier extraction and reduced hysteresis in regular PSCs. Zwitterion interlayers have also proven effective in inverted architectures. Wang et al. introduced a perylene diimide-based zwitterion (QAPDI) as an electron-collecting interlayer between PCBM and the top metal electrode, thereby reducing interfacial energy loss and improving device performance.^355^ A key challenge associated with zwitterions is their tendency to aggregate, owing to strong electrostatic interactions between their internal positive and negative charges. Such aggregation can lead to morphological inhomogeneity, interfacial defects, and unwanted recombination pathways. A recent strategy to mitigate this issue involves reacting the zwitterion with lithium bis­(trifluoromethane)­sulfonimide (LiTFSI), in which Li^+^ ions disrupt intermolecular interactions and suppress aggregation, yielding improved interfacial quality and device efficiency.^354^ Together, these findings highlight the versatility of zwitterions as interfacial modifiers, capable of providing electrostatic tuning, defect passivation, and improved film morphology, while underscoring the importance of controlling their intermolecular interactions for optimal device performance.

Another interesting class of molecules used to tune the energetics of the PSC is ionic liquids (IL). These molecules are salts that remain liquid at or near room temperature. They typically comprise ammonium-based organic cations, counterbalanced by inorganic anions, such as halide, tetrafluoroborate, phosphate, and imide. Ionic liquids feature high polarity, tunable dipole moments, low volatility, and good interfacial wettability, making them attractive candidates for use in PSCs. Several studies have demonstrated the ability of ILs, including (EMIM)­PF_6_,^356^ (BMIM)­BF_4_,^357,358^ (HOEtMIM)­Cl,^359^ (BMP)­BF_4_,^360^ (IM)­BF_4_,^361^ (BMIM)­OAc,^362^ potassium tetrafluoroborate (Figure 30d),^363^ to improve the energy level alignment at critical PSC junctions. These molecules are reported to form a negative dipole moment, resulting in a downward shift in the *WF* of the underlying layer. From this observation, it is possible to conclude that the positively charged ions in the IL likely bind to the transparent conductive oxide through dangling bonds in the substrate.^360^ This interaction creates a negative dipole moment, which clarifies the role of IL in interfacing with the ETL. Interestingly, Li et al. utilized methylammonium acetate (MAAc) in ETL-free PSC.^364^ The researchers used MAAc as a solvent in the perovskite solution and found that this molecule physically adsorbs onto the ITO surface, lowering its work function and facilitating an *in situ* band bending that promotes electron extraction. This finding enabled them to create PSCs without requiring a dedicated ETL, resulting in a PCE of 21%, and thus simplifying the device fabrication process by eliminating one step.

To the best of our knowledge, there are fewer reports on the positive dipole induced by ILs. In one of the examples, Xu et al. explored alkylammonium (AM^+^) acetate ionic liquids with chain lengths of 4, 5, and 6 carbons at the interface between the perovskite and the HTL in regular *n-i-p* PSCs.^365^ By varying the size of the alkylammonium chain, the dipole moment of the AM^+^ cation increased from 9.17 D for butylammonium to 14.74 D for hexylammonium. Additionally, the longer alkyl chain facilitates stronger intermolecular van der Waals interactions and chemical coordination with the perovskite surface, which increases the binding energy. Moreover, the energy level offset between the VBM of the perovskite and the HOMO of the HTL decreases, helping to explain the improvement in PCE, which reached 25% with hexylammonium acetate.

It is crucial to note that, in addition to altering interfacial energetics, these interlayers are frequently reported to affect ion migration, a key factor for achieving long-term device stability. The suppression of ion migration is associated with the formation of a potential step at the interface, which impedes ionic drift. For example, Tang et al. demonstrated that a potential barrier of 0.6 eV at the perovskite surface can reduce ion migration within the device across various perovskite compositions.^366^ The authors overcame this potential barrier by utilizing a dipolar interlayer, which significantly impacts the drift and accumulation of halide ions when the device is exposed to light and an applied bias. In summary, dipolar interlayers can reduce ion migration when their dipole moments are aligned in opposition to the built-in electric field of the perovskite. This alignment helps minimize the drift component of ionic transport, although ion migration through diffusion within the perovskite can still occur and would need to be addressed through passivation in the perovskite’s bulk. To achieve effective ion confinement, it is essential to control the orientation, packing density, and magnitude of the dipoles. This control must create a sufficiently strong electrostatic barrier while avoiding excessive interfacial electric fields that could hinder electronic extraction or affect long-term stability.

It is worth mentioning that most of the shifts in energetics we have addressed above occur without forming chemical bonds. The strong physical adsorption of these molecules to the substrate can lead to a compact film. This not only contributes to a more stable interface but also helps establish a defined and oriented dipole at the interface. These characteristics are key aspects of SAMs, which feature anchor groups that strongly chemisorb to oxide substrates through interactions with surface −OH terminations. This strong molecule–substrate interaction minimizes desorption or reorientation events that could otherwise disrupt the interfacial dipole. To achieve a comparable level of interfacial stability with ILs and zwitterions, careful control of molecular packing and charge distribution within the molecules is generally required. These factors ultimately dictate the intermolecular interactions. It is important to note that for these molecules, the interaction with the substrate, whether a perovskite or a transparent conductive oxide, is typically driven by weaker physisorption rather than chemisorption, as is the case for SAMs containing phosphonic acids. Consequently, ionic liquids and zwitterions can be more susceptible to dipole reorganization or aggregation under thermal or bias stress. In this context, tuning the molecular structure by increasing molecular rigidity, adopting multidentate binding modes, or adding stronger binding units would help stabilize the interface dipoles.

### 5.3 Electrical Doping of CTLs

Charge-transport layers may constitute a performance bottleneck in perovskite solar cells if they are highly resistive or have low charge carrier mobility. This can constrain charge extraction, causing the charge accumulation at these interfaces, and leading to recombination losses. While the minimization of the layer thickness can be, and often is, a sufficient remedy, one must be careful to ensure a good film coverage, which becomes a serious challenge for thin films. Alternatively, doping is a well-known method for improving the mobility and the conductivity of semiconductors. The methods to control the doping of organic and inorganic semiconductors used as CTLs in PSCs have been widely established,^367–371^ and can therefore serve as an excellent tool to increase conductivity and/or carrier mobility.^372^

Moreover, doping of the CTLs offers a unique way to shift the Fermi level of the charge-selective layers without changing their CBM and VBM, thereby modifying the equilibrium band alignment at both the perovskite/CTL and CTL/electrode interfaces. This improves efficient charge transport to the electrode and can suppress the formation of Schottky barriers at the CTL/electrode interface. The resulting reduction in contact resistance directly lowers the series resistance of the device and enhances fill factor and operational stability. In this sense, doping CTLs is a tool for shaping the interfacial potential landscape and enforcing favorable boundary conditions for charge flow, while simultaneously increasing their conductivity.

#### 5.3.1 P-Doping of HTLs

P-type doping of hole-transport layers raises their Fermi level toward the VB edge (Figure 31a), increasing the hole density and reducing the energetic offset at the perovskite/HTL and HTL/electrode interfaces. This narrows interfacial depletion regions, suppresses Schottky barriers, and enables quasi-ohmic hole extraction, thereby lowering series resistance and mitigating interfacial recombination. Most importantly, however, the doping increases the film’s conductivity, reducing charge accumulation at the interface with the much more conductive perovskite.

**Figure 31:**
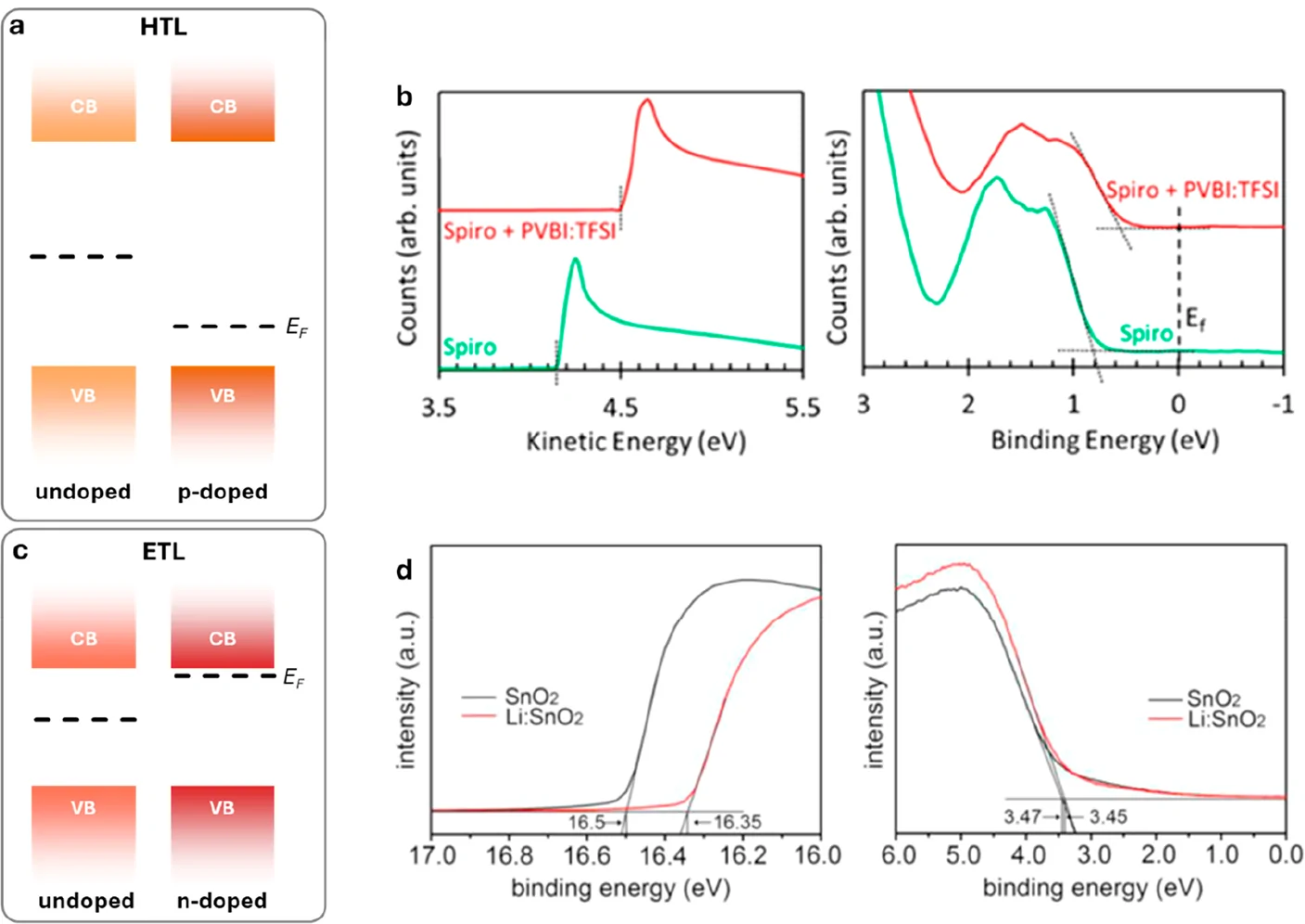
(a) Fermi-level shift toward the valence band upon the *p*-type doping. (b) UPS spectra of SpiroOMeTAD: undoped and doped with PVBI:TFSI, showing the increase of the *WF* and the increase of the energy with respect to *E_F_* upon doping. Reproduced with permission from ref 376. Copyright 2020 American Chemical Society. (c) Fermi-level shift toward the conduction band (CB) upon the *n*-type doping; (d) UPS spectra of undoped and Li-doped SnO_2_, demonstrating a decrease of the *WF* (scale inverted with respect to panel b) and the increase of the energy with respect to *E_F_*. Reproduced with permission from ref 391. Copyrights 2016 Elsevier Inc.

Even the ubiquitously used HTL in the *n-i-p* architecture, i.e., Spiro-OMeTAD, suffers from low conductivity and is always doped to reach high PCEs.^373,374^ While most common *p*-type dopants of this molecule are 4-*tert*-butylpyridine (tBP) and Li-TFSI,^374,375^ other interesting approaches have been proposed recently. For instance, Geffroy et al. demonstrated the *p*-type doping of Spiro-OMeTAD using poly­(1-butyl-3-vinylimidazolium bis­(trifluoromethylsulfonyl)­imide) (PVBI-TFSI) as dopant,^376^ resulting in the conductivity increase by 4 orders of magnitude. As concluded from the UPS spectra of the thin films (Figure 31b), the Fermi level of the HTL shifts toward the valence band upon the doping with either dopant. As a doping mechanism, the authors propose a proton transfer from PVBI^+^ to Spiro-OMeTAD, which is partially supported by the nuclear magnetic resonance (NMR), yet the unambiguous evidence remains to be demonstrated. Other recently investigated dopants of Spiro-OMETAD include the ionic radical Spiro-OMeTAD^2•+^(TFSI^–^)_2_, which provides hole polarons that increase conductivity and, together with TBMP^+^TFSI^–^, modulates the *WF*.^377^ In another work, Li^+^ was replaced by other metal anions. It was found that Ca­(TFSI)_2_ and Mg­(TFSI)_2_ promote enhanced oxidative doping, whereas NaTFSI predominantly induces interstitial doping within the bulk perovskite. In addition, KTFSI acts as a catalytic species, lowering the reaction energy barrier for the other dopants, thereby facilitating the more rapid formation of the Spiro-OMeTAD radical cation.^378^

Similarly, PTAA can be doped to increase conductivity and shift the Fermi level of the HTL.^379^ Alike Spiro-OMETAD, PTAA is often doped with tBP and Li-TFSI,^380,381^ with novel dopants including fluorinated iron­(III) porphine (Fe­(III)-PP) or nonionic PFB-TFSI.^380,382^ Often lacking unambiguous evidence of the doping effect from UPS and an increase in conductivity, the doping efficiency is commonly inferred from improved power conversion efficiency, possibly ignoring other effects, such as the reactions with the mobile ions from the perovskite layer under operation or surface defect passivation.

A mild doping effect of NiO_*x*_ can be obtained by the vacancy modulation by the introduction of other metal species, such as Fe, Sr, or Cs.^383–385^ An interesting strategy was proposed by NiO_*x*_ doping with fluorine, demonstrating an increase of the Ni^3+^/Ni^2+^ ratio, due to the formation of NiF and O–F species, leading to the modest increase of conductivity by 26%.^386^

#### 5.3.2 N-Doping of ETLs

N-type doping of the ETLs is a common approach to increase their conductivity. Additionally, *n*-type doping shifts ETL’s Fermi level toward the conduction-band edge (Figure 31c), increasing the electron density and minimizing the barrier for electron transfer at both the perovskite/ETL and ETL/electrode contacts. In addition to the increased conductivity, the resulting reduction in band bending and depletion width promotes efficient electron extraction and stabilizes interfacial energetics under operating conditions.

Metal oxides are a common choice of ETLs in the *n-i-p* architecture. For example, Vasilopoulou et al. showed a 7-fold increase in conductivity and a Fermi-level shift toward the conduction band as a result of TiO_2_ annealing in H_2_ or N_2_ atmospheres, pointing to interstitial H^+^ ions as donors.^387^ For TiO_2_ and SnO_2,_ a modest impact on the electrical properties of the oxide layer can be obtained by doping with metal ions. Here, the doping is achieved either by vacancy modulation or heterovalent substitution, but the exact doping mechanism is difficult to determine unambiguously.^300,388–390^ Small metal ions, such as Li^+^, tend to occupy interstitial sites and act as donors. For instance, Park et al. doped SnO_2_ with Li^+^ ions, resulting in a 2.5-fold increase in conductivity and a shift of the Fermi level toward the conduction band, demonstrating *n*-type doping (Figure 31d).^391^ Organic ETLs, including fullerenes, are typically doped with molecules with shallow LUMO levels, for example Bis-PCBM with DMC and PNDI-2T with N-DMBI.^392,393^

#### 5.3.3 Limitations

The electrostatic modification at the perovskite/CTL interface induced by CTL doping may perturb the dynamic landscape of mobile ionic species and electrically active surface defects. Consequently, an energetic shift measured after CTL doping may partially originate from secondary processes within the perovskite, including (i) redistribution of halide vacancies,^394^ (ii) formation of interfacial dipoles,^395^ or (iii) chemical interactions between dopant species and the perovskite surface.^396^ In this sense, the doped CTL does not merely act as a passive electronic contact but can actively reshape the interfacial electrostatics of an ionic semiconductor. The resulting device behavior reflects a coupled electronic–ionic response, rather than a simple semiconductor junction defined by static band offsets.

For instance, Li-TFSI in Spiro-OMeTAD indeed increases the conductivity and the hole density in the HTL, yet Li^+^ ions are mobile and can migrate toward the perovskite/HTL interface, where they modify interfacial energetics and defect populations.^397^ Similarly, molecular *n*-dopants in fullerene-based materials often form localized charge-transfer complexes at or near the interface; hence, the doping effect can be hard to disentangle from dipole formation or surface passivation.^398,399^ In metal-oxide transport layers such as TiO_2_, NiO_*x*_, or SnO_2_, doping is commonly achieved through oxygen vacancies, alkali ions, or aliovalent substitution.^394,400^ While these modifications achieve reduced contact resistance and improved extraction, it cannot be ruled out that they arise from an interfacial interaction between the CTL dopant and the perovskite, rather than from homogeneous volumetric modification of the transport layers. Therefore, the band alignment at the perovskite interface may reflect not only the changes of the transport properties of the CTLs, but also ion redistribution,^394^ interfacial charge transfer, or dipole formation.^395^ Consequently, improvements attributed to “doping-induced energetic alignment” cannot always be unambiguously separated from perovskite-side electrostatic reconfiguration. Doping of transport layers should thus be viewed less as a classical semiconductor strategy and more as a tool for interfacial Fermi-level control and contact optimization. While numerous studies report efficiency gains upon CTL doping, only a subset directly demonstrates the intended energetic realignment, and even fewer address its evolution under operating conditions. Consequently, the principal advantage of CTL doping in PSCs lies in its ability to suppress contact barriers and series resistance by their increased conductivity, rather than in modifying bulk transport.

### 5.4 Interface Passivation

Although inorganic materials, such as potassium iodide (KI),^401,402^ potassium thiocyanide (KSCN),^403^ cesium iodide (CsI),^404^ and cesium fluoride (CsF),^405^ have been used to passivate perovskite interfaces, organic molecules remain the most investigated ones. The attractiveness of organic molecules stems from their ability to be tuned to interact with a specific type of defect.^406,407^ This can be achieved by chemically manipulating the molecular structure by incorporating desired functional groups, such as amines, ammonium, carboxylic acids, ketones, and alcohols, among others. The presence of these functional groups not only helps passivate defects at the interfaces but may also inadvertently generate dipole interlayers at the perovskite interfaces. Disentangling the effects of passivation and the changes in work function induced by these molecules can be challenging. In principle, any organic molecule with a nonzero intrinsic dipole moment can lead to the formation of a dipole interlayer. Although passivation and dipole formation effects occur simultaneously, it is possible to emphasize the passivation effect over dipole formation by using molecules with a low dipole moment or by employing sufficiently thin interlayers.

Passivating interfaces is key to achieving high performance and stability in PSCs. Passivation typically reduces trap density at interfaces, thereby decreasing nonradiative recombination and enhancing device performance. Additionally, most molecules used to passivate defects at interfaces are hydrophobic, helping prevent moisture and oxygen from permeating into the perovskite. The defects present at the interfaces of the perovskite with the CSL are regarded as the primary energy loss mechanism in various perovskite compositions.^408,409^ Figure 32a presents the estimated *V*_*OC*_ loss as a function of bandgap. Ideally, we would expect *V*_*OC*_ to increase with a higher bandgap. However, since bulk recombination decreases as the bandgap increases, the share of the interface recombination losses tends to rise significantly for wide bandgap perovskites. This highlights the need for interface passivation to reduce the density of trap states at the interfaces. Irrespective of the composition, these defects can arise from various sources, often stemming from the imperfect termination of the lead halide octahedron layers, grain boundaries, the formation of undercoordinated species, degradation products, and chemical reactions between the perovskite and adjacent layers. These defects trap charge carriers, leading to the formation of an interface charge layer.^410^ This layer screens the internal electric field, which mitigates the diffusion of charge carriers toward the interface.^410^ The presence of surface defects causes Fermi-level pinning at the perovskite interfaces, significantly reducing the internal quasi-Fermi-level splitting in the perovskite layer and, consequently, the *V*_*OC*_. Furthermore, the presence of defects increases nonradiative recombination in the device.^411^ Because there are many causes of performance loss when defects are present at interfaces, the enhancement in *V*_*OC*_ upon interface passivation cannot be interpreted solely as a consequence of improved energy-level alignment. In many cases, spectroscopic measurements reveal little or no shift in the energy levels after passivation, while substantial gains in QFLS and *V*_*OC*_ are observed. In this context, the passivation strategies discussed here improve device efficiency not by aligning energy levels, but by suppressing defect-assisted recombination, thereby decreasing internal QFLS.

**Figure 32:**
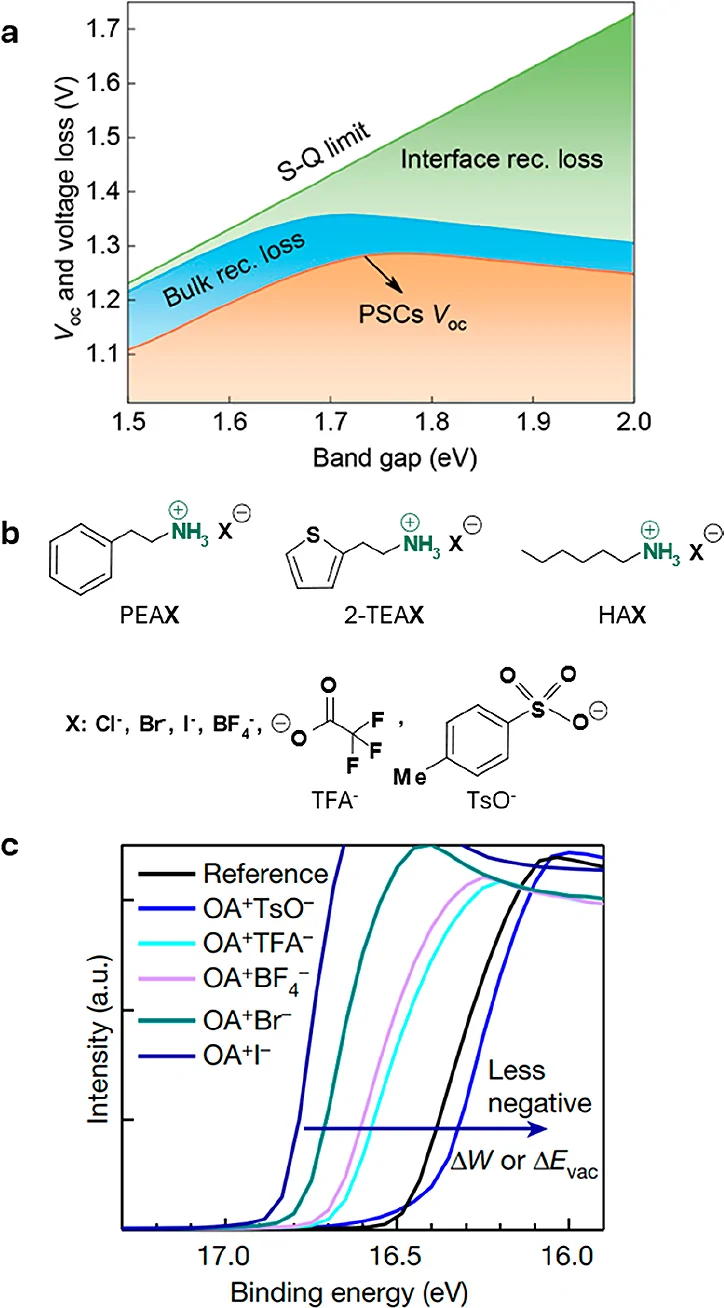
(a) Contributions of interface and bulk recombination losses to the voltage loss and *V*_*OC*_ of a perovskite solar cell with different bandgaps in the absorber. The S-Q limit represents the Shockley–Queisser limit. (b) Chemical structure of various ammonium-based cations and different anions (X) used for passivation of the interlayer in PSCs. PEA: phenylethylammonium. 2-TEA: 2-thiophenethylammonium. HA: hexylammonium. TFA: trifluoroacetate. TsO: tosylate. (c) Ultraviolet photoelectron spectroscopy secondary electron cut-offs of octylammonium (OA) treated films with varied halides. (a) Adapted with permission from ref 408. Copyright 2021 John Wiley & Sons, Inc. (c) Adapted with permission from ref 233. Copyright 2022 Springer Nature.

One of the most common ways to visualize and execute defect passivation in PSC is by observing the Lewis acid and base character of the defects and the corresponding interlayers. Lewis base groups, such as amines, pyridines, thiophenes, carbonyls, or phosphine oxide groups, facilitate the passivation of positively charged defects, including uncoordinated Pb^2+^ atoms. Conversely, acidic moieties, including hydrogen bond donors, such as ammonium (R-NH_3_^+^), and Lewis acids, such as boron trifluoride (BF_3_) or sulfur trioxide (SO_3_), facilitate the passivation of negatively charged defects, e.g., halide vacancies. Zwitterions are characterized by the presence of both positive and negative charges within a single molecule (Figure 30c). While these frequently manifest as Lewis acid–base pairs, they may also consist of ionic moieties that do not function as Lewis centers. In this sense, zwitterions are suitable candidates for passivating various types of defects.^412–414^ The effects of this dual-passivation were explored by Wang et al.^412^ They compare the passivating effects of l-tryptophan (l-Trp), which combines an ammonium, a carboxyl, and an aromatic functionality, with l-glutamic acid (l-Glu), characterized by multiple carboxyl groups, and l-arginine (l-Arg), which features a strongly basic guanidinium group (Figure 30c). These molecules were then applied to the NiO_*x*_ surface, and it was verified that l-Trp yields the best passivation effect. The carboxyl group binds to oxygen vacancies in the NiO_*x*_ surface. In contrast, the ammonium group interacts with halide atoms in the perovskite structure and also passivates cationic vacancies, such as *V*_A_ and *V*_Pb_. In the previous section, we discussed how zwitterions can also function as a dipolar layer. Indeed, the authors have identified that, in addition to the passivation effect, Trp induces a dipolar layer on NiO_*x*_, resulting in a 0.2 eV shift in the *E_F_* toward lower energy, indicating *p*-type surface doping. The net result of the Tpr surface treatment is an enhancement in device performance, primarily by increasing *V*_*OC*_ and *FF*, suggesting a reduction in charge-carrier nonradiative recombination at the interfaces.

In the context of interlayer passivation, ammonium-based organic molecules are among the most commonly used (Figure 32b). These molecules can have either an aliphatic core (such as butylammonium or hexylammonium) or an aromatic core (like phenylethylammonium, PEA^+^, or thiophenethylammonium, TEA^+^). They can be applied at the buried or top interface, as well as within the bulk of the perovskite material. Some of these molecules are reported to shift the surface Fermi level to either *p*-type or *n*-type.^415–418^ For example, Gharibzadeh et al. used PEACl to passivate both the bulk perovskite and the top interface of *p-i-n* PSCs.^419^ The authors confirmed the formation of a thin 2D perovskite layer, represented as (PEA)_2_(Cs_1–*x*_FA_*x*_)_*n*−1_Pb_*n*_(I_1–*y*_Cl_*y*_)_3*n*+1_, at the grain boundaries and surface. This modification reduces the trapping density and increases the charge carrier lifetime. Additionally, the authors identified that the formation of the 2D perovskite at the GB increases the QFLS by approximately 40 meV. At the same time, surface passivation created a dipole layer that blocked holes at the ETL/perovskite interface, resulting in an additional increase in QFLS of approximately 20 meV. Interestingly, the species used as a counteranion in ammonium-based salts was found to influence the surface work function.^264,420^ Tan et al. demonstrated that the substitution of iodide with anions of lower Lewis basicity alters the binding affinity to the metal center, due to the negative charge delocalization across the molecular framework. Consequently, bromide, tetrafluoroborate (BF_4_^–^), trifluoroacetate (TFA^–^), or tosylate (TsO^–^) in the ammonium-based salt helps suppress a negative work function variation after interface passivation (Figure 4c).^233^ The authors noted that this negative shift, which makes the surface more *n*-type, creates an additional potential barrier at the interface with the Spiro-OMeTAD used as HTL, causing charge accumulation that accelerates ion migration and impacts stability.

Defect passivation and changes in energy levels do not necessarily occur simultaneously, or at least do not produce detectable changes. In particular, a sufficiently thin passivation layer can effectively reduce the density of interfacial defect states, but it is insufficient to induce a measurable shift in the surface work function or to significantly increase the QFLS. Nevertheless, even under these conditions, a beneficial impact on device performance can still be observed. For example, it was demonstrated that using a very diluted solution of phenylethylammonium iodide (PEAI) derivatives to passivate the buried interface and bulk perovskite film in inverted *p-i-n P*SCs does not alter the interfacial energetics, even though device efficiency increases by over 3%.^421^ This improvement arises because interfacial defect states act as efficient charge-carrier trapping and recombination centers. In this sense, their passivation reduces nonradiative recombination losses, thereby improving charge extraction and transport without requiring a substantial modification of the nominal energetic alignment.

It is important to note that passivating molecules or polymers used at the interfaces often exhibit insulating or low-conductivity character. Even low-dimensional perovskites can hinder charge extraction. While the presence of such interlayers often suppresses interfacial recombination, excessive thickness of a low-mobility interlayer can introduce transport limitations.^130^ This may lead to quasi-Fermi-level bending at the interface contact, resulting in a significant mismatch between the QFLS and the *V*_*OC*_. An interesting strategy to address this issue was proposed by Peng et al.^422^ They used a thick (∼100 nm) Al_2_O_3_ dielectric layer with random nanoscale openings on top of the PTAA hole transport layer in *p-i-n* PSCs. By creating such random openings, the charge carrier can freely cross the perovskite to the HTL, while the Al_2_O_3_ islands reduce defect-assisted recombination. This strategy increased the *V*_*OC*_ from an average of 1.14 V (control) to ∼ 1.20 eV (Al_2_O_3_-passivated), corresponding to 94% of the Shockley-Queisser limit for a 1.55 eV solar cell.

In summary, the improvements in device efficiency obtained through interface passivation are fundamentally different from those achieved through energetic realignment. While interlayers may occasionally induce changes in dipoles or the work function, the primary mechanism, especially with sufficiently thin interlayers, is to suppress defect-assisted nonradiative recombination. Reducing interfacial trap states typically decreases Fermi-level pinning at the interface, enabling the absorber’s quasi-Fermi level to reach its full radiative limit. Given this context, it is reasonable to expect significant performance gains even when no measurable changes in energy levels are observed. This observation is particularly relevant in perovskite-based technologies, as it shows that predicting performance solely on the basis of band alignment criteria can be inadequate for predicting device behavior. This is in stark contrast to organic solar cells, where optimal band alignment, specifically having the HOMO of the HTL higher in energy than that of the absorber, and the LUMO of the ETL lower in energy than that of the absorber, is a crucial factor for determining performance. In the context of interlayer passivation in perovskite devices, it appears that nearly any organic molecule added to the interface can, to varying degrees, passivate interfacial traps. The rich defect chemistry at the interface allows it to interact with almost any organic group. Nevertheless, optimizing interlayer deposition conditions is essential. Factors such as solvent choice, annealing temperature, and concentration play a critical role in this process. Ideally, an effective interlayer design should strike a balance between chemical passivation and charge transport, suppressing defects without compromising charge extraction.

### 5.5 Ionic Management

The redistribution of mobile ions under bias or illumination influences the evolution of the built-in field, interfacial band bending, and ultimately the time-dependent performance of perovskite solar cells.^423^ The impact of mobile ions on the energetic landscape within the perovskite layer was introduced in Section 3.1.1, and the impact of the device’s energetic alignment on ionic movement was outlined in Section 3.3. However, here we focus on how ions interact with adjacent layers, typically charge-transporting materials, and on methods to suppress ionic motion, first within the perovskite film and second throughout the device.

#### 5.5.1 Impact of Mobile Ions on Device Performance and Longevity

Thiesbrummel et al. proposed a model describing the evolution of ionic movement in response to illumination-induced aging of the device based on current-density versus voltage (*J–V*) scans of perovskite solar cells of different formulations at various scan rates.^184^ Varying the scan rate is a good strategy for tracking ion migration in the system, as it allows for separating fast electronic responses from slow ionic processes in the device, and thus accounting for the ion migration contribution to hysteresis. It was found that prolonged illumination triggers the formation of ion/ion vacancy pairs, which are considered to be distributed homogeneously within the bulk of the perovskite film. At steady-state operating conditions with slow scanning rates (e.g., 0.125 V s^–1^), the ions move in the electric field and accumulate at both interfaces of the perovskite (Figure 33a). This layer of accumulated ions screens the electrons, leading to a decrease in PCE, mainly due to a drop in *J*_*SC*_ (Figure 33b,c). On the contrary, in the ion-freeze regime at a very high scan rate (e.g., 2500 V s^–1^), this effect is visibly suppressed, with negligible decreases in PCE and light-induced degradation when measured at fast scan speeds (Figure 33d). Moreover, the ion accumulation and screening were associated with the mismatch between the quasi-Fermi level splitting and the *V*_*OC*_. This mechanism suggests that the long-term stability of PSCs might be inherently linked to the formation and migration of mobile ions.

**Figure 33:**
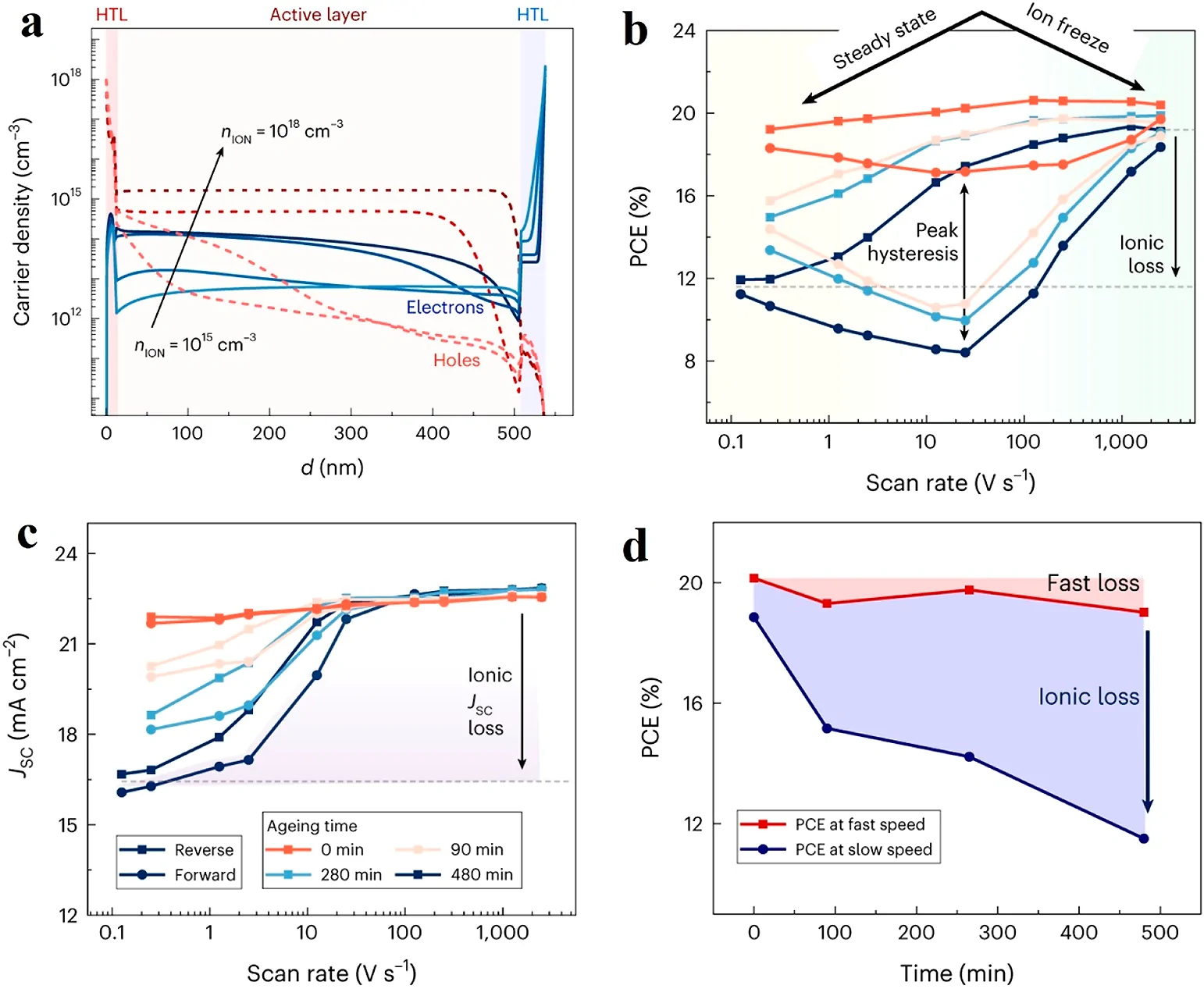
(a) Simulated electron (solid blue) and hole (dashed red) density profiles across the device for ion concentrations ranging from 1 × 10^15^ to 1 × 10^18^ cm^–3^, indicated from light to dark along the arrow. (b) PCE and (c) *J*_*SC*_ extracted from *J*–*V* measurements acquired at varying scan rates in reverse (squares) and forward (circles) directions for Cs_0.05_(FA_0.83_MA_0.17_)_0.95_Pb­(I_0.83_Br_0.17_)_3_ perovskite solar cells subjected to different aging durations. (d) Evolution of the absolute PCE at fast and slow measurement time scales as a function of aging time. The shaded regions highlight the performance deficit associated with ion migration: pronounced efficiency losses at slow scan speeds (blue) and substantially reduced losses at fast scan speeds (red). Reproduced with permission from ref 184. Creative Commons CC BY license. Springer Nature.

Furthermore, a substantial body of literature suggests that mobile ions can interact with both organic and inorganic CTLs. For example, Weber et al. reveal that the ionic charges created by mobile *I* ^*–*^ and immobile *V*_*I*_ pairs under bias appear at both interfaces of the perovskite film. Negative ionic charge is stabilized at the electron-collecting electrode (SnO_2_/MHP), probably due to complexation or binding reactions, while at the hole-collecting interface (MHP/Spiro-OMeTAD), iodide can be chemically trapped, forming longer-lived interfacial species,^182^ which causes a potential drop at this interface.^303^ The link between mobile ions and Spiro-OMeTAD to device hysteresis was widely reported in the literature.^36,424–427^ The screening potential forms within milliseconds of field application, while its relaxation is 40 times slower, indicating strong stabilization of interfacial ionic charge and causing band bending at these interfaces.^182^ The asymmetric relaxation time is a direct cause of hysteresis in PSC. A similar conclusion is proposed by Bergman et al., who explicitly indicate that mobile ions and electronic trap states at the interfaces are the root causes of the hysteresis.^302^ As a result, the interaction of mobile ions with CTLs can lead to a loss of charge selectivity due to changes in the electrode work function, oxidation reactions, corrosion, and, eventually, the formation of insulating layers via chemical reactions.

It is noteworthy that experimentally, ion migration is complex to distinguish from defect passivation and band-level alignment effects, and many studies consider all of these mechanisms in parallel. Mobile ions correspond to mobile defects, which can be electrically active and modify the bulk or surface electronic properties of perovskite devices. Defect formation, for example, under illumination, can therefore generate the same species that contribute to ion migration.

#### 5.5.2 Strategies to Suppress the Ionic Movement

Approaches for managing mobile ions have emerged as the understanding of their impact on the efficiency and stability of PSCs has deepened. They involve controlling the perovskite composition and microstructure, as well as strategies to prevent the intrusion of mobile species into adjacent layers.

##### 5.5.2.1 Engineering of the Perovskite Composition and Microstructure

##### 5.5.2.1.1 Halide Engineering

Compositional engineering emerged as one of the first approaches to suppress the ionic movement.^428–430^ For example, replacing I^–^ with more electronegative halides strengthens the ionic bonding in the lattice, increasing migration barriers. Another approach is the use of halide-containing additives. For example, fluoride-based additives are particularly effective, as F^–^ forms strong ionic and hydrogen bonds with Pb^2+^ and MA^+^/FA^+^, which correlate with higher vacancy formation energies and reduced ion migration.^431,432^ As a result, NaF-treated films exhibit more uniform surface potential distributions. Similarly, chloride incorporation raises the energetic barrier for halide motion and mitigates phase segregation.^428,430^ Triple-halide (I–Br–Cl) perovskites further demonstrate enhanced phase stability through Cl incorporation into the lattice,^429^ and later studies suggest that pseudohalides such as SCN^–^, BF_4_^–^, and PF_6_^–^ may offer similar benefits,^433–436^ though their mechanisms remain less well understood.

##### 5.5.2.1.2 Low-Dimensional Perovskites

Low-dimensional perovskites, both 1D and 2D, are reported to effectively suppress ion migration in 2D (1D)/3D perovskites because the bulky organic ligands used to produce this heterostructure can strongly anchor to surfaces and grain boundaries, blocking fast halide-diffusion pathways.^437–439^ For example, a study has shown that bipyridine-based 1D structures can form ion and/or hydrogen bonds with I^–^ or A-site ions in the 3D perovskite, thereby increasing the migration activation energy, restricting I^–^ transport at GBs, and enhancing moisture stability.^440^ In addition, 2D layered phases such as PEA_2_PbI_4_, in addition to passivate GBs, improve band alignment, and greatly enhance operational stability,^441–443^ effectively hindering ion migration due to the arylammonium cations (e.g., PEA^+^).^443^ *In-situ* PL imaging confirms much lower ion mobility in 2D/3D stacks compared to pristine 3D films.^444^ Time-of-flight secondary ion mass spectrometry (TOF-SIMS) further shows that iodide diffusion under thermal stress is suppressed by more than an order of magnitude when PEA-based 2D layers cap 3D perovskites.^445^ DFT and nudged-elastic-band (NEB) calculations reveal that I-vacancy formation energies and migration barriers are substantially higher in 2D perovskites (e.g., 0.67 eV vs ∼ 0.3 eV in MAPbI_3_), and mixed PEA_2_PbI_4_-MAPbI_3_ systems inherit this inhibited vacancy mobility.^446^ Additionally, similar approaches using PEAI as a precursor for the 2D perovskite,^445^ and alternative cations, such as 2-TEAI (2-thiophenethylammonium iodide) or multifunctional thiophene-based ammonium cations, can physically impede halide transport and enhance long-term stability.^447,448^ Other cations, such as butylammonium (BA^+^) halides, are also demonstrated to have a similar effect due to the formation of 2D/3D structures that prevent MA/I_2_ volatilization,^449^ and/or form a passivation layers that reduce defects and suppress ion migration.^450^ In addition to low-dimensional/3D structures, quasi-2D perovskite-based devices likewise display minimal temperature- or sweep-rate-dependent hysteresis, consistent with reduced vacancy motion, supported by higher formation energies for *V*_*I*_ and *V*_*MA*_ in 2D lattices.^441^ Low-frequency impedance analyses also show reduced ionic contributions, and additives, such as guanidinium, further hinder I^–^ migration in mixed-dimensional systems.^451^ These results demonstrate that dimensional engineering provides a robust pathway to inhibit halide migration without sacrificing device performance, as widely summarized across the 2D/3D heterojunction literature. Overall, extensive studies confirm that 2D and 2D/3D perovskites are intrinsically more stable against ion migration due to intercalated spacer ligands, strong van der Waals interactions, and out-of-plane ionic barriers.

##### 5.5.2.1.3 Microstructure of the Film

The impact of grain size on ion migration and film stability has been a long-established consensus. Grain boundaries act as sites of defects, facilitating ionic migration under illumination or bias, thereby decreasing long-term and operational stability.^452^ Therefore, increasing perovskite grain size to reduce GB density is a common approach to mitigate ion migration.

For example, it was suggested that seed-assisted growth with Cs^+^ nucleation yields much larger grains, fewer traps, and significantly improved device stability,^453–455^ while substituting PEDOT:PSS with PTAA as hole transport layer, or applying similar growth control, increases grain size (up to ∼ 5 times in MAPbBr_*x*_I_3–*x*_), suppresses phase segregation, and reduces trap density.^454^ A DFT and machine learning molecular dynamics (MLMD) study correlates lattice stability and the associated potential for ion migration with compressive strain, proposing the incorporation of B-site substitutions as a possible remedy.^456^ As the reliable B-site doping for the microstructure improvement poses an inherent challenge, a more practical approach toward this end was proposed by Singh et al., where a triple cation perovskite is deposited on the HTLs of different roughness, namely a smooth PTAA and on the MeO-2PACz, which, as a quasi-monolayer, mimics the roughness of the ITO.^457^ Surprisingly, it was found that smooth PTAA promoted compositional stratification, preferentially binding to MA^+^-rich perovskite and thereby impeding the formation of the defect-poor mixed-cation perovskite throughout the film, promoting ion migration and decreasing operational stability. In contrast, the textured buried interface led to better cation mixing, ensuring a homogeneous composition of the film.

The literature presents a cohesive picture in which low defect density results in less pronounced ionic migration. Consequently, many strategies aimed at suppressing ionic migration focus on improving the microstructure of the active layer.

##### 5.5.2.1.4 Inorganic Halide Salt Additives

Other strategies to control ion migration propose the introduction of halide salts as additives, especially KI or NaI, aiming to neutralize halide vacancies by introducing an excess of halogen ions^458^ or by raising the migration barrier by occupying interstitial positions with the small anion.^459^ DFT and device studies show that small alkali cations preferentially occupy size-matched interstitial sites, thereby significantly increasing the activation energy for halide migration. For example, K^+^ interstitials raise diffusion barriers from 0.11 to 0.59 eV, thereby blocking I^–^ motion.^460^ Surface measurements similarly indicate that low K^+^ loadings reduce surface ion mobility.^461^ According to first-principles calculations, interstitial Cs^+^ incorporation could provide a complementary effect, slowing photo-oxidation, increasing the activation energy of ion motion, and introducing an additional barrier along the *V*_*I*_ migration path.^461,462^ Beyond monovalent species, higher-valence interstitial cations such as Nd^3+^ interact more strongly with negatively charged intrinsic defects, yielding even greater suppression of defect migration and dramatically improved PL stability under illumination.^463^ Overall, these studies suggest a picture in which alkali (and alkaline-earth/lanthanide) cations could occupy interstitial or defect-associated sites, strengthen local bonding, and obstruct fast halide-migration channels, thereby stabilizing the perovskite lattice and improving device operational stability.

##### 5.5.2.1.5 Organic Additives

Since ion migration is mainly dominant at grain boundaries, many studies focus on passivating GB defects to suppress ionic motion. Chemical passivators such as methimazole (MMI) strongly reduce I^–^ diffusion, as shown by depth-dependent EDS where I and Ag signals are nearly absent at the HTL side after MMI treatment,^464^ while polymeric passivation using PCE10, a π-conjugated donor–acceptor copolymer composed of fused thiophene- and thienothiophene-based units, similarly lowers capacitance by an order of magnitude after aging, indicating restrained ion migration.^465^ Molecular interactions can also immobilize mobile species through surface defect passivation^466^ or by cation−π binding with rubrene (∼1.5 eV), which effectively pins A-site cations, thereby eliminating their diffusion, as demonstrated by TOF-SIMS.^467^ Likewise, interfacial engineering through carbon-based layers suppresses vacancy-driven I^–^ migration. For example, graphene-PCBM coatings mitigate thermal decomposition into PbI_2_ and volatile MA/HI, preserving absorption intensity during aging.^468^

##### 5.5.2.2 CTL Modification

##### 5.5.2.2.1 Metal Oxide-Based ETLs

Metal-oxide ETLs (such as SnO_2_, TiO_2_, ZnO) offer high mobility and good band alignment but can catalyze perovskite degradation and facilitate ion migration if unmodified.^469–472^ Nevertheless, surface engineering mitigates this. For example, SnO_2_ modified with red-emissive carbon quantum dots suppresses I^–^ migration and nonradiative losses, improving both efficiency and stability,^473^ while SnO_2_ treated with heparin potassium reduces interface defects and retains 97% PCE after 1000 h of operation without UV filtering.^474^ Additionally, KI and KCl post-treatment of the SnO_2_ layer is demonstrated to minimize the oxygen vacancies and react with hydroxyl groups at the surface through the formation of K–OH bonds, while the halide forms Sn-X-Pb bonds that minimize ion migration.^475^ This strategy helps obtain *n-i-p* PSC with minimal hysteresis and high efficiencies.^476,477^ In early studies, aluminum-doped zinc oxide was demonstrated to improve ambient stability, with the mechanism ascribed to the suppression of ion migration.^478^ Numerous other SnO_2_/TiO_2_/ZnO modifications likewise aim to passivate defects and inhibit ionic drift.^474,479,480^

##### 5.5.2.2.2 Fullerene-Based ETLs

Fullerene-based CTLs, such as PCBM or C_60_, are known to be more resistant to the incorporation of halides.^481,482^ This effect is often attributed to the formation of iodide-fullerene complex or generally ascribed to defect passivation. For example, it was shown that replacing TiO_2_ with C_60_/SAM in a standard *n-i-p* PSC results in nearly complete elimination of hysteresis.^303^ However, the limited solubility of fullerene-based materials can sometimes lead to nonuniform films and weak barrier properties, allowing ion migration and rapid degradation under illumination. Additive engineering improves PCBM’s barrier function, for example, poly­(ethylene-*co*-vinyl acetate) (EVA) forms a cross-linked PCBM:EVA network that enhances hydrophobicity, molecular ordering, and ion blocking, as confirmed by TOF-SIMS, enabling devices to retain 80–90% of their efficiency over 1500 h.^483^ *N*-doped graphene/PCBM composites (CQDs/G-PCBM) similarly control carrier and ionic transport, improving the device stability.^468^ Alternatively, fullerene derivatives such as fluorinated 5F-PCBP create strong H-bonding with MA^+^ at the interface, maintaining a stable ion profile and ∼ 70% PCE after 1000 h at 65 °C, unlike PCBM.^484^ Chelating FP-C8 yields tighter molecular packing and inhibits ion migration, giving 96% PCE retention over 1200 h.^485^ Alternatives to address the solubility issue of fullerene derivatives can involve vacuum-assisted thermal evaporation methods or chemical functionalization. Furthermore, nonfullerene ETLs such as PDTzTI also suppress ion migration by strongly coordinating surface Pb^2+^, reducing defects, and improving stability.^486^

##### 5.5.2.2.3 Sprio-OMeTAD

Spiro-OMeTAD is one of the most widely used hole-transport materials in regular *n-i-p* PSCs, enabling state-of-the-art device efficiency.^377,487–489^ However, in its neutral form, Spiro-OMeTAD has intrinsically low conductivity, which limits charge extraction and hinders achieving high device performance.^490–492^ To overcome this limitation, LiTFSI has been extensively employed as a *p*-type dopant to facilitate the formation of Spiro-OMeTAD^+•^ radical cations. Typically, LiTFSI is added to the Spiro-OMeTAD solution, and the resulting perovskite/Spiro-OMeTAD:LiTFSI film is exposed to ambient air for 12–24 h to enable oxygen-assisted oxidation. However, this doping strategy suffers from several drawbacks, including a slow oxidation rate, limited doping efficiency, and the formation of parasitic byproducts, which may negatively impact device performance and stability.^377^ Additionally, Li^+^ from LiTFSI-doped Spiro-OMeTAD readily migrates into the perovskite, degrading performance.^493^ In this sense, alternatives have been developed using Li-free dopants, hosts that immobilize Li^+^, or dopant-free HTLs. For example, MoS_2_-modified Spiro-OMeTAD adsorbs Li^+^ and blocks its migration, improving stability (85% PCE retention vs 30% for the control);^494^ 12-crown-4 complexes Li^+^ via host–guest chemistry, strongly reducing its migration and enabling 95.9% PCE retention over 2400 h;^495^ and ferrocene interlayers bind Li^+^, yielding 70% retention after 1250 h.^496^ Li-free salts such as Zn-TFSI_2_ ^490,497^ or Fe­(F_20_TPP)Cl exhibit higher migration barriers and greater moisture tolerance, thereby avoiding Li-induced instability, while yielding performance comparable to that of Li-based salts.^498^ Ionic liquids, such as BMPy-TFSI, replace both Li-TFSI and tBP, another dopant frequently used to prevent Spiro-OMeTAD aggregation and improve film morphology, while maintaining conductivity and moisture stability.^499,500^ Alternatively, radical-based Spiro-OMeTAD doping eliminates the need for Li-TFSI and enables 25% PCE with excellent durability.^377^

### 5.6 CTL/Electrode Coupling

The interface between the CTL and the electrode governs charge extraction efficiency, series resistance, and long-term device stability. Malfunction at this interface can arise from several coupled effects, including energetic mismatch between the CTL and the electrode, the presence of interfacial defect states, and chemical reactions driven by migrating ionic species from the perovskite absorber. Together, these factors can lead to nonideal electrical contacts and degraded device performance.

#### 5.6.1 Key Properties

From an electronic standpoint, the nature of the CTL/electrode contact is commonly described in terms of ohmic versus Schottky behavior (Figure 6). When the work function of the electrode is lower than or equal to the *EA* of the ETL, Fermi-level equilibration upon contact leads to the electron flow from the *E*_*C*_ to the metal (ohmic carrier extraction). For the hole ohmic contact *WF*_*electrode*_ ≥ *IE*_*HTL*_, upward band bending occurs. In contrast, if the electrode work function is higher than the *EA* of the ETL (for electron contact), Fermi-level alignment results in upward band bending within the ETL and the formation of a depletion region of the thickness *w*, defined by eq 2, giving rise to a Schottky barrier that impedes electron extraction and increases the series resistance. An analogous situation applies to HTLs: efficient hole extraction requires a high-work-function electrode aligned with the *IE* of the HTL, whereas mismatched contacts lead to interfacial barriers and resistive losses. Such Schottky barriers are well-known to reduce carrier injection and extraction efficiency in thin-film photovoltaic devices.

The presence of interfacial defect states further complicates this picture. Structural disorder, chemical imperfections, or damage induced during electrode deposition can introduce localized states within the bandgap of the CTL. These states can pin the Fermi level, modify the effective barrier height, and promote nonradiative recombination. In practice, the Schottky barrier at the CTL/electrode interface is therefore not solely determined by bulk energy levels but is strongly influenced by interface-specific electronic states and their spatial distribution. As a demonstration of optimizing the energy alignment between the ETL and the electrode, Wang et al. introduced a thin In_2_O_3_ film with an *EA* of 4.55 eV to ensure a gradient energy alignment between the ITO and the SnO_2_ (Figure 34).^501^ As a signature result of the improvement band alignment, the *V*_*OC*_ increased by up to 100 meV when both layers were applied, compared to the device with a single SnO_2_ or In_2_O_3_ film.

**Figure 34:**
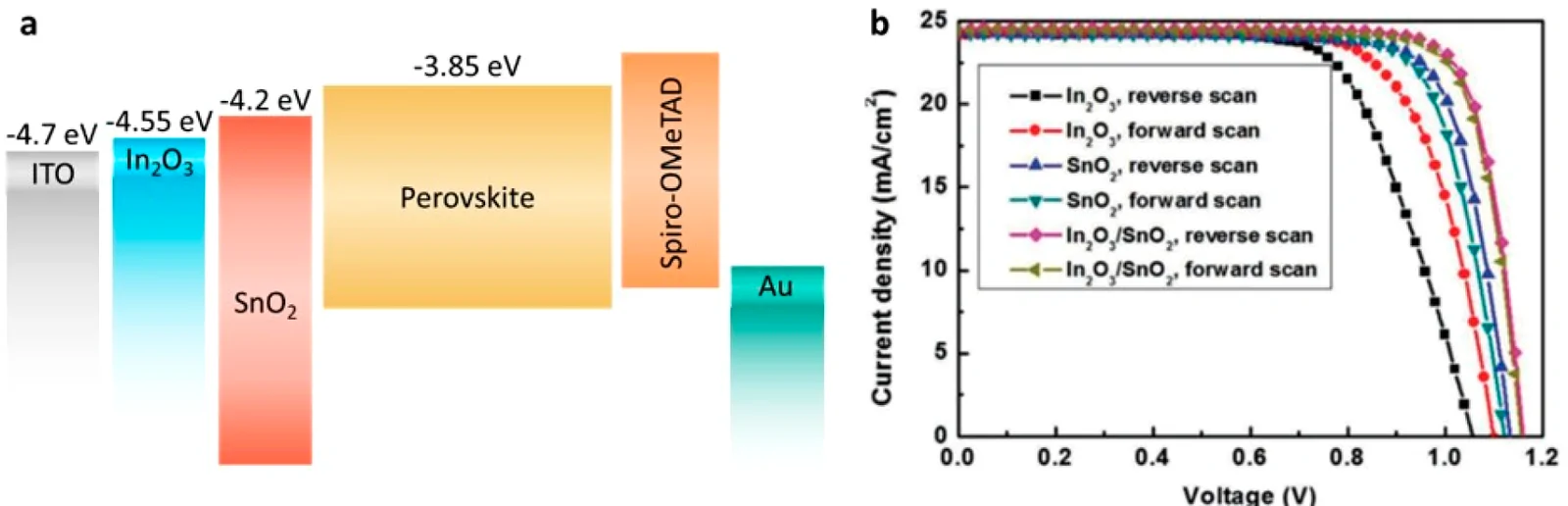
(a) Band diagram of the device with the In_2_O_3_ interlayer. (b) *J*–*V* curves of the device with and without the In_2_O_3_ interlayer. Reproduced with permission from ref 501. Copyright 2020 John Wiley & Sons.

The chemical reactivity at the CTL/electrode interface is an additional and distinctive challenge. Migrating halide ions or metal cations from the perovskite can react with metal electrodes, forming insulating metal halide layers at the interface. These reaction products effectively act as tunneling barriers, impeding charge transfer and substantially increasing the device series resistance. Over time, such processes can also alter the effective work function of the electrode and accelerate device degradation. The matter was discussed in detail in Section 3.1.1 and will also be addressed in Section 5.3.

It is noteworthy that the series resistance arises not only from Schottky barriers and interfacial reaction layers but also from the intrinsic conductivity of the CTL itself. For this reason, transport layers with relatively low carrier mobility or conductivity, such as polymeric hole transport materials, are typically deposited as thin films, often below ∼ 20 nm, to limit resistive losses while maintaining sufficient coverage and uniformity.

Several mitigation strategies have been developed to address these issues. Interfacial dipole engineering is a well-established approach to modulate the effective work function of electrodes, thereby achieving near-ohmic contacts. A rigorous discussion treating the use of dipolar layers was presented in Section 5.1. Introducing ion-blocking layers between the CTL and the electrode can prevent the migration of halides toward the electrode, and of metal ions toward the active layer. In that context, the use of less reactive electrode materials, such as carbon-based contacts or conductive oxides, has emerged as an alternative strategy to improve chemical stability, albeit sometimes at the expense of ideal energy alignment.

#### 5.6.2 Ion Blocking CTL/Electrode Interlayers

The CTL/electrode interface strongly affects PSC stability because ion migration between the perovskite and the metal electrode can lead to the formation of insulating materials that compromise the device performance.^502–505^ Because organic CTLs are typically thin and loosely packed, especially when based or small molecules, they allow ions to leak through nanoscale gaps; thus, strategies to inhibit ion diffusion can rely on the introduction of a thin oxide layer at the CTL/metal interface. For example, an amorphous atomic layer deposited (ALD)-TiO_2_ barrier can be introduced between Spiro-OMeTAD and Au to block Au diffusion toward the perovskite/SnO_2_ interface, thus suppressing ion migration besides improving PCE.^506^ Moreover, amine-mediated TiO_*x*_ (AM-TiO_*x*_) was suggested to prevent Ag corrosion by amines binding to halides, resulting in a stable series resistance for >900 h under N_2_.^507^ Similarly, ALD-AZO or ALD-SnO_2_ layers, introduced between the CTL and the electrode, suppress perovskite decomposition and reduce overall breakdown;^478^ this strategy has proven effective also in large-scale modules.^508^ In the same direction, a PCBM/CeO_*x*_ bilayer ETL provides both efficient electron extraction and chemical shielding, achieving high PCEs and markedly improved stability.^509^ TOF-SIMS depth profiles show that I^–^ migration to the electrode and Ag^+^ back-diffusion into the perovskite are significantly reduced compared to pristine devices. Interestingly, some purely metal interlayers have demonstrated effectiveness in blocking ion migration. For example, Chen et al. used a bismuth layer between the PCBM/BCP and Ag layers.^510^ This layer was effective at protecting the perovskite from moisture and inhibiting iodine-induced Ag corrosion, yielding devices that retain 95–97% of their initial PCE after 500 h at elevated temperatures. This can be explained by the weaker tendency of bismuth to form stable compounds with iodide, compared to Ag.

Other approaches have also been demonstrated using organic materials instead of oxides. For example, benzotriazole (BTA) was introduced between the ETL and the Cu electrode to prevent electrochemical corrosion by forming [BTA–Cu] complexes.^511^ Energy-dispersive X-ray spectroscopy (EDX) and TOF-SIMS show Cu^+^ diffusion is nearly eliminated, resulting in devices retaining 92.8% of initial PCE after 2500 h and 88.6% after 1000 h MPP tracking. Similarly, a TTTS (1,3,5-triazine-2,4,6-trithiol trisodium salt) additive to the bathocuproine (BCP) film prevents Au diffusion by coordinating to the TTTS molecules.^512^ EDX shows strong Au penetration in pristine devices but minimal diffusion in BCP:TTTS devices, giving excellent operational, ambient, and thermal stability. Furthermore, Milić et al.^513^ proposed adding a conductive reduced graphene oxide (rGO) interlayer between the copper thiocyanate (CuSCN) HTL and a gold electrode. The rGO layer serves as both a physical and a chemical barrier, helping maintain the integrity of the device.

#### 5.6.3 Nonmetal Electrodes

Although nonmetal electrodes in perovskite solar cells can sometimes be discussed in terms of energy-level alignment, their primary advantage lies in their relatively chemical inertness toward migrating halide species. In metal-based contacts, ion migration induces interfacial reactions, the formation of metal halide compounds (AgI, for instance), and defect states that dynamically alter the effective work function. These chemically driven processes result in time-dependent and poorly controlled energetic alignment. In contrast, nonmetal electrodes such as carbon, conductive polymers, or transparent conductive oxides could largely suppress interfacial redox chemistry, stabilizing the interfacial electronic structure. As a consequence, the energy-level alignment in these devices should not be impacted by the metal-halide reaction and the possible metal ion migration, allowing to remove one degree of freedom in the interpretation of the measured characteristics.

Alternative electrode strategies have been explored that move beyond conventional metals (e.g., Ag, Au, Cu). Transparent conductive oxides,^514–516^ low-reactivity metal alloys, or metal bilayers based on less reactive metals such as Bi or Cr have been introduced to mitigate halide-induced corrosion while maintaining acceptable contact energetics.^505,517,518^ Although such approaches may compromise absolute conductivity or optical reflectivity relative to Au or Ag, they offer a pragmatic balance between energetic compatibility and chemical robustness. In this context, carbon-based electrodes represent a particularly compelling solution: their intrinsic chemical inertness toward migrating halides, combined with hydrophobicity and sufficient conductivity, enables stable charge extraction without relying on reactive metal contacts. As a result, carbon electrodes decouple interfacial energetics from corrosion-driven degradation, making them especially attractive for CTL-free and long-term stable perovskite solar cells.^505,519^ This stabilization of interfacial energetics is particularly relevant for CTL-free architectures, where charge selectivity must emerge from interfacial band bending and ion-mediated electrostatics rather than from dedicated transport layers.

Overall, while the CTL/electrode interface is often considered a secondary factor compared to the perovskite/CTL junction, it still plays a significant role in determining contact resistance, charge extraction efficiency, and operational stability. A rigorous understanding of Schottky versus ohmic behavior, coupled with careful control of interfacial chemistry, is therefore essential for the rational design of high-performance and durable perovskite solar cells.

## 6 Alternative Concepts for Controlling Device Energetics

Beyond the strategies for modifying interfaces discussed in Section 5, alternative concepts for energetic control of perovskite solar cells include fabricating charge-transport layer-free devices, using nonmetallic electrodes, and forming an energetic gradient within the perovskite active layer. These approaches, while less common and investigated, offer potential advantages for the commercialization of perovskite solar cells, as they can minimize the number of processing steps required for device fabrication. In the following, we outline the state of progress in the development of these approaches and discuss the challenges associated with their realization.

### 6.1 CTL-Free PSCs

The removal of CTLs is motivated by the industry’s need for simpler device fabrication. The inclusion of CTLs adds additional steps to the manufacturing process and often requires high-temperature annealing, a step commonly used to prepare metal oxide-based ETLs in *n-i-p* PSC architecture, thereby increasing production costs. As a result, significant research efforts are focused on developing ETL-free device architectures. Interestingly, the remarkable hole mobility of perovskite materials, along with their long carrier diffusion lengths, ambipolar charge-transport capabilities, and low exciton binding energies, also presents an opportunity to eliminate HTLs. In this sense, to simplify device architecture and reduce fabrication costs, various strategies have been employed to design PSCs without ETLs or HTLs, collectively referred to as ETL-free, HTL-free, or, more broadly, CTL-free PSCs. Given the breadth of reported CTL-free device configurations, a consolidated comparison of structures and efficiencies is presented in a dedicated review article.^520^

The operation of CTL-free devices relies on favorable interfacial energetics and built-in electric fields within the perovskite absorber, in contrast to conventional structures where engineered transport layers govern selectivity and extraction. In ETL-free *n-i-p* devices and HTL-free *n-i-p* devices, efficient carrier extraction requires appropriate work-function alignment between the TCO and the relevant perovskite band edge (*E*_*C*_ for ETL-free, *E*_*V*_ for HTL-free), enabling selective extraction while blocking the opposite carrier type. In many cases, a metal–semiconductor contact forms at the TCO/perovskite interface, driving charge transfer through interfacial band bending.

In HTL-free *n-i-p* cells, a perovskite/ETL *n-p* heterojunction creates a depletion region that establishes a built-in potential, assisting charge separation and suppressing interfacial recombination. The extent of this depletion region, and thus charge-collection efficiency, depends strongly on ETL thickness; for example, a depletion width of ∼ 395 nm in a ∼ 620 nm TiO_2_ layer enabled a PCE of 7.2% in an HTL-free FTO/*c*-TiO_2_/*m*-TiO_2_/MAPbI_3_/Au device.^521^ A milestone in HTL-free device development was reported by Etgar et al. in 2012, proposing a simple device architecture ITO/m-TiO_2_/MAPbI_3_/Au, achieving a PCE of approximately 5.5%^522^ and by Hu et al., who later introduced an HTL-free inverted PSC with a structure of ITO/MAPbI_3_/C_60_/Ag, achieving a PCE of 5.4%.^523^ Since then, notable progress in this approach has been observed, driven by increased research interest. Improvements in film morphology, reduction in defect densities, and optimized interfacial engineering have collectively enhanced device performance. To date, CTL-free PSCs have surpassed PCEs of 20%,^524,150^ demonstrating remarkable potential for scalable, cost-effective photovoltaic technologies.

#### 6.1.1 Carbon-Based Electrodes for PSCs

An alternative to metal electrodes is carbon-based materials, which have a high work function and are good hole collectors. Additionally, carbon materials offer several advantages. For example, they are cost-effective, chemically inert with MHPs, and can be processed at low temperatures. Additionally, carbon electrodes eliminate the need for expensive vacuum-assisted thermal evaporation methods generally used for metals. Furthermore, their hydrophobic nature also helps to protect the perovskite from moisture, thereby improving device longevity.

Li et al. demonstrated a device comprising a mesoscopic TiO_2_/Al_2_O_3_ framework, a MAPbI_3_ absorber, and a carbon electrode, achieving a significantly improved PCE of 14.7%.^525^ Han’s group demonstrated mesoporous carbon-based PSCs with a verified PCE of 12.8%, and >1000 h stability under illumination.^526^ They later developed printed HTL-free mesoscopic PSCs using (5-AVA)_*x*_MA_1–*x*_PbI_3_ perovskites (5-AVA: 5-ammonium valeric acid).^527^ Under light, heat, and bias, MAPbI_3_ undergoes MAI loss and ion migration, whereas 5-AVAI strengthens grain boundaries and interfaces via hydrogen bonding, thereby suppressing MA^+^/I^–^ migration. These devices passed tests with >9000 h of operation. Kobayashi et al. attributed the suppression of ion migration to a quasi-2D perovskite/oxide interface formed by 5-AVAI and MA^+^, which enhances charge transport and stabilizes ion movement.^528^ Ion migration is light-activated, leading to interfacial charge buildup, performance enhancement, and reversible relaxation during storage. Ku et al.^529^ demonstrated the use of a carbon black/spherical graphite composite as a rear electrode, achieving a PCE of 6.64%. More recently, PSCs with carbon electrodes reached a certified PCE of 21.9%,^530^ which was later improved to 22.2% in 2024, accounting for a passivation defect observed in the perovskite and electron transport layer.^531^

#### 6.1.2 Ionic Double Layers

Because mobile ions in metal halide perovskites can accumulate at contacts under illumination or bias, ionic double layers (and interfacial dipoles) may spontaneously form at the perovskite/electrode interfaces and give rise to local electrostatic fields that alter band alignment and carrier extraction. These self-assembled fields can, in principle, contribute to charge separation in CTL-free architectures by inducing interfacial band bending that favors selective extraction of one carrier type, rather than relying solely on fixed semiconductor junction properties. Indeed, several studies have shown that ion accumulation at selective contacts or at perovskite interfaces correlates with changes in open-circuit voltage, *J–V* hysteresis behavior, and surface potential profiles, indicating that ionic redistribution strongly influences the internal field landscape.^134,265^ However, direct experimental evidence specifically demonstrating that stable ionic double layers alone are responsible for efficient CTL-free device operation is limited, in part because the interfaces in working devices are dynamic, highly defect-rich, and subject to simultaneous electronic and ionic screening. While the concept of ion-mediated interfacial energetics provides a valuable framework for interpreting certain charge separation phenomena in CTL-free PSCs, more targeted *in-operando* measurements and validated modeling are required to confirm whether ionic double layers can independently sustain selective carrier extraction in the absence of conventional transport layers.

#### 6.1.3 CTL-Free but Not Interlayer-Free

With that in mind, it is worth noting that CTL-free devices do not always have to be interlayer-free. Dedicated passivation layers are still necessary to prevent direct contact of the migrating halide (or halide vacancy) with the reactive metal electrode and to adjust the *WF* of the adjacent electrode. Concerning the latter, a common remedy is to introduce a dipolar layer at the TCO, which typically has a low *WF*. For example, poly­(ethylene oxide) (PEO) at the perovskite/carbon electrode interface improved charge transfer by adjusting the energy levels between the two materials.^532^ On TCOs, self-assembled polar molecules can induce dipoles and reduce the work function: a modification with dipolar molecules decreased the work function of ITO from 4.43 to 5.06 eV, enabling a more favorable ITO/perovskite contact.^275^ Polymeric layers such as polyethylenimine ethoxylated (PEIE) at the FTO/perovskite junction have also been shown to lower the TCO work function and facilitate electron extraction.^533^ Ionic liquids with hydroxyl groups, such as 1-([1,1’-biphenyl]-4-yl)-3-(2-hydroxyethyl)-1H-imidazol-3-ium iodide (BIPH-II), chemisorb onto TCO surfaces, introducing dipoles that shift the work function and improve electron extraction.^533^ Inorganic acids can serve a similar role by tuning the ITO work function, thereby improving energy alignment.^533^ Finally, organic monolayers forming covalent bonds with ITO, such as N–Sn linkages, provide another route to control the electrode work function and reduce the interfacial barrier.^533^

An interesting example is CeO_*x*_, which is sometimes used as a bilayer with TiO_2_ or with SnO_2_.^534,535^ Even though CeO_*x*_ is likely to facilitate electron transport due to its favorable CBM, the layer is usually too thin to enable bulk charge transport. Therefore, the interlayer of CeO_*x*_ is instead discussed in terms of defect passivation and recombination control.

Collectively, these examples illustrate a continuum of device architectures, spanning from conventional semiconductors with bulk electron or hole transport to architectures that dispense with charge-transport layers altogether by relying on favorable electrode work-function alignment. Between these extremes is a wide range of surface treatments and functional interlayers, such as defect passivators, dipolar layers, and ion-blocking layers, that do not support measurable carrier transport within their bulk. Despite this, these layers play a crucial role in shaping the device’s energetic landscape.

#### 6.1.4 Toward CTL-Free Devices: Energy Gradient

##### 6.1.4.1 Phase Heterojunction

The concept of phase-heterojunctions in all-inorganic perovskites has recently been demonstrated using polymorphic CsPbI_3_, where the β- and γ-phases are intentionally combined within a single device.^263,536^ In γ-CsPbI_3_/β-CsPbI_3_ architectures, the favorable energetic offset between the two phases increases the built-in potential, improving band alignment for charge separation, while the wider-bandgap γ-phase also contributes to surface passivation of the β-phase. Phase-heterojunction devices exhibit simultaneous enhancements in *V*_*OC*_ and fill factor compared with single-phase counterparts, leading to power conversion efficiencies exceeding 20%. There is, however, an ongoing debate over the direction of the funneling effect, with two studies proposing substantially different band diagrams and consequently demonstrating both *n-i-p* and *p-i-n* architectures as optimal. Given the lack of IPES or operando spectroscopy, the improved photovoltaic response may also arise from contributions from other effects, such as reduced interfacial recombination, phase-dependent self-doping, and electrostatic effects, rather than a uniquely defined band-edge alignment.

##### 6.1.4.2 Energy Gradient in Mixed Halide Perovskites

Mixed-halide metal halide perovskites exhibit an intrinsic tendency toward halide redistribution under illumination and/or strain.^537^ The effect was mainly observed for Br/I formulations, in which the system tends to form I-rich regions with low bandgap, and Br-rich domains with wide bandgap.^538^ This phenomenon has been consistently observed across a wide range of material systems, device architectures, and length scales, including both polycrystalline thin films and single crystals.^539–541^ The process is generally attributed to the relatively low migration barriers of halide vacancies and to the coupling between ionic motion and photogenerated carriers,^542,543^ and is consistent with the finding that the addition of Cs^+^, known to limit ion migration, decreases the halide segregation effect.^544^ It further leads to the formation of an internal energy landscape characterized by the gradient of *IE* across the film (Figure 35a).^545^ This effect was demonstrated by the evolution of the photoluminescence (PL) peaks, characteristic of single-halide formulations, as a function of illumination time (Figure 35b) or by differences in carrier lifetime measured on different sides of the crystal (Figure 35c).^545^

**Figure 35:**
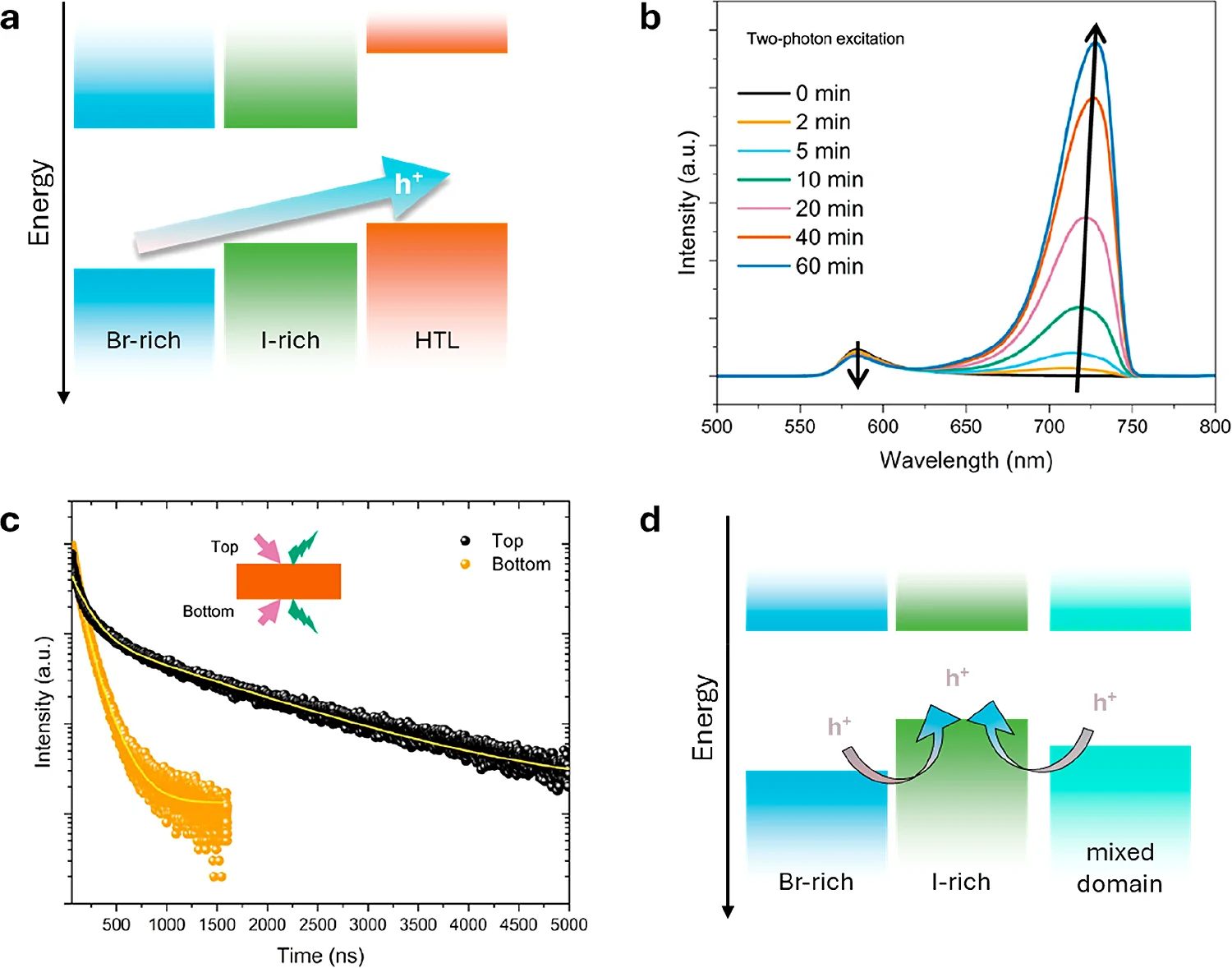
(a) Carrier funneling from a wider bandgap of the Br-rich domain to a narrower bandgap of the I-rich domain in a mixed-halide PSC. (b) PL intensity of a mixed Br/I-perovskite single crystal as a function of the time under illumination, triggering the formation of I-rich domains (c) Time resolved (TR) PL of the same single crystal after the light exposure, measured on both sides, evidencing the halide redistribution. (d) Schematic diagram of carrier trapping at the I-rich domain. Panels b and c were reproduced with permission from reference^545^ under the Creative Commons CC-BY-NC-ND license. Nature Springer.

Interestingly, time-resolved (TR) PL measurements indicate that the charge-carrier lifetime in the mixed-halide systems is shortened by charge trapping in the I-rich domains of a smaller bandgap, a phenomenon that is fully reversed in the dark (Figure 35c,d).^546^ Surprisingly, halide segregation does not substantially modify the intrinsic carrier mobility within the perovskite lattice. Charge transport parameters, such as mobility, suggest that segregation primarily alters the *energetic* rather than the *kinetic* aspects of carrier dynamics.^547^ As a result, the dominant electronic consequence of segregation is the redistribution of carriers in real space rather than enhanced transport through improved mobility.

It is worth noting that the impact of halide segregation on device efficiency remains ambiguous. On the one hand, the formation of I-rich regions near charge-selective interfaces can locally align band edges and, in certain device configurations, facilitate hole extraction. On the other hand, the same segregation process can introduce additional recombination pathways at compositional interfaces or induce temporal instability in the device response. Furthermore, the partial recovery of halide segregation upon returning the film to dark conditions after periods of illumination could, in principle, lead to a recovery of device efficiency during repeated day–night cycling (when considering real outdoor applications). However, the resulting halide distribution is unlikely to match the initial, as-prepared state. Such irreversible or metastable rearrangements may, for example, lead to localized increases in defect concentration or to nonoptimal band bending at grain boundaries and intragrain interfaces. Consequently, these effects raise critical questions regarding the long-term compositional reliability and operational stability of multihalide metal halide perovskites.

To demonstrate the complexity of halide segregation, Datta et al. prepared FA_0.66_MA_0.54_Pb­(I_0.67_Br_0.33_)_3_ films and demonstrated that they exhibit a pronounced red shift and enhancement of the photoluminescence signal under prolonged illumination, consistent with the emergence of iodide-rich, lower-bandgap domains that act as sinks for photogenerated electrons and holes and promote radiative recombination. While such photoinduced segregation can make efficient charge extraction difficult and contribute to *J*_*SC*_ losses, its impact on the open-circuit voltage appears considerably weaker, with *V*_*OC*_ remaining relatively stable despite the presence of energetically narrower domains. This apparent decoupling highlights the unresolved and potentially competing roles of halide segregation in determining device efficiency and operational stability.^548^ Importantly, because halide redistribution occurs spontaneously under typical operating conditions, it is difficult to establish an actual “segregation-free” control device, limiting the ability to unambiguously assess whether the effect is beneficial or detrimental to performance.

From a stability perspective, halide segregation is generally reversible upon removal of illumination or bias, indicating that it does not necessarily correspond to permanent chemical degradation. However, as previously highlighted, repeated segregation–recovery cycles may contribute to long-term operational instability through indirect mechanisms, such as enhanced local strain, defect accumulation, or interfacial reactivity, although these links remain incompletely resolved.

Taken together, these observations suggest that halide segregation should be viewed not as an intentional optimization strategy, but as an emergent, and largely unavoidable property of mixed-halide perovskites. Its primary consequence is the dynamic reshaping of the internal energy landscape under operation, rather than a fundamental modification of charge transport. Recognizing and accommodating this behavior, rather than attempting to eliminate it, may therefore be a more realistic approach when designing stable and efficient mixed-halide perovskite devices.

##### 6.1.4.3 Dimensionality

Mixed-dimensional (2D-3D, 1D-3D, or 0D-3D) perovskite heterostructures are now widely recognized as an effective defect-management strategy, particularly at surfaces and grain boundaries, where low-dimensional phases selectively form and passivate electronically active defects.^477,549–551^ It is also widely recognized that the addition of low-dimensional (LD) phases improves stability, especially against moisture-assisted degradation.^552^ In parallel, band-alignment effects between LD and 3D perovskite phases are frequently invoked to rationalize enhanced device performance. While it is evident that LD phases have generally higher band gaps than their 3D counterparts, it is unclear whether the 3D/2D band alignment is hole- or electron-selective, with the literature offering examples supporting both sides of the debate. For instance, Hui et al. outlined a method to create stable ETL-free FAPbI_3_ PSCs by forming a low-dimensional perovskite layer directly between the FTO and the perovskite.^524^ The authors propose that this interlayer induces energy band bending, reduces defect density at this interface, and establishes indirect contact, thereby enhancing energy level alignment between the electron-collecting electrode and the perovskite. These changes promote charge carrier transport and collection while minimizing recombination. In another example, different spacer cations, namely BA^+^, PEA^+^, and EA^+^, were used as a 2D capping layer for a triple cation perovskite.^553^ The band-edge positions of the 3D/2D interfaces, extracted by combining scanning tunneling spectroscopy and KPFM, showed that the EA^+^-based 2D perovskite establishes the most favorable type-II alignment for the electron extraction (Figure 36a–c). On the other hand, an HTL-free device with a carbon top electrode and a 3D/2D heterojunction is also demonstrated, in which the 2D capping layer improves the hydrophobicity of the film and passivates surface defects.^554^ The authors also suggest an improved band alignment that facilitates the hole transport toward the carbon electrode (Figure 36d–f). An insight into how the interfacial energetics of 2D/3D perovskite heterostructures vary in the dark and under illumination was obtained by combining cross-sectional STM/dI/dV spectroscopy.^555^ Spatially resolved dI/dV spectra recorded in the dark revealed a gradual shift of both conduction and valence band edges across the 2D overlayer, resulting in a progressively widened bandgap and a *p*-type character toward the surface, consistent with enhanced hole extraction at the 2D/HTL interface (Figure 36g,i). Under illumination, light-modulated measurements showed pronounced photoinduced band bending, with opposite band shifts in the 3D and outer 2D regions, leading to a strengthened internal electric field within the 2D layer (Figure 36h,j). By combining spatially resolved spectroscopy with controlled illumination, this work accesses *quasi*-*in-operando* interfacial energetics, capturing dynamic band bending at 3D/2D heterojunctions and narrowing the gap between static *ex-situ* measurements and real operating conditions.

**Figure 36:**
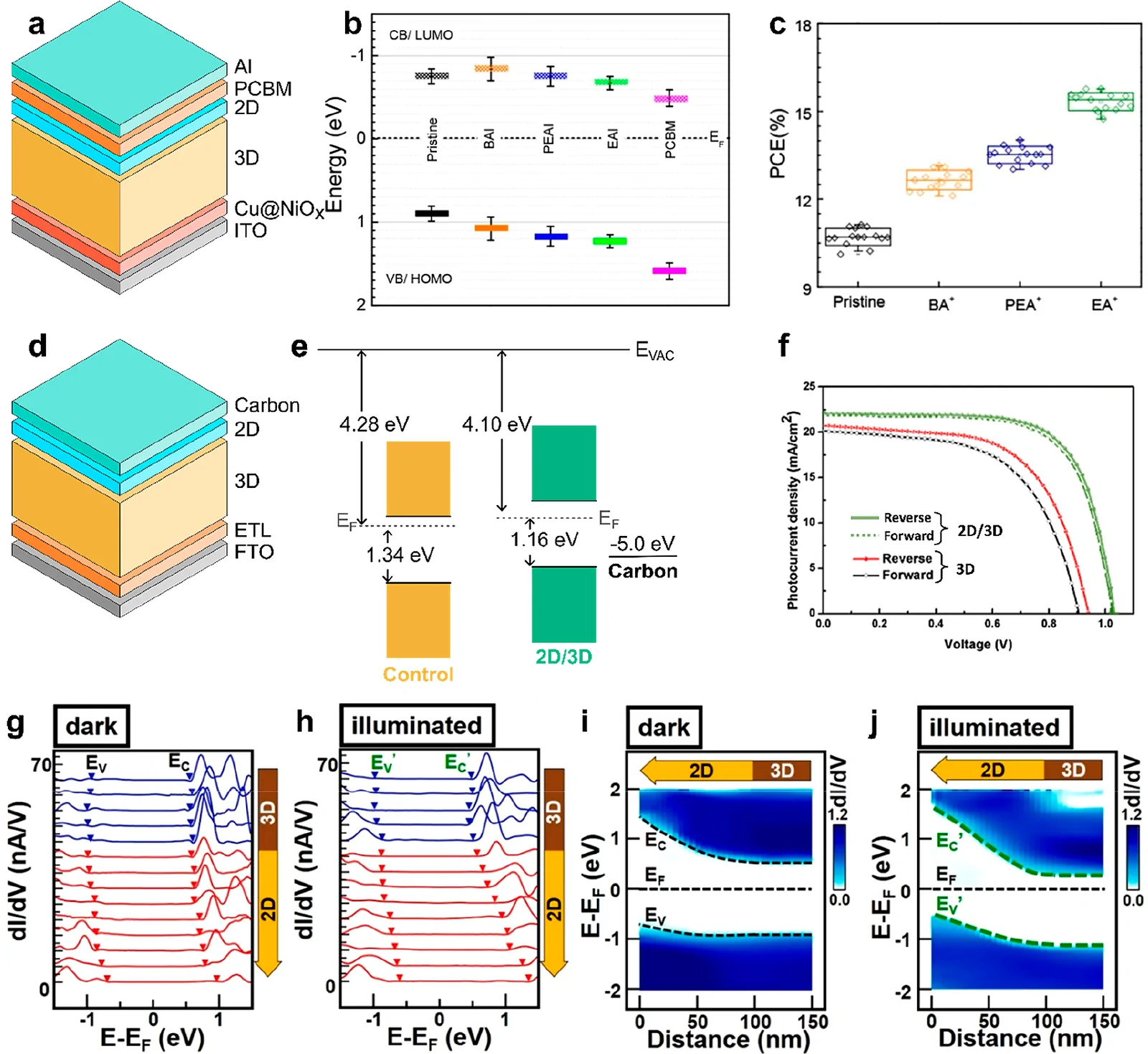
(a) Solar cell architecture with the electron-selective 2D capping layer from reference^553^ (b) *E_C_* and *E_V_* of the 3D perovskite and the 2D capping layers made with different organic cations. (c) PCE of the solar cells with the 2D capping layers made with different organic cations; (d) solar cell architecture of the device with the hole selective 2D capping layer from^556^ (e) band diagram of the 3D and 2D perovskite, together with the *WF* of the carbon electrode; (f) IV characteristics of the solar cell without and with the 2D capping layer; (g) electronic dI/dV curves of the 2D/3D interface in the dark and (h) under illumination (515 nm); (i) Band alignment of the 2D/3D perovskite interface mapped in the dark and (j) under illumination (515 nm), extracted from the positions of the *E_C_* and *E_V_* levels in g and h, respectively, indicated by the triangular marks i. (a–c) Reproduced with permission from ref 553. (d–f) Reproduced with permission from ref 556. (g–j) Reproduced with permission from ref 557 under Creative Commons CC-BY license.

In many reports, proposed band diagrams are commonly inferred from optical bandgap measurements and surface-sensitive UPS, rather than from direct determinations of conduction-band offsets or buried interfacial energetics. Consequently, claims of improved energetic alignment in mixed-dimensional heterojunctions should be interpreted with caution. The carrier selectivity of such heterojunctions is highly sensitive to the specific material formulation and interfacial chemistry, as even minor compositional variations can induce subtle shifts in band-edge energies, often on the order of only a few hundred millielectronvolts. First-principles calculations further suggest that the resulting band alignment may strongly depend on the surface termination of the 3D perovskite phase, which is difficult to control and experimentally verify.^558^ Moreover, the actual energetic landscape under device operating conditions, including dynamic band bending and ion-mediated interfacial effects, remains largely unresolved. Consequently, while favorable energetic offsets may exist in specific mixed-dimensional architectures, their contribution to device performance is likely secondary to defect passivation and recombination suppression in most practical devices.

Similar to light-induced halide redistribution, mixed-dimensional heterojunctions are a case in which spatially varying composition modifies local energetics and recombination pathways. In both cases, performance improvements are experimentally evident, while the microscopic energetic landscape remains dynamic and difficult to define uniquely. Despite extensive device-level evidence, direct experimental determination of band offsets at mixed-dimensional perovskite interfaces remains limited. Advanced approaches such as IPES, hard X-ray photoelectron spectroscopy, or operando spectroscopic techniques will be essential to unambiguously resolve the energetic landscape of these heterojunctions.

In summary, mixed-dimensional perovskite heterojunctions have emerged as an effective strategy for defect passivation and stability enhancement in perovskite solar cells, particularly by selectively forming low-dimensional phases at surfaces and grain boundaries. These phases efficiently suppress nonradiative recombination and ion migration, thereby prolonging charge-carrier lifetimes and improving operational stability. Although favorable band alignment between LD and 3D perovskites is frequently proposed to explain performance gains, direct experimental verification of conduction- and valence-band offsets at these buried interfaces remains scarce. In practice, interfacial electrostatic effects, dipole formation, and defect passivation are likely to play a dominant role in governing device behavior, with energetic alignment playing a secondary, architecture-dependent role.

##### 6.1.4.4 Defect Control

Metal halide perovskites are known to be susceptible to the intrinsic defect doping (see discussion in Section 2.1). For example, halide vacancies are known to carry a negative charge and impart mild *n*-type doping, while *V*_*Pb*_ can lead to *p*-type doping.^63,118,559^ Specifically, adjusting the PbI_2_/MAI ratio in MAPbI_3_ modulates the Fermi level and conductivity type.^63,250,559–563^ Cui et al. fabricated the *n-p* homojunction by varying the precursor ratios and combining spin coating, thermal evaporation, and dip coating; they were able to obtain *p*- and *n*-type MAPbI_3_ (Figure 37a).^560^ The built-in junction enhanced charge separation and extraction (Figure 37b). Using the same idea, one can conceptualize a device structure based solely on perovskites, i.e., CTL-free. For example, using the regular *n-i-p* PSC architecture, it should be possible to create a high-bandgap bottom *n*-type MHP layer by introducing donor point defects to the perovskites, followed by an intrinsic MHP layer, and a top layer of a *p*-type perovskite. This type of heterostructure, although theoretically feasible, may suffer from significant limitations. A notable phenomenon is halide diffusion, which can alter the composition of the heterostructure over time, compromising its integrity and, consequently, the device’s performance. However, it is worth mentioning that the doping effect is often limited by self-compensation mechanisms and the relatively low formation energy of counteracting defects. Therefore, the ability of intrinsic defects to effectively dope a material is governed by a balance among formation energy, charge-neutrality constraints, and defect–defect interactions, and is highly material- and condition-dependent.

**Figure 37:**
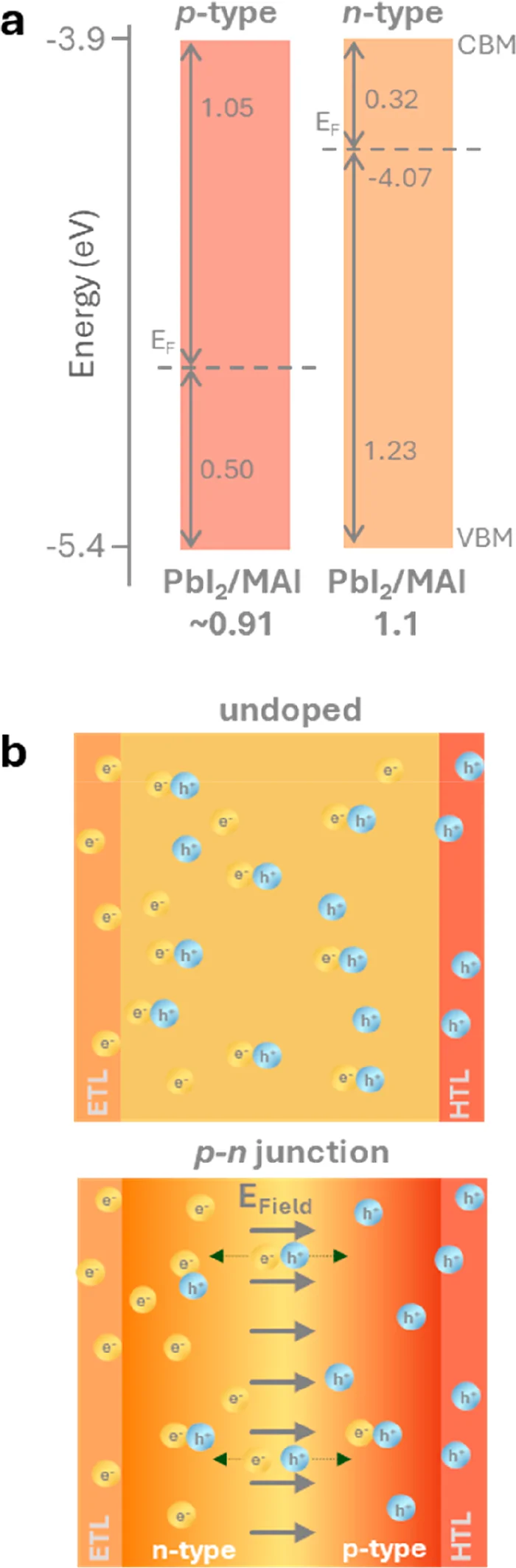
(a) Energy levels of the *p*-type and *n*-type perovskites and the (b) corresponding schematics showing the carrier generation and transport within the perovskite layer in a PSC employing the *p-n* junction. The electric field *E_Field_* drives the separation of electrons and holes toward the charge-selective contacts, as indicated by the dotted green arrows. The yellow and blue circles represent the electrons and the holes, respectively. Adapted with permission from ref 560. Copyright 2019 Springer Nature.

In parallel, several studies support that stoichiometry management not only tunes the doping type but also improves bulk transport properties. Hu and co-workers showed that introducing a slight PbI_2_ excess extends carrier lifetimes in MAPbI_3_-based ETL-free devices, which is consistent with reducing detrimental acceptor-type defects and enhancing the effectiveness of internal fields for charge extraction.^564^ Their composition-dependent analysis confirmed that mixed-cation/mixed-halide perovskites (FA_0.83_Cs_0.17_Pb­(I_0.6_Br_0.4_)_3_) exhibit substantially longer lifetimes than MAPbI_3_, correlating with improved efficiencies in ETL-free configurations. The authors also attributed this behavior to the formation of a more conductive *n*-type perovskite and relocation of the diode junction toward the perovskite/HTL contact when excess PbI_2_ acts as an *n*-dopant. Similarly, excess MAI has been shown to increase hole density and shift the junction to the ETL/perovskite interface, enabling HTL-free device operation.^565^ Together with the observation of higher carrier lifetimes under the appropriate defect doping regime, these studies reinforce that controlled defect-driven doping, achieved by simple stoichiometry tuning, offers a practical pathway for forming efficient internal p–n junctions in CTL-free PSCs while maintaining strong charge-collection fields.

##### 6.1.4.5 Doping

Intentional extrinsic doping in MHPs is analogous to established strategies in conventional semiconductors. represents a promising route to tune band alignment in PSCs, potentially eliminating the need for CTLs and simplifying device architecture. Either *p*-type or *n*-type perovskite doping can be pursued, enabling HTL-free and ETL-free configurations, respectively (Figure 38). For instance, *p*-type behavior in MAPbI_3_ has been achieved through molecular charge-transfer doping using F4TCNQ, where the LUMO level of the dopant is energetically close to the VBM of the perovskite, thereby facilitating the hole transfer from the dopant to the perovskite.^150^ Conversely, *n*-type doping has been demonstrated by incorporating Sm^2+^ ions into MAPbI_3_, where the in-lattice oxidation process yields free electrons as a beneficial byproduct.^40,566^ In both cases, the simplified device stacks exhibited reduced series resistance, highlighting the functional advantage of successful doping.

**Figure 38:**
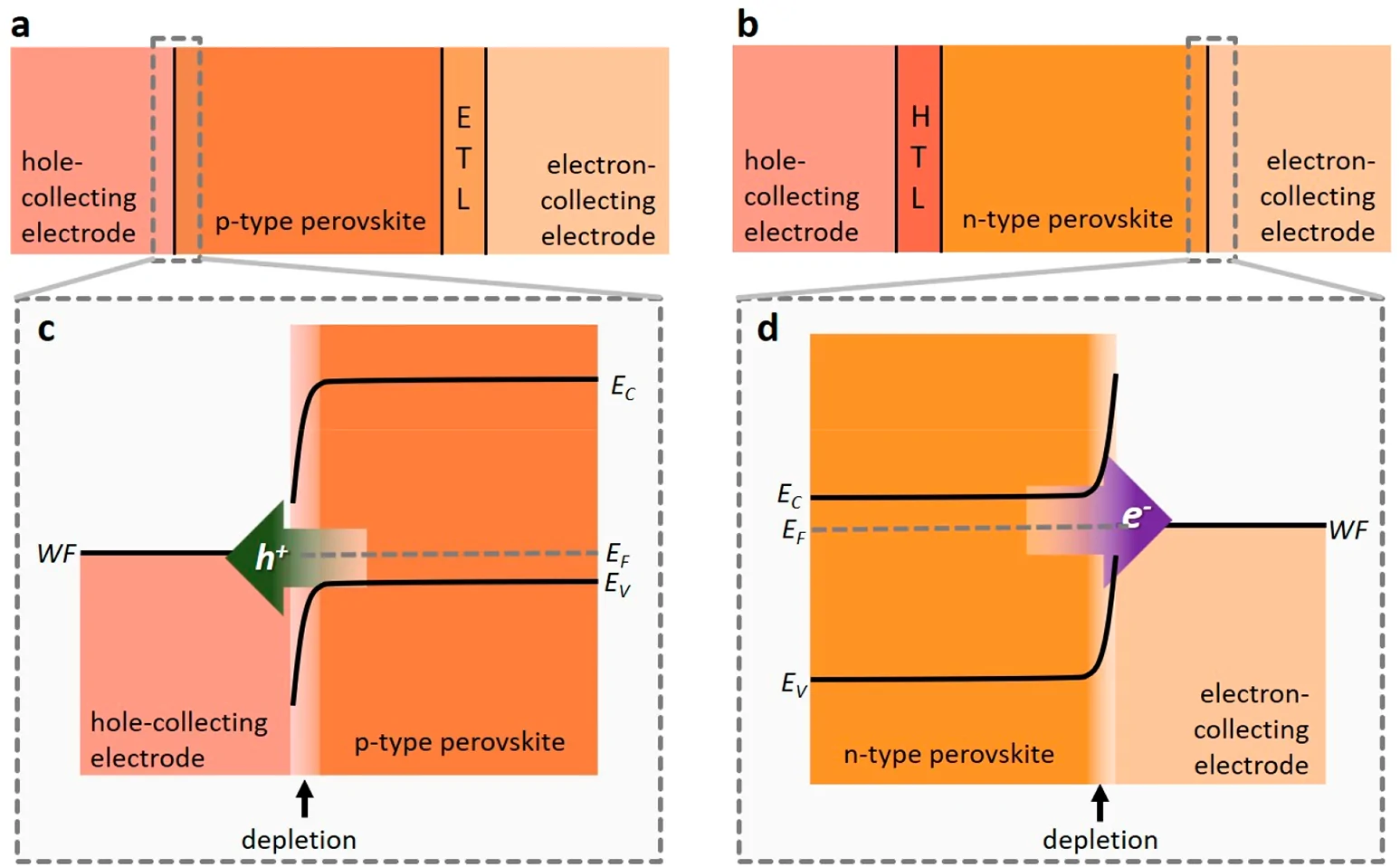
Device architecture using (a) *p*-type and (b) *n*-type perovskite. (c) and (d) are the corresponding energy diagrams representing the band bending at the semiconductor-metal junction for the *p*- and *n*-type perovskite, respectively. Tunneling through the depletion layer is possible if it is sufficiently thin.

Importantly, the role of doping in perovskite photovoltaics differs from the previously discussed interfacial alignment of the VBM or CBM of the semiconductor with metallic contacts. Rather than modifying the interfacial conduction or valence band edges, doping shifts the Fermi level of the perovskite itself. Upon contact with a metal, Fermi-level equilibration induces band bending near the interface, forming a depletion region of width *w*, depending inversely on the concentration of electrically active dopants *N*_*d*_ (eq 2). At sufficiently high *N*_*D*_, *w* becomes small enough to allow carrier tunneling through the barrier, enabling the charge extraction without a dedicated CTL (schematic cases for *p*- and *n*-type shown in Figure 38).

However, the effectiveness of doping remains strongly dependent on the dopant incorporation mechanism and the stability of the resulting electronic states. The highly ionic bonding nature of halide perovskites increases the likelihood of compensating defect formation, particularly in heterovalent substitution, which can counteract the intended doping effect and suppress free-carrier introduction. Thus, while extrinsic doping is an appealing strategy for tailoring perovskite electronic properties, its realization requires careful management of defect chemistry and dopant energetics. More broadly, perovskite doping represents a powerful and versatile strategy for interfacial and bulk energy-level engineering. However, its mechanisms, limits, and device implications are sufficiently rich to warrant dedicated treatment and are therefore beyond the scope of this article; a focused discussion will be presented in a subsequent contribution.

## 7 Conclusions

Despite significant advancements in understanding and controlling energetic alignment in MHP-based optoelectronic devices over the past two decades, the field is still developing, and many aspects remain to be understood. The literature presents a myriad of conflicting reports, ranging from the ionization energies of different MHPs to the selection criteria for charge-extraction materials. Such disagreements likely result from the metastability of the perovskite interfaces, which can be triggered by environmental factors such as humidity, oxygen, temperature, and light, as well as by operating conditions like bias, electric fields, and ion migration.

In contrast to organic photovoltaics, where band alignment is a primary factor governing device performance, perovskite solar cells exhibit a more complex behavior in which initial energy level alignment does not necessarily dictate operational efficiency. This deviation arises from the intrinsic properties of perovskites, including their high carrier density, mobile ions, and soft lattice, all of which enable dynamic redistribution of charges and internal fields. As a result, the energy landscape at interfaces can evolve under working conditions, where the illumination and external bias are present. Conventional UPS, XPS, and IPES provide only a static picture under high vacuum, which misses key degradation pathways. This highlights the importance of in-operando characterization techniques for capturing the dynamic nature of energy level alignment in PSCs. A systematic adoption of such approaches will be crucial for disentangling intrinsic instabilities from extrinsic, processing-related effects. This, in turn, opens the possibility of designing more stable interfaces by chemically manipulating defect centers while creating a favorable, long-lasting band alignment. At the same time, it is important to recognize that even such measurements can perturb the system, for instance, through the illumination used during probing, further emphasizing the need for careful interpretation of experimental results.

The long-term stability of modifications introduced into the perovskite, whether at the interfaces or within the bulk, is yet another critical issue. For instance, small molecules used for defect passivation or for inducing interfacial dipoles can migrate over time and permeate into the perovskite layer, thereby altering their passivation efficacy or the magnitude and orientation of the dipole. Additionally, low-dimensional/3D perovskite architectures may experience dynamic halide and cation exchange across the interface, leading to compositional changes on both sides of the heterojunction. Even extrinsic species that appear chemically benign can participate in undesired reactions over extended operation times, ultimately affecting phase purity, interfacial energetics, and overall device lifetime.

The control of interfacial energetics involves careful selection of charge-selective materials and defect passivation methods, as well as a deep understanding of how these layers behave under operating conditions. Consequently, the emergence of CTL-free device concepts, which challenge the traditional paradigm of optimizing every interface, is an interesting new direction. CTL-free devices are particularly attractive to industry because they simplify fabrication, lower material costs, and reduce the number of potentially incompatible heterojunctions, all of which can accelerate the large-scale commercialization of perovskite technologies. In principle, CTL-free devices can rely on selective contacts, but it is challenging to identify two conductive materials with sufficiently distinct work functions that also remain compatible with MHPs. As an alternative, controlled doping within the perovskite or at the interfaces with the conductive materials can be employed to form internal homojunctions and provide carrier selectivity. While still in their infancy, CTL-free perovskite solar cells highlight that minimizing interfaces may, in some cases, be as effective as engineering them, an idea that could reshape how energetic alignment is approached in future device architectures.

Complementing experimental advances, numerical simulations are becoming indispensable for disentangling the interplay between electronic structure, ionic motion, and interfacial phenomena in perovskites. Atomistic and continuum-scale models can reveal how local dipoles, defect distributions, or strain fields influence band bending and charge selectivity, while device-level simulations help connect these microscopic effects to measurable performance metrics. Crucially, simulations can probe regimes difficult to access experimentally, such as long-term ion migration and transient operating conditions, offering predictive insights into stability and guiding the rational design of interface modifications.

Together, these directions highlight that the future of interfacial energetics research will depend not only on refining our physical understanding but also on equipping the field with dynamic characterization tools and data-driven methodologies capable of addressing the intrinsic instability of perovskites.

## Abbreviations

[EMIM]­PF_6_: 1-Ethyl-3-methylimidazolium hexafluorophosphate
AFM: atomic force microscopy
APT: atom probe tomography
APTES: 3-amino-propyl triethoxysilane
ARUPS: Angle-Resolved Ultraviolet Photoemission Spectroscopy
BMIMPF_6_: 1-Butyl-3-methylimidazolium hexafluorophosphate
BV: benzyl viologen
CB: conduction band
CBM: conduction band minimum
CFSYS: Constant final state yield spectroscopy
CPD: contact potential difference
CPE: conjugated photoelectrolytes
CRPH: carrier-resolved photo-Hall
CSP: chloropropyltrimethoxysilane (CPS, Cl-(CH_2_)_3_-Si-(OCH_3_)_3_) and chloromethyltrimethoxysilane (CMS, Cl-CH_2_-Si-(OCH_3_)_3_)
CuSCN: copper thiocyanate
CV: cyclic voltammetry
DBC: dibenzocarbazole
DCTPA: 2,5-dichloroterephthalic acid
DFE: defect formation energy
DFT: density functional theory
DMSO: dimethyl sulfoxide
DOS: density of states
DPHH: 2,4-dichlorophenylhydrazine hydrochloride
DSSCs: dye-sensitized solar cells
*EA*: electron affinity
EAL: effective attenuation length
*E*_*bin*_: binding energy
*E*_*C*_: conduction energy
EDC: energy distribution curve
EDC: energy distribution curve
EDS: energy dispersive spectroscopy
*E*_*F*_: Fermi level
*E*_*F,n*_: electron quasi-Fermi level
*E*_*F,p*_: hole quasi-Fermi level
*E*_*g*_: bandgapband gap
*E*_*kin*_: kinetic energy
*E*_*P*_: pass energy
ETL: electron transporting layer
*E*_*V*_: valence energy
*E*_*VAC*_: vacuum level
F4TCNQ: 2,3,5,6-Tetrafluoro-tetracyanoquinodimethane
F6TCNNQ: 2,2′-(perfluoronaphthalene- 2,6-diylidene)­dimalononitrile
FBABF_4_: 4-fluorophenylammonium tetrafluoroborate
*FF*: fill factor
FTO: fluorine-doped tin oxide
GB: grain boundary
GGA: generalized gradient approximation
GI: grain interior
HAD: hexane-1,6-diamine
HATCN: 1,4,5,8,9,12-hexaazatriphenylenehexacarbonitrile
HAXPES: hard X-ray photoemission spectroscopy
HF: Hartree–Fock
HOMO: highest occupied molecular orbital
HS-UPS: high-sensitivity UPS
HTL: holr transporting layer
ICBA: Indene-C60-bisadduct
IDIS: induced density of interface states
IL: ionic liquid
IMFP: inelastic mean free path
*IP*: ionization energy
IPES: inverted PES
IPH: isophorone
ITO: indium tin oxide
*J*_*SC*_: short circuit current
KBF_4_: potassium tetrafluoroborate
KPFM: Kelvin probe force microscopy
KS: Kohn-*n*-Sham
LDA: local density approximation
LEDs: light emitting diodes
LUMO: lowest unoccupied molecular orbital
MHP: metal halide perovskite
m-MTDATA: 4,4′,4″-tris­[phenyl­(*m*-tolyl)­amino]­triphenylamine
Mo­(tfd-COCF_3_)_3_: Molybdenum tris­(1-(trifluoroacetyl)-2-(trifluoromethyl)­ethane-1,2-dithiolene)
MOF: metal–organic framework
MPP: maximum power point
*N*: Avogadro’s number
NAP: near-ambient pressure
NDI2HD: naphthalene-diimide
NDP: *P*-9–2-(7-dicyanomethylene-1,3,4,5,6,8,9,10-octafluoro-7H-pyrene-2-ylidene)-malononitrile
NIR: near-infrared
n-*n*-DMBI: 4-(2,3-Dihydro-1,3-dimethyl-1H-benzimidazol-2-yl)-N,N-*N*-dimethylbenzenamine
OAI: octylammonium iodide
OPV: organic photovoltaic
PAA: poly­(acrylic acid)
PBE: Perdew–Burke–Ernzerhof
PCBM: phenyl-C61-butyric acid methyl ester
PCE: power conversion efficiency
PEI: polyethylenimine
PEIE: ethoxylated polyethylenimine
PEO: poly­(ethylene oxide)
PES: photoemission sspectroscopy
PFN-*N*-Br: Poly­[(9,9-bis­(3′-((N,N-*N*-dimethyl)-N-*N*-ethylammonium)-propyl)-2,7-fluorene)-*alt*-2,7-(9,9-dioctylfluorene)]­dibromide
PFPT3: 5,5′-(2,5-difluoro-1,4-phenylene)­bis­(3-(2-decyltetradecyl)­thiophene)
PMAI: phenylmethylammonium iodide
PrC_60_MA: C60,N,N,N -trimethyl-1-(2,3,4-tris­(2-(2-methoxyethoxy)­ethoxy)­phenyl)­methanaminium monoadduct
PSC: perovskite solar cell
PTAA: Poly-[bis­(4-phenyl)-(2,4,6-trimethylphenyl)-amin]
QFLS: quasi-Fermi level splitting
rGO: graphene oxide
SAM: self-assembled monolayer
SCF: self-consistent field
SCs: solar cells
SECO: secondary electron cutoff
SHE: Schockley-Read-Hall
SOC: spin-*n*-orbit coupling
Spiro-OMeTAD: 2,2′,7,7′-Tetrakis­[N,N-*N*-di­(4-methoxyphenyl)­amino]-9,9′-spirobifluorene
SPV: surface photovoltage
SR: scalar relkativistic
s-SNOM: scattering-type Scanning Near-field Optical Microscopy
*t*: tolerance factor
TaTm: *N*4,*N*4,*N*4″,*N*4″-tetra­([1,1′-biphenyl]-4-yl)-[1,1′:4′,1′′-terphenyl]-4,4′′-diamine
TCO: transparent conductive oxide
TCTA: tris­(4-carbazoyl-9-ylphenyl)­amine
TEACl: 2-thiopheneethylammonium
TEM: transmission electron microscopy
TEM: transmission electron microscopy
TFET: tunneling field-effect transistors
TFSI: bis­(trifluoromethane)­sulfonimide
TFT: thin film transistor
TOPO: trioctylphosphine oxide
trSPV: time-resolved surface photovoltage
UHV: ultra high vacuum
UPS: Ultraviolet photoemission (or photoelectron) spectroscopy
UV: ultraviolet
VB: valence band
*V*_*bi*_: built-in potential
VBM: valence band maximum
*V*_*OC*_: open circuit voltage
W_2_(hpp)_4_: tetrakis­(hexahydropyrimidinopyrimidine)­ditungsten­(II)
*WF*: work function
XC: exchange-correlation
XPS: X-ray photoemission spectroscopy
XRD: X-ray diffraction
Δ*E*_*F*_: quasi-Fermi level splitting
τ_TPL,HLI_: carrier-density-dependent decay time
